# Automated Variable Electric-Field DFT Application for Evaluation of Optimally Oriented Electric Fields on Chemical Reactivity

**DOI:** 10.1021/acs.joc.2c01893

**Published:** 2022-12-12

**Authors:** Dalton J. Hanaway, C. Rose Kennedy

**Affiliations:** Department of Chemistry, University of Rochester, Rochester, New York14627, United States

## Abstract

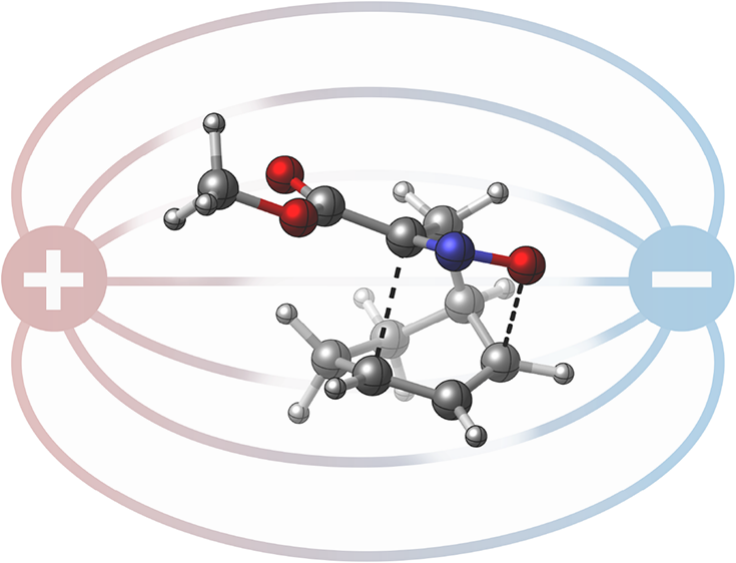

Recent theoretical work and experiments at molecular junctions have provided a strong conceptualization for the effects of oriented electric fields (OEFs) on organic reactions. Depending on the axis of application, OEFs can increase (or decrease) the reaction rate or distinguish between isomeric pathways. Despite the conceptual elegance of OEFs, which may be applied externally or induced locally, as tools for catalyzing organic reactions, implementation in synthetically relevant systems has been hampered by inefficiencies in evaluating reaction sensitivity to field effects. Herein, we describe the development of the Automated Variable Electric-Field DFT Application (A.V.E.D.A.) for streamlined evaluation of a reaction’s susceptibility to OEFs. This open-source software was designed to be accessible for nonexpert users of computational and programming tools. Following initiation by a single command (and with no subsequent intervention) the Linux workflow manages a series of density functional theory calculations and mathematical manipulations to optimize local-minimum and transition-state structures in oriented electric fields of increasing magnitude. The resulting molecular and reaction dipole moments, field-perturbed geometries, and net effective activation energies are compiled for user interpretation. Ten representative pericyclic reactions that showcase the development and evaluation of A.V.E.D.A. are described.

## 1 Introduction

Achieving precise control over reactivity and selectivity is a defining goal of synthetic chemistry. Traditionally, this has been achieved through substrate, reagent, or catalyst control under carefully tuned reaction conditions including (for example) solvent, concentration, temperature, and time. By contrast, external electromagnetic stimuli have traditionally been relegated to spectroscopic characterization with little consideration for potential effects on chemical transformations more broadly. In a disruption to these traditional approaches, oriented electric fields (OEFs) have been identified as an alternative synthetic tool.^1−4^ An applied electric field hyperpolarizes coaxial bonds, which in turn alters the effective barriers for transformations involving changes in bonding and redistribution of electron density associated.^4^ This phenomenon—called the electric-field effect—has long been invoked to justify the catalytic efficiency of enzymes,^5−11^ and spectroscopic work has quantified the field-induced component of enzymatic catalysis.^6,12−16^ Analogous techniques have been applied to probe effective electric-field magnitude and orientation at electrode surfaces and interfaces.^17−19^

**Figure 1:**
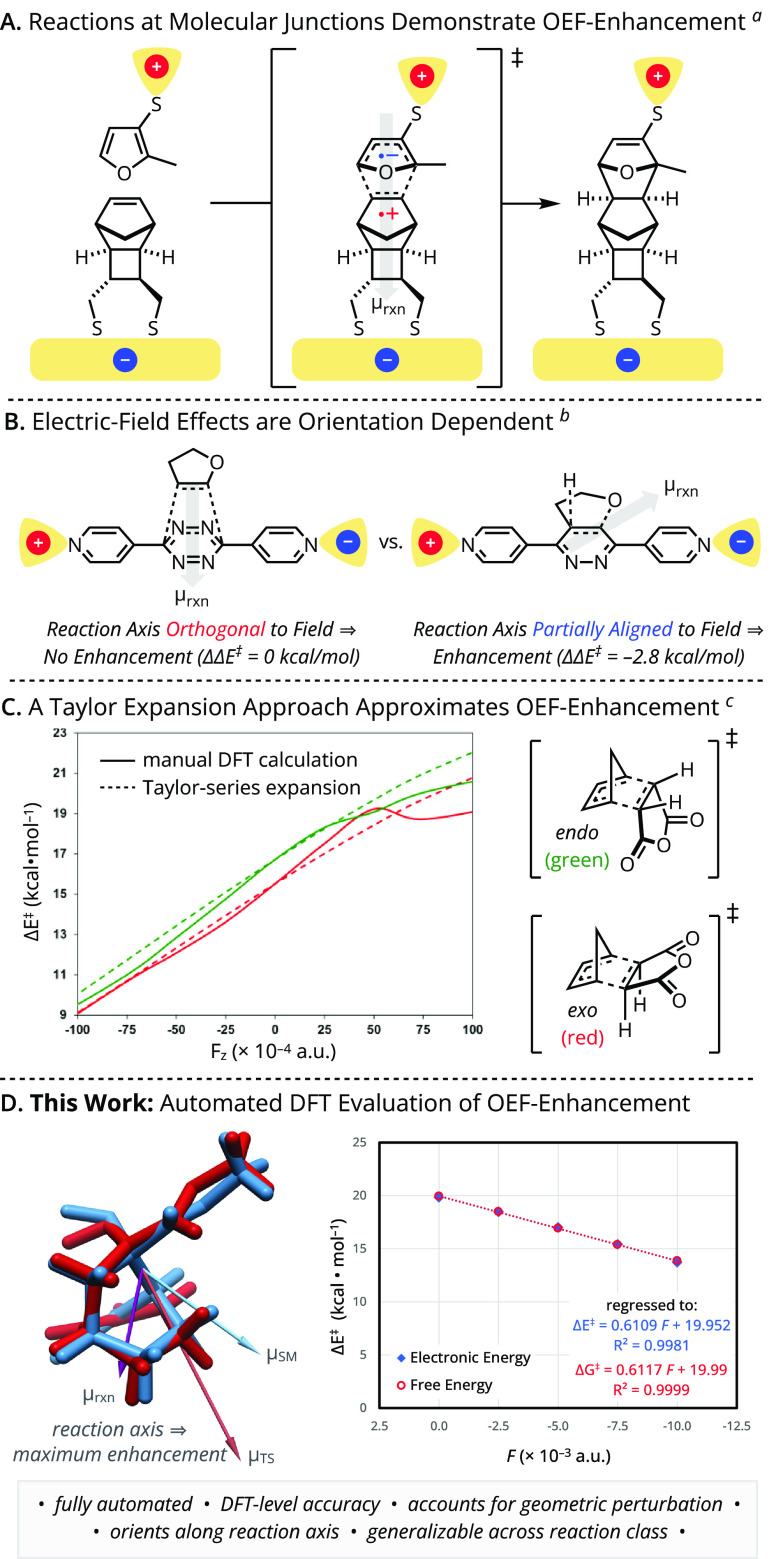
Foundational (A) conceptualization, (B) demonstration, and (C, D) approaches for expedited modeling of oriented electric field (OEF) effects on organic reactions. ^a^, ref (25); ^b^, ref (24); ^c^, ref (29). Plot modified with permission from Besalú-Sala, Solà, Luis, & Torrent-Sucarrat. 2021 American Chemical Society. For overlaid structures, blue = starting material local-minimum stationary point, red = lowest energy transition-state structure.

In nonenzymatic systems, theoretical studies, most commonly examining concerted cycloaddition reactions, support the viability of OEFs as unconventional “catalysts” for synthetically relevant transformations (Figure 1A).^20−23^ In agreement with computational predictions, single-molecule experiments examining Diels–Alder substrates immobilized at molecular junctions supported that C–C bond formation was accelerated or inhibited depending on the orientation of the applied electric field (Figure 1A,B).^24,25^ Increasingly, this approach has been expanded to evaluate field effects on a range of chemical transformations at molecular junctions.^26−28^ These studies further highlight sensitivity of OEF effects to alignment with the reaction axis, which is defined by the vector difference in electron localization between ground and transition states.^2,25^

Electric-field effects on molecular systems can be described mathematically through a power series where electronic energy (*E*) depends on the interaction of the electric field (*F⃗*) with the molecular dipole moment (μ⃗), polarizability (α⃗), hyperpolarizability (β⃗), etc.^29,30^
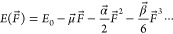
1

As a first approximation, the higher-order terms can be neglected to relate the interaction energy to the product of the field and molecular dipole moment.
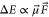
2

Because both μ⃗ and *F⃗* are vectors, the effect is the greatest when they are aligned parallel or antiparallel. For a chemical reaction within an electric field, the vector difference in dipole moments between the local-minimum and transition-state structures (Δμ⃗^‡^) therefore determines the overall change in energy barrier.
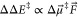
3

For additional introduction into the fundamental bases for electric-field effects on reactivity and their sensitivity to orientation, readers are referred to a tutorial review from Shaik and coworkers.^2,3^

The theoretical understanding and proof-of-principle results described above have inspired a burgeoning area of research that seeks to harness OEFs in synthetically practical systems. This has been pursued through design of molecular capacitors,^31,32^ electrostatically biased molecular frameworks, and especially small-molecule reagents or catalysts with an induced local electric field.^3,33,34^ Related work in inorganic and coordination chemistry has employed secondary coordination sphere electrostatics to alter redox thermochemistry and related processes.^35−43^ However, harnessing (and understanding) even local field effects for synthetically useful reactivity is hampered by the tedious and technically arduous computational process of evaluating both the viability (magnitude of the possible electric field effect) and optimal orientation of the applied field.

Herein, a user-friendly application is presented to facilitate density functional theory (DFT) calculations of chemical reactions in the presence of optimally oriented electric fields. The Automated Variable Electric-Field DFT Application (A.V.E.D.A.) provides the first fully automated electric field probe with DFT-level rigor. This approach is complementary to the Taylor-series expansion methodology developed by Luis et al.,^44^ which offers the trade-off between reduced precision and expedited computational efficiency when compared to DFT approaches (Figure 1C). Because each calculation with A.V.E.D.A. requires only a single command with simple user specifications that obviate the need for mathematical or programming expertise, this program is well-suited both as a teaching tool and as a prescreening tool for experimental electric field applications. Building from the marked OEF effects observed for Diels–Alder reactions, the development and application of A.V.E.D.A. are showcased with a suite of noncycloaddition pericyclic reactions as model transformations (Figure 1D).^45^ Predictions obtained with A.V.E.D.A. support the broad viability of energetically significant electric-field effects across these families of pericyclic processes, despite their limited sensitivity to traditional forms of catalysis. Additional extensions to mechanistically distinct test cases demonstrate excellent transferability of the developed method.

## 2 Results and Discussion

### 2.1 Input and Workflow

**Scheme 1:**
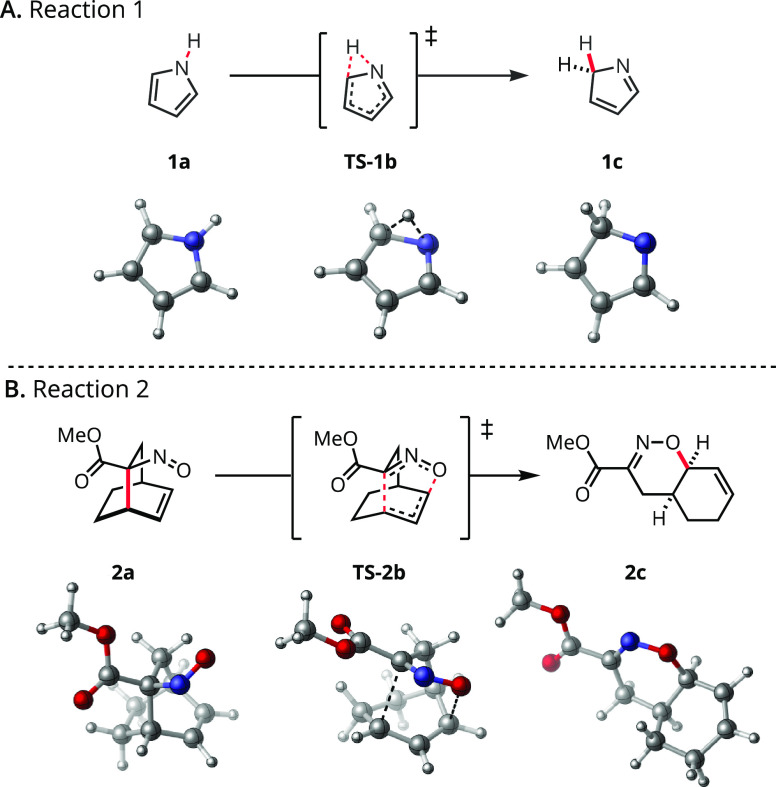
Select Model Reactions Used to Develop and Demonstrate A.V.E.D.A. Capabilities H = white, C = gray, N = blue, O = red.

To develop the workflow for A.V.E.D.A. and evaluate OEF results, pericyclic reactions were identified as an attractive model reaction class.^45^ Promising results obtained for the Diels–Alder reaction in an OEF suggested viability for a super-set of pericyclic reactions. Ten transformations—Cope elimination, Cope rearrangement, Claisen rearrangement, ene reaction, electrocyclic ring-opening/closing, and sigmatropic rearrangements ([1,5], [3,3], and [2,3])—were selected as the development data set in addition to the well-studied [4 + 2]-cycloaddition of cyclopentadiene and maleic anhydride.^20,22,44^ The small size and concerted mechanisms enabled quick computation times and efficient algorithm development while offering a range of electronic descriptions and activation energies. Two representative transformations are depicted in the main text to illustrate the capabilities and results obtained with A.V.E.D.A. (Scheme 1). The remainder are provided in the Supporting Information.

A.V.E.D.A. input consists of two *.xyz* files listing the coordinates for local-minimum and transition-state structures for the transformation of interest. The output consists of optimized structures and energies at zero field and four non-zero electric-field strengths. Note that the transition state describes a first-order saddle point on the ground-state potential energy surface, and the local minimum describes the nearest starting material or intermediate stationary point on the same energy surface. Upon instantiation, all subsequent OEF calculations proceed automatically with no user intervention required; see Computational Methods for details and the Supporting Information for workflow visualization. Nonetheless, the quality of the output depends on the input, and users are encouraged to perform a routine optimization at a low level of theory and validate the stationary points by frequency analyses for input structures prior to submission through A.V.E.D.A. For reactions that could conceivably react through multiple conformers and/or include multiple elementary steps, each case should be evaluated independently. For example, the input structures for the model reactions described in this manuscript were optimized initially with B3LYP/6-31 g(d) prior to submission to A.V.E.D.A. for evaluation at higher levels of theory.

Upon beginning each run, a directory containing copies of all necessary A.V.E.D.A. scripts is created. Local-minimum and transition-state structures are formatted into Gaussian input files for initial optimization in the absence of an applied electric field and submitted to SLURM for job handling.^46^ The Gaussian computational chemistry package was selected due to its user-friendly interface, broad adoption by the synthetic community, and track-record of implementation in prior computational reports investigating electric-field effects.^1,21,23,29,47−50^ Next, corresponding atoms in the optimized local-minimum and transition-state structures are aligned in the Cartesian coordinate system using PyMol, an open-source molecular visualization platform, via a root-mean-square deviation (RMSD) distance reduction.^51^

### 2.2 Reordering Atomic Coordinates

Subsequent calculations in an applied electric field utilizing the *Field* keyword must be performed on structures in Z-matrix format. In contrast to a Cartesian format, the Z-matrix constrains rotational degrees of freedom by constraining *atom 1* at the origin, *atom 2* on the *z*-axis, *atom 3* on the *x*-axis, and *atom 4* on the *y*-axis. These four atoms are referred to as the ″orientation atoms″ throughout this work. This constraint is necessary to prevent reorientation during the OEF optimization. During development, it became clear that the input order of atomic coordinates (and therefore the quality of the Z-matrix) influenced the success of subsequent OEF optimization steps. To support successful implementation, the atomic coordinates for aligned structures were thus reordered by one of three methods, described below.

- *Method 0* imposes no reordering criteria prior to implementation of the Gaussian *newzmat* utility. However, the *newzmat* utility may arbitrarily reorder the atomic coordinates if the input order is not conducive to Z-matrix construction.
- *Method 1* was developed to maximize the stability of the orientation atoms in an electric field by calculating the unweighted Cartesian center of the transition structure and moving the nearest three atoms to the orientation atom positions prior to implementation of the *newzmat* utility. It is predicted that these core atoms will have the smallest net displacement during optimization.
- *Method 2* reorders atoms to minimize impact of geometric changes on dipole orientation by designating the atom furthest from the site of transformation as orientation *atom 1* prior to implementation of the *newzmat*. This remote atom and all subsequent atoms are moved in a block to the top of the input file so that connectivity represented by atom order is preserved with minimal perturbation.

During testing on the pericyclic data set, it was determined that *method 1* was the most suitable when fringe atoms could easily rotate (e.g., a methyl group) or when the transition state was comprised of two unconnected fragments associated only by bond-breaking and forming (see the Supporting Information for details). *Method 2* proved useful when *method 1* produced a low-quality Z-matrix due to inconsistencies between atom order and molecular connectivity.

### 2.3 Dipole Moment Vector Algebra

**Figure 2:**
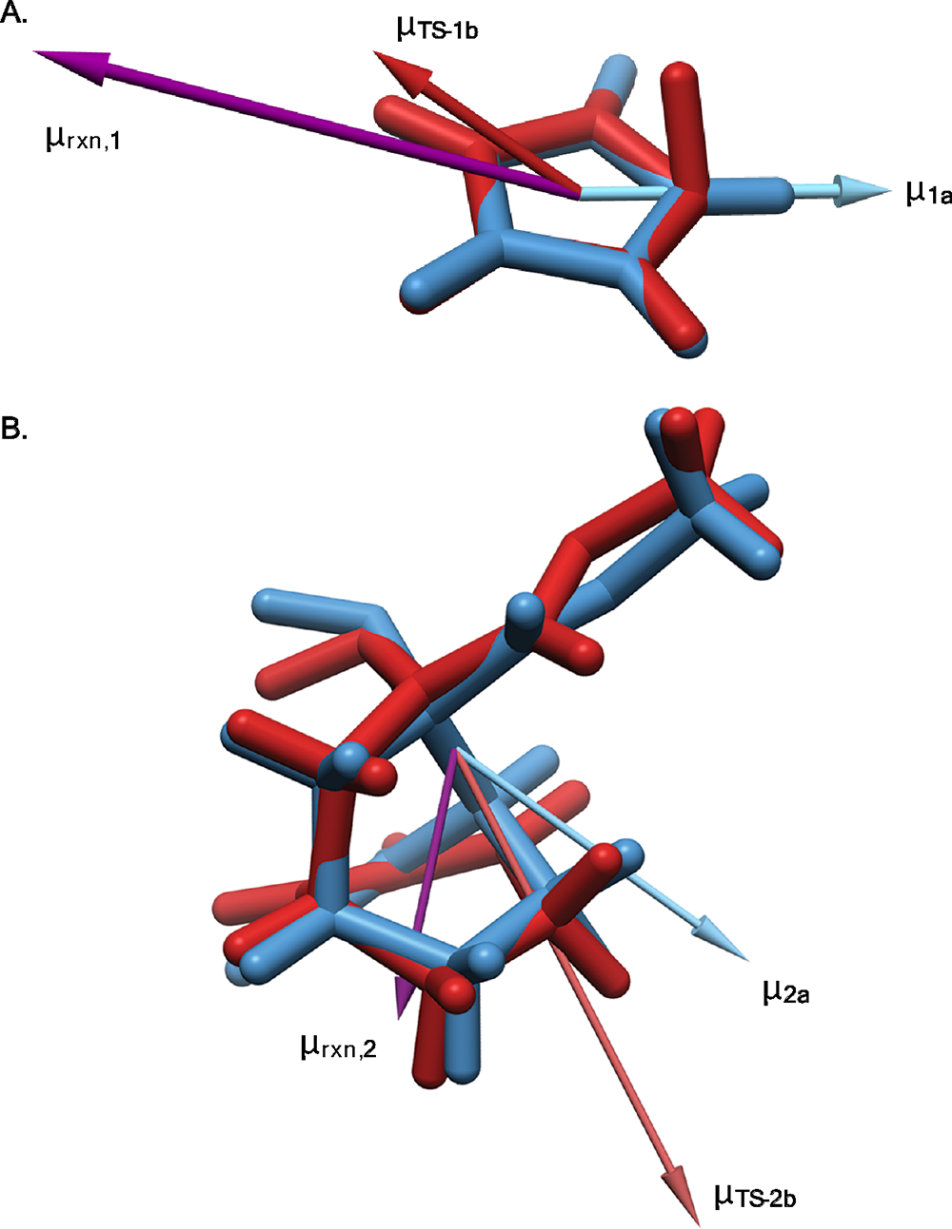
Structural alignment and vector algebra to determine the reaction dipole (μ⃗^‡^ or μ⃗_rxn_) and normalized reaction axis (μ̂^‡^) from the local-minimum (blue) and transition-state (red) structures for (A) reaction 1 and (B) reaction 2. The electric field is applied along the negative direction of μ̂^‡^.

Following alignment and reordering, single-point energy calculations are performed in the Cartesian and Z-matrix input formats for both the optimized local-minimum and transition-state structures. From these results, A.V.E.D.A. calculates the optimal electric-field alignment from the normalized dipole difference vector (μ⃗^‡^or μ⃗_rxn_) between the local-minimum (μ⃗_Int_) and transition-state (μ⃗_TS_) dipole moments (eq 4, Figure 2). This unit vector (μ̂^‡^), often described as the ″reaction axis″, points along the flow of electron density during a transformation and thus approximates the orientation most susceptible to beneficial electric field perturbation.^20^
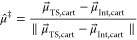
4

However, the local-minimum and transition-state structures change orientation independently when converted from Cartesian to Z-matrix coordinate systems. Therefore, μ̂^‡^ must be mapped between the two orientations for each structure. This process is accomplished by generating rotation matrices for the local-minimum and transition-state dipole moments (eqs 5–7). For each structure, the Cartesian μ̂_cart_^‡^ vector is translated with its corresponding rotation matrix (eq 7) into the Z-matrix coordinate system. The electric field is then applied along the resulting μ̂_zmat, SM_^‡^ or μ̂_zmat, TS_^‡^ for the local-minimum and transition-state structures, respectively.
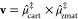
5
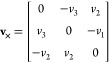
6
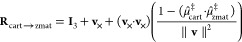
7

### 2.4 Job Submission and Output

Gaussian submission scripts are generated for geometry optimizations with applied electric fields oriented along μ̂_zmat_^‡^ and scaled by −2.5, −5.0, −7.5, and – 10.0 × 10^–3^ a.u. The structures optimized at zero-field (above) are used to generate the input geometry for the −2.5 × 10^–3^ a.u. case. Input geometries for subsequent optimizations at greater field strengths are called recursively from the preceding checkpoint files once optimization in the weaker field converges. This iterative approach improves the probability of successful computational outcomes by minimizing the geometric perturbation in any single step. After all eight OEF optimizations have converged, the output files are moved to the results directory for analysis. At this stage, the RMSD structural deviation is calculated for each optimized structure with respect to the unperturbed (zero-field) system and reported in the output. Deviations of the RMSD of >10% thus warn the users of possible changes to mechanism, fragmentation, and/or field-induced distortions.^21,48,49^

**Figure 3:**
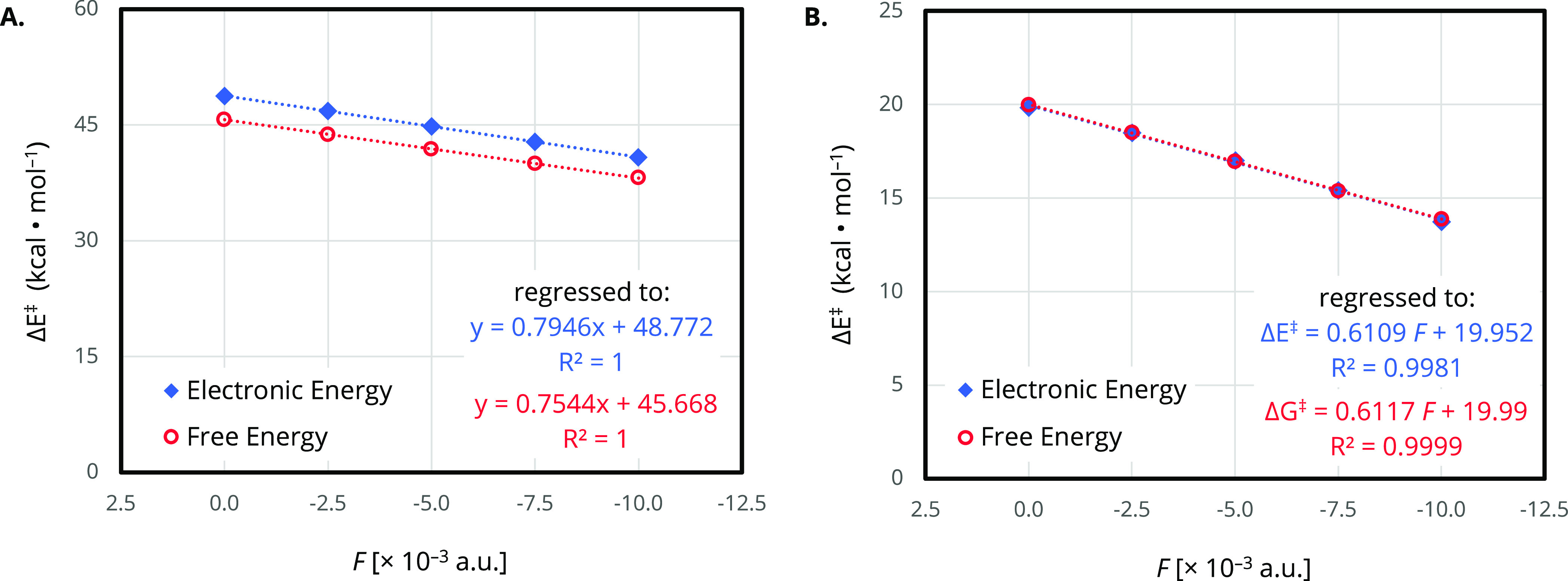
Plot of the effective activation energy (Δ*E*^‡^ or Δ*G*^‡^, kcal/mol) as a function of OEF magnitude for (A) reaction 1 and (B) reaction 2 computed at the B3LYP-D3/def2-TZVP level of theory (gas phase, 298 K). Both uncorrected electronic energies (blue diamonds) and corrected electronic and thermal free energies (red circles) are reported.

Using A.V.E.D.A., all 11 pericyclic reactions in the development data set were evaluated using four different functionals (B3LYP, B3LYP-D3, M06-2X, and ωB97X-D) and Weigend’s triple-ζ basis set, def2TZVP.^52,53^ The hybrid B3LYP functionals^54^ (with and without Grimme’s D3 empirical dispersion correction)^55^ and M06-2X^56^ (which accounts for dispersion intrinsically) were selected due to their computational efficiency and track-record of performance for pericyclic reactions.^57,58^ The ωB97X-D variation of pure functional B97 was also evaluated to consider alternative functional forms with long-range dispersion correction.^59,60^ For each case, A.V.E.D.A. produced outputs resembling those illustrated in Figure 3 for reaction 2 (see the Supporting Information). However, the success rate varied with respect to the atomic ordering method selected. A successful run was characterized by near-linear reductions in activation energy as a function of OEF magnitude—indicated by an *R*^2^ greater than 0.95 and no discontinuous jumps in energy or molecular geometry—with appropriate imaginary vibrational frequencies for intermediates (none) and transition states (one). Slight, continuous deviations from linearity can be expected from the higher order terms in eq 1. Atom-ordering methods were evaluated using B3LYP. *Method 1* yielded an 80% success rate, while *methods 0* and *2* were successful for 70% of the data set. *Method 0* (or the most successful alternative where *method 0* was not successful) was then applied for calculations with the other functionals. Because each method was suitable for different types of geometries, overall, the three methods covered all of the pericyclic reactions tested (see the Supporting Information for details).

For all successful executions, A.V.E.D.A. tabulates the molecular dipole moments (μ⃗) and optimized local-minimum and transition-state electronic and free energies in CSV files (compiled in Table 1 for reactions 1 and 2). The RMSD distortion from the zero-field geometry is also reported each OEF strength to quantify structural changes. Finally, A.V.E.D.A. plots the effective activation energy (Δ*E*^‡^ or Δ*G*^‡^, kcal/mol) as a function of OEF magnitude (a.u.) from the tabulated energies (Figure 3).

**Table 1:** Summary of A.V.E.D.A. Output Data for Reaction 1 and Reaction 2 Computed at the B3LYP-D3/def2-TZVP Level of Theory (Gas Phase, 298 K)

Reaction 1					
Dipole Moments (μ) [Debeye]					
μ_Int_[*x*,*y*,*z*]	[0.2481, −1.8900, −0.0889]			||μ_Int_||	1.9083
μ_TS_ [*x*,*y*,*z*]	[0.3813, 1.2430, 0.6952]			||μ_TS_||	1.4744
μ_rxn_ [*x*,*y*,*z*]	[0.1332, 3.1330, 0.7841]			||μ_r*x*n_||	3.2324
Oriented Electric Field (*F*) [× 10^–3^ a.u.]					
	0	–2.5	–5.0	–7.5	–10.0
Electronic Energies (*E*)					
Int [a.u.]	–210.2570	–210.2553	–210.2541	–210.2532	–210.2527
TS [a.u.]	–210.1791	–210.1806	–210.1825	–210.1848	–210.1874
Δ*E*^‡^[kcal/mol]	48.88	46.89	44.90	42.91	40.93
ΔΔ*E*^‡^[kcal/mol]	0	–1.99	–3.98	–5.97	–7.95
Free Energies (*G*)					
Int [a.u.]	–210.2011	–210.1996	–210.1985	–210.1978	–210.1974
TS [a.u.]	–210.1282	–210.1297	–210.1316	–210.1339	–210.1365
Δ*G*^‡^[kcal/mol]	45.75	43.85	41.95	40.07	38.22
ΔΔ*G*^‡^[kcal/mol]	0.00	–1.91	–3.80	–5.68	–7.54
RMSD from *Z*ero–Field [Å]					
Int		0.0023	0.0055	0.0092	0.0081
TS		0.0024	0.0049	0.0074	0.0098

Reaction 2					
Dipole Moments (μ) [Debeye]					
μ_Int_[*x*,*y*,*z*]	[−0.1313, −1.6860, 1.8263]			||μ_Int_||	2.4890
μ_TS_ [*x*,*y*,*z*]	[1.5291,–2.9343, 1.4541]			||μ_TS_||	3.6142
μ_rxn_ [*x*,*y*,*z*]	[1.6604,–1.2483,–0.3722]			||μ_rxn_||	2.1104
Oriented Electric Field (*F*) [× 10^–3^ a.u.]					
	0	–2.5	–5.0	–7.5	–10.0
Electronic Energies (*E*)					
Int [a.u.]	–669.5006	–669.5016	–669.5035	–669.5064	–669.5104
TS [a.u.]	–669.4689	–669.4721	–669.4764	–669.4819	–669.4885
Δ*E*^‡^[kcal/mol]	19.84	18.48	17.01	15.42	13.73
ΔΔ*E*^‡^[kcal/mol]	0.00	–1.36	–2.83	–4.42	–6.10
Free Energies (*G*)					
Int [a.u.]	–669.3190	–669.3200	–669.3221	–669.3253	–669.3298
TS [a.u.]	–669.2872	–669.2906	–669.2951	–669.3008	–669.3077
ΔG^‡^[kcal/mol]	19.97	18.49	16.93	15.38	13.88
ΔΔ*G*^‡^[kcal/mol]	0.00	–1.48	–3.04	–4.59	–6.09
RMSD from Zero–Field [Å]					
Int		0.0038	0.0074	0.0076	0.0190
TS		0.0195	0.0699	0.1121	0.1613

### 2.5 Validating the Reaction Axis Assumption

Following program workflow development and testing, the key assumption underlying this ersatz was validated by considering the electric-field orientation with respect to μ̂^‡^ for reaction 1. The A.V.E.D.A. algorithm was adapted to apply an electric field along 20 vectors distributed over a unit sphere, each scaled to a magnitude of 5.0 × 10^–3^ a.u. The field-perturbed local-minimum and transition-state structures and energies were then computed. As expected, the lowest Δ*E*^‡^ was realized along −μ̂^‡^, while fields that increased Δ*E*^‡^ were found when the applied field included a +μ̂^‡^component; see the Supporting Information for visualization. Note that the Gaussian positive field direction is opposite to physics convention, thus applying an electric field along −μ̂^‡^, represents alignment between the field and reaction axis of the given transformation and stabilization of the dipole moment.^13^ These data thus validated the assumption, made in developing the program workflow, that the optimally oriented fields project along μ̂^‡^.

### 2.6 Relating OEF Effects to Dipoles and Geometries

**Figure 4:**
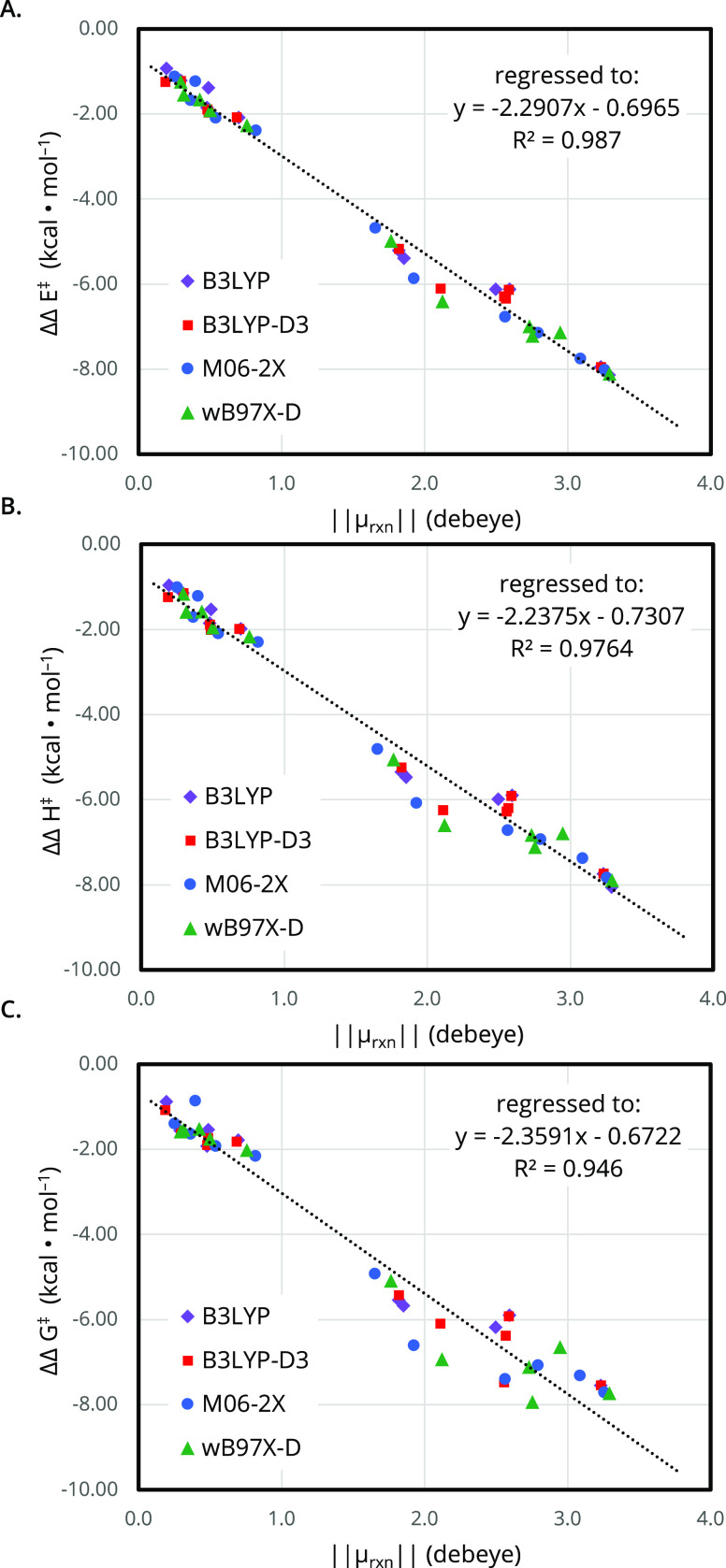
Correlation between net reaction dipole moment (μ⃗^‡^ or μ⃗_rxn_) and change in effective activation energy for the model reaction set described by the (A) uncorrected electronic energy difference (ΔΔE^‡^), (B) thermally corrected enthalpy difference (ΔΔ*H*^‡^), and (C) thermally corrected free energy difference (ΔΔ*G*^‡^). Results were computed in the gas phase at 298 K using B3LYP (purple diamonds), B3LYP-D3 (red squares), M06-2X (blue circles), and ωB97X-D (green triangles) with the def2-TZVP basis set at 0 vs 10 × 10^–3^ a.u.

A relationship between the predicted rate enhancement and the magnitude of Δμ⃗^‡^ emerged from the pericyclic reaction data set. Across levels of theory, the change in activation energy OEF (vs the zero-field case) with application of an oriented electric field (10.0 × 10^–3^ a.u.) demonstrated a strong linear correlation with the reaction dipole magnitude (Figure 4), thus implying that reactions with larger ∥μ⃗^‡^∥ are more susceptible to acceleration by an OEF. The strongest correlation (*R*^2^ = 0.987) was observed with the electronic energy difference (ΔΔ*E*^‡^, Figure 4A), with only a slightly weaker fit (*R*^2^ = 0.9764) observed when considering the computed enthalpy difference (ΔΔ*H*^‡^, Figure 4B). However, a significantly increased scatter (*R*^2^ = 0.946), especially involving cases with large ∥μ⃗^‡^∥, was observed upon considering the computed free energy difference (ΔΔ*G*^‡^, Figure 4C). This result arises from the entropic impacts of field-induced geometric changes, which may be nonintuitive to predict. This variable behavior thus highlights the utility of full DFT-level evaluation of a reaction of interest, rather than considering the reaction dipole difference alone.

**Figure 5:**
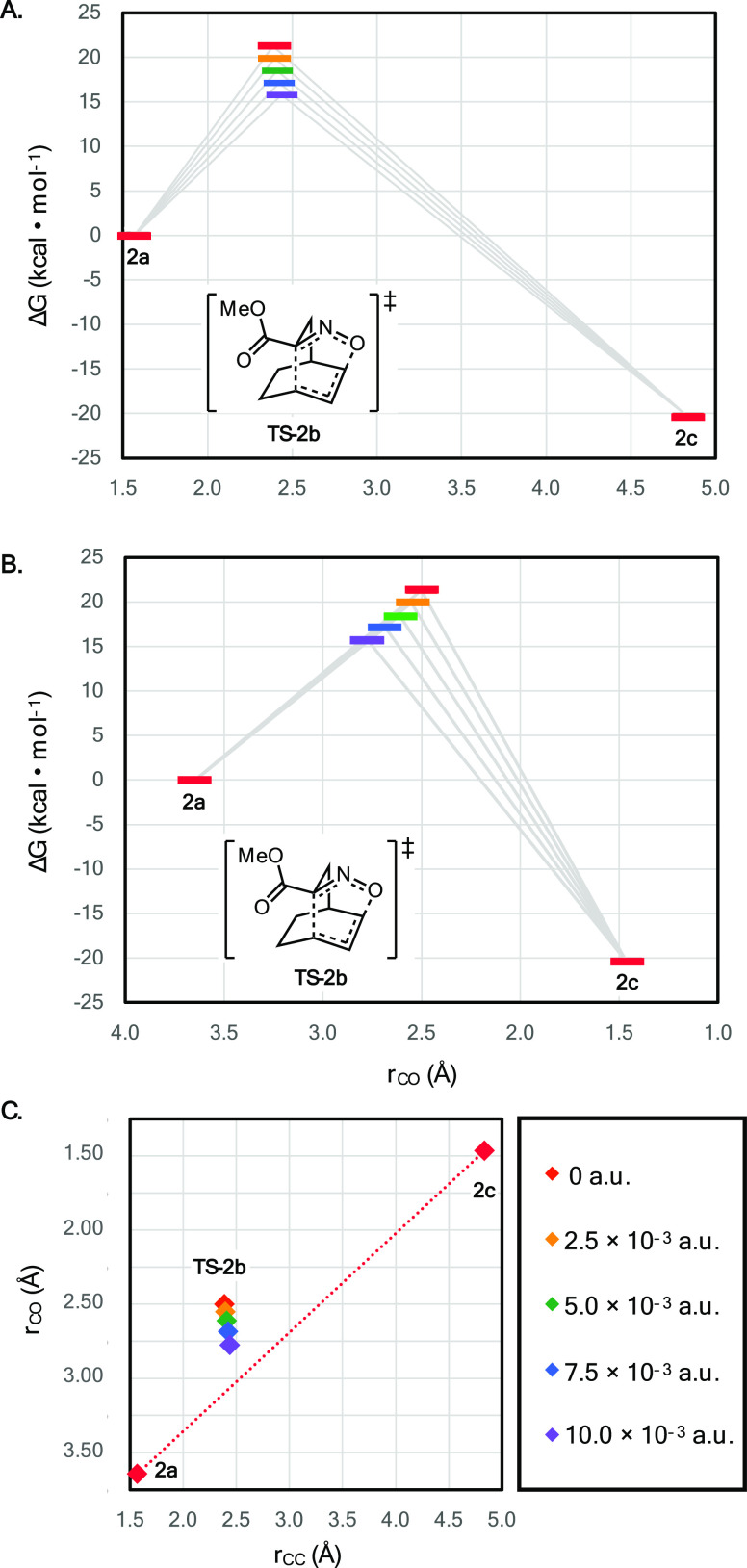
Field-induced geometric distortions in the transition-state structure for reaction 2 represented as reaction coordinate diagrams reflecting changing (A) C–C and (B) C–O bond lengths as well as (C) a More O’Ferrall–Jenks representation. Calculations performed at the B3LYP-D3/def2-TZVP level of theory (gas phase, 298 K). Zero-field (red) and applied fields of 2.5 (orange), 5.0 (green), 7.5 (blue), and 10.0 (purple) × 10^–3^ shown.

To better understand the nature of the geometric changes arising from application of the electric field, molecular geometries were thus evaluated for reaction 2 as a case study. Although geometric perturbations were observed in the presence of an applied field for both the local-minimum and transition-state structures, the effects in the transition state were substantially greater, reflected in slight lengthening of the breaking C–C bond (Figure 5A, Δ*r*_CC_ = +0.05 Å) and substantial lengthening of the forming C–O bond (Figure 5B, Δ*r*_CO_ = +0.274 Å) over the 0–10 × 10^–3^ a.u. range. Taken together, these geometric changes reflect a shift toward an earlier, less-associative transition state, as depicted by the trend toward the diagonal in the More O’Ferrall–Jenks plot (Figure 5C).^61^ These findings further highlight that one of the strengths of a full DFT-optimization for evaluating electric field effects (as compared to the rapid, Taylor series expansion approximation^44^) is the wealth of resulting structural insight.

### 2.7 Scope of A.V.E.D.A

Although A.V.E.D.A. was developed and tested on the pericyclic reaction training data set, its design facilitates broad applications. To demonstrate transferability, A.V.E.D.A. was implemented to study potential electric field effects on alternative (nonpericyclic) reaction classes including electrophilic addition and concerted metallation–deprotonation (CMD) mechanisms (see the Supporting Information).^62^ Computations have no constraint on system size, computer resource caps, or run time limits (barring those controlled by SLURM for the user’s computing cluster). While optimization times depend on system size, the local A.V.E.D.A. calculations and processes remain virtually unchanged. Furthermore, multiple predefined atom ordering methods, variable level of theory, detailed documentation, and fully open-source software enable broad customization for computationally challenging instances. The simple initiation requirements and automated mathematical processing thus increase the accessibility of OEF studies to students and computational nonexperts, thereby providing opportunities for increased contribution from the synthetic community.

## 3 Conclusions

The computational workflow described herein offers a simple tool for evaluation of chemical reactions in optimally oriented electric fields using density functional theory. A.V.E.D.A. is controlled by a one-time command, making the program highly accessible to computational nonexperts. Results are discussed for a set of pericyclic reaction case studies, for which significant barrier reduction is predicted as a function of electric fields application along the μ⃗^‡^ dipole difference vector. Structural changes due to the electric fields are considered, in addition to the relationship between dipole moment magnitude and absolute barrier reduction. These insights bolster the promise of OEF applications to modulate chemical reactivity and improve accessibility of computational tools to support this effort.

## 4 Experimental

### 4.1 Computational Methods

The complete workflow for all A.V.E.D.A. processes was developed in Python 3 and Bash script intended for use on a computing cluster running a Linux operating system and the SLURM scheduling protocol.^46^ The Gaussian 16 module is loaded and executed for all density functional theory calculations throughout the workflow.^63^ Structural analysis, visualization, and presentation was facilitated by Avogadro,^64,65^ UCSF Chimera,^66^ and CYLview.^67^ Optimizations were completed in the gas phase at 298 K and were evaluated using four different functionals (B3LYP, B3LYP-D3, M06-2X, and ωB97X-D)^54−56,59,60^ and Weigend’s triple-ζ basis set, def2-TZVP.^52,53^ However, users may request any Gaussian-standard functional at instantiation. All reported structures were confirmed with normal vibrational mode analyses yielding zero imaginary frequencies for local minima and a single imaginary frequency for transition states. Uncorrected electronic energies and corrected thermal and electronic free energies are reported. The complete pericyclic data set geometries—optimized at all field strengths for each successful atom ordering method—are found in the Supporting Information.

### 4.2 Implementation

Each instance of A.V.E.D.A. requires intermediate and transition state geometries in *.xyz* format, with the corresponding atoms numbered consistently, to be in the same directory as the *start.sh* script and program folder. When called, this script handles all setup and execution of the A.V.E.D.A. workflow and thus must be provided with job-specific user arguments. First, charge and multiplicity must be entered corresponding to the overall charge and electron configuration of the given transformation. The desired level of theory is indicated with a functional and basis set argument as well as the method for atom reordering (see Results and Discussion for details). Computing resources are allocated from SLURM by the desired number of processors and compute node. Finally, the user specifies to output electronic energies only (keyword *nofreq*) or vibrationally corrected free energies (keyword *freq,* recommended). In the event that users bypass the frequency analysis to increase computational efficiency at the optimization stage, users are strongly encouraged to implement ex post facto frequency calculations and/or intrinsic reaction coordinate analyses. These are necessary to validate the stationary points identified and obtain physically meaningful corrected enthalpy and free-energy terms. Note, all input parameters must be satisfied or A.V.E.D.A. will return an error and stop instantiation. A.V.E.D.A. may be installed, edited, modified, and distributed, protected under the open source MIT License, from the Kennedy laboratory GitHub repository page.^68^

## Supporting Information Available

Supplementary figures; output and energies for all model reaction; and extended discussion (PDF)
Coordinates of computed structures (XYZ)

## Supplementary Material

jo2c01893_si_001.pdf

jo2c01893_si_002.xyz
