# Supplementary material for: Automated Variable Electric-Field DFT Application for Evaluation of Optimally Oriented Electric Fields on Chemical Reactivity
Source: J Org Chem. 2022 Dec 12;88(1):106–15. doi: 10.1021/acs.joc.2c01893 (PMC9830642; doi:10.1021/acs.joc.2c01893)
Supplement: Supplementary file 1 — jo2c01893_si_001.pdf [file jo2c01893_si_001.pdf]

Supporting Information

An Automated Variable E-Field DFT Application (A.V.E.D.A.) for Evaluation of Optimally Oriented Electric Fields on Chemical Reactivity

Dalton J. Hanaway and C. Rose Kennedy*

Department of Chemistry, University of Rochester

Rochester, New York 14627, United States

* c.r.kennedy@rochester.edu

Table of Contents for the Supporting Information

1. **General Information** **S3**
   - **1.1** Computational Methods S3
   - **1.2** Implementation S3
   - **1.3** Reaction Data Set S4
   - **1.4** Abbreviations S5
2. **Validation of Optimal Orientation Calculations** **S6**
3. **Atom Reordering Methods** **S7**
4. **A.V.E.D.A. Output & Key Results** **S8**
   - **4.1** Reaction 1 S8
   - **4.2** Reaction 2 S16
   - **4.3** Reaction 3 S24
   - **4.4** Reaction 4 S32
   - **4.5** Reaction 5 S40
   - **4.6** Reaction 6 S48
   - **4.7** Reaction 7 S56
   - **4.8** Reaction 8 S64
   - **4.9** Reaction 9 S72
   - **4.10** Reaction 10 S80
   - **4.11** Reaction 11 S88
   - **4.12** Reaction 12 (Acetylene Dibromination) S96
   - **4.13** Reaction 13 (Palladium-Mediated C–H Activation) S104
   - **4.14** Summary of Results by Functional S112
5. **References** **S124**

1. General Information

1.1 Computational Methods

The complete workflow for all A.V.E.D.A. processes was developed in Python 3 and Bash script intended for use on a computing cluster running a Linux operating system and the SLURM scheduling protocol.[1] Development and all calculations were performed on the BlueHive computing cluster managed by the University of Rochester Center for Integrated Research Computing (CIRC). DFT calculations were performed using Gaussian 16.[2] Alignment procedures and RMSD calculations were performed using PyMol (Version 2.3.0).[3] Structural analysis and visualization were performed using Avogadro,[4, 5] UCSF Chimera,[6] and CYLview.[7] Optimizations were completed in the gas phase at 298 K using were evaluated using four different functionals (B3LYP, B3LYP-D3, M06-2X, and ωB97X-D) [8-12] and Weigend's triple-ζ basis set, def2-TZVP.[13, 14] All reported structures were confirmed with normal vibrational mode analyses yielding zero imaginary frequencies for local minima and a single imaginary frequency for transition states.

1.2 Implementation

Each instance of A.V.E.D.A. requires input local-minimum and transition-state geometries in XYZ file format, in the same directory as the *start.sh* script and *Program* folder. When called, this script handles all setup and execution of the A.V.E.D.A. workflow (Figure S1) and thus must be provided with the following job-specific user arguments:

- Charge and multiplicity corresponding to the overall molecular charge and electron configuration
- The desired level of theory (functional and basis set arguments)
- The method for atom reordering (see Section 3).
- The desired number of processors and compute node for resource allocation by SLURM.
- A selection for electronic energies output only (keyword *nofreq*) or vibrationally corrected free energies (keyword *freq*).

Note, all input parameters must be satisfied or A.V.E.D.A. will return an error and stop instantiation.

A.V.E.D.A. may be installed, edited, modified, and distributed, protected under the open-source MIT License, from the Kennedy laboratory GitHub repository page.[15]

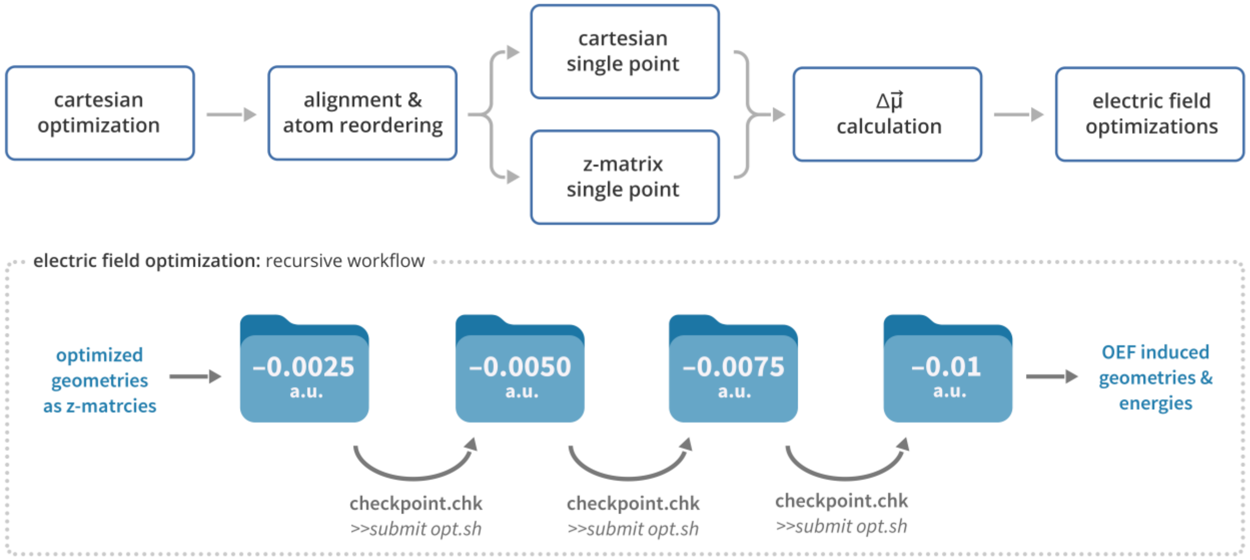

Figure S1. Overview of a processes during an A.V.E.D.A instance.

1.3 Reaction Data Set

To develop the A.V.E.D.A. workflow and evaluate OEF results, pericyclic reactions were identified as an attractive model reaction class. In addition to the well-studied Diels–Alder reaction between cyclopentadiene and maleic anhydride, ten non-cycloaddition reactions—Cope elimination, Cope rearrangement, Claisen rearrangement, ene reaction, electrocyclic ring-opening/closing, and sigmatropic rearrangements ([1,5], [3,3], and [2,3])—were selected for the development data set (Figure S2). The small size and concerted mechanisms enabled quick computation times and efficient algorithm development while offering a range of electronic descriptions. and activation energies. Reactions 1 and 2 are discussed in the main text as representative transformations; the remainder are provided below. Two additional, mechanistically distinct reactions (Reactions 12 and 13) were also evaluated to test for program generality.

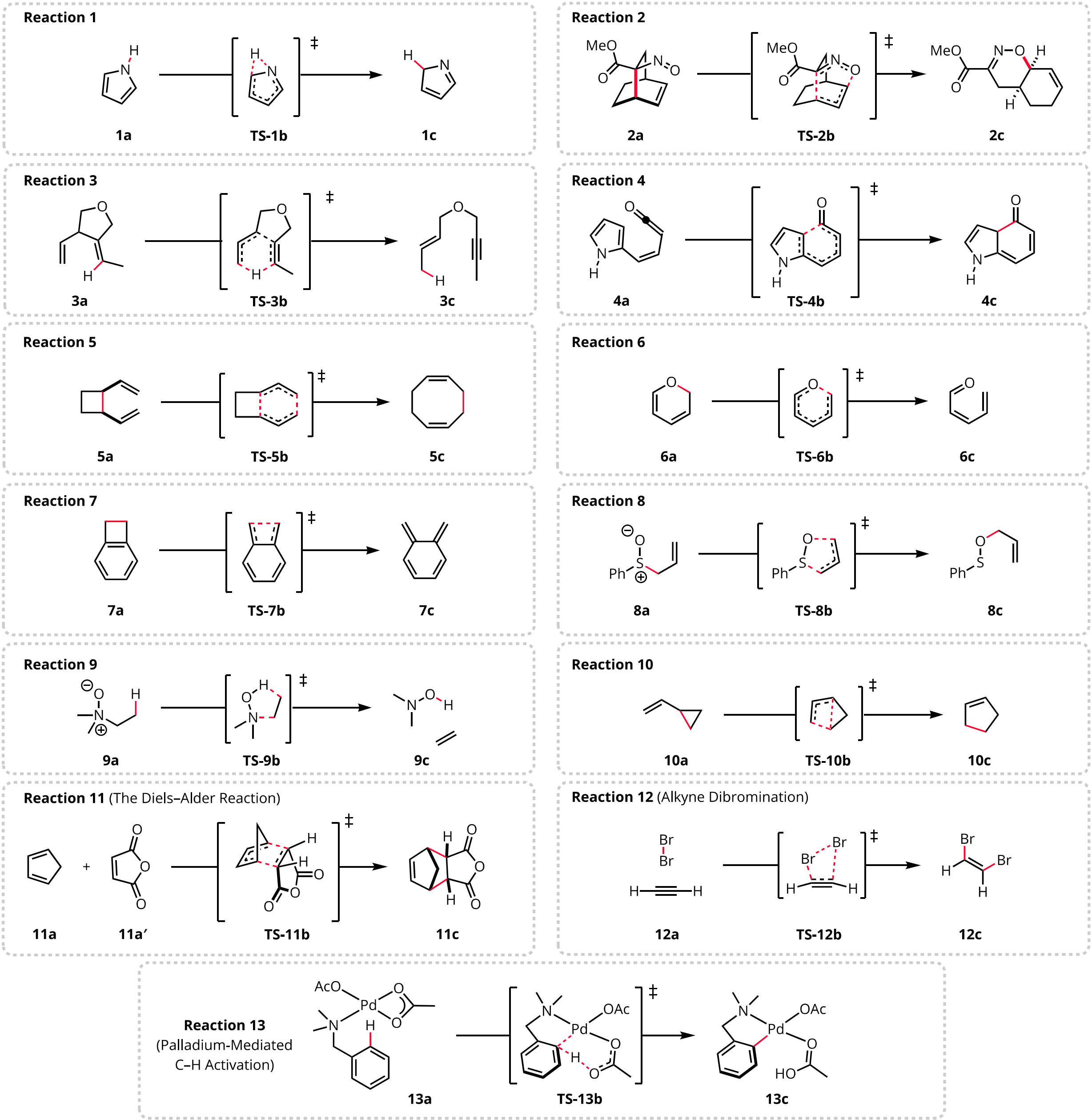

Figure S2. Summary of model reactions evaluated for development and testing of A.V.E.D.A.

1.4 Abbreviations

a.u. = atomic units; A.V.E.D.A. = automated variable-electric-field DFT application; CMD = concerted metallation deprotonation; CSV = comma-separated values (file format); DFT = density functional theory; OEF = oriented electric field; RMSD = root-mean-square deviation; XYZ = Cartesian coordinate (file format)

2. Validation of Optimal Orientation Calculations

A.V.E.D.A. calculates the optimal electric field alignment from the normalized dipole difference vector ($\vec{\mu}^{\ddagger}$or $\vec{\mu}_{rxn}$) between the local-minimum ($\vec{\mu}_{Int}$) and transition-state ($\vec{\mu}_{TS}$) dipole moments (eq. S1). This vector, often described as the "reaction axis", is depicted for Reactions 1 and 2 in Figure 2 (main text).

$$\hat{\mu}^{\ddagger} = \frac{\vec{\mu}_{TS,cart}-\vec{\mu}_{SM,cart}}{\|\vec{\mu}_{TS,cart}-\vec{\mu}_{SM,cart}\|} \quad \text{(S1)}$$

This key assumption was validated by considering the electric field orientation with respect to $\hat{\mu}^{\ddagger}$ for Reaction 1. The A.V.E.D.A. algorithm was modified to apply an electric field along 20 vectors distributed over a unit sphere, including the $\pm\hat{\mu}^{\ddagger}$ directions, each with a magnitude of 5.0×10^{-3} a.u. The field effects on the activation energy were normalized relative the zero-field case and multiplied by the unit vector along which the respective OEF was applied to visualize the effects (Figure S3). The lowest activation energy—corresponding to the longest dark blue vector—was realized along $-\hat{\mu}^{\ddagger}$ while fields increasing the transformation barrier were found in the opposite direction (with a $+\hat{\mu}^{\ddagger}$ component.)

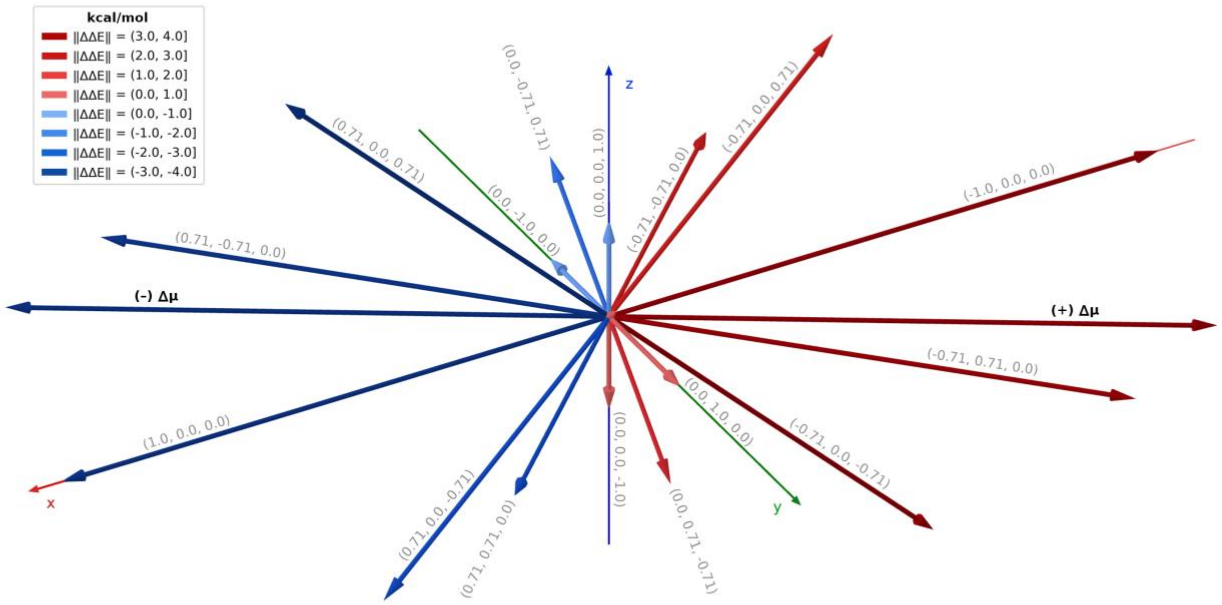

Figure S3. Vector magnitude and color represent the change in effective activation energy ($\Delta\Delta E^{\ddagger}$) in kcal/mol upon application of an oriented electric field (5.0×10^{-3} a.u.) along that vector's direction (gray coordinates). Blue shading indicates a more negative $\Delta\Delta E^{\ddagger}$ while red shading indicates a more positive $\Delta\Delta E^{\ddagger}$.

3. Atom Reordering Methods

Electric field calculations were performed in the Z-matrix format. However successful convergence to physically meaningful structures was found to be highly sensitive to the quality of the Z-matrix constructed from the input Cartesian coordinate data. To support successful implementation, the atomic coordinates for aligned structures were reordered by one of three methods. Because each method was suitable for different types of geometries, overall the three methods covered all of the pericyclic reactions tested (Table S1).

- Method 0 imposes no reordering criteria prior to implementation of the Gaussian *newzmat* utility. However, the *newzmat* utility may arbitrarily reorder the atomic coordinates if the input order is not conducive to z-matrix construction.
- Method 1 was developed to maximize the stability of the orientation atoms in an electric field by calculating the unweighted Cartesian center of the transition structure and moving the nearest three atoms to the orientation atom positions. It is assumed that these core atoms will have the smallest net displacement during optimization.
- Method 2 reorders atoms to minimize impact of geometric changes on dipole orientation by designating the atom furthest from the site of transformation as orientation atom 1. This remote atom and all subsequent atoms are moved in a block to the top of the input file so that connectivity represented by atom order is preserved with minimal perturbation.

Table S1. Change in effective activation energy ($\Delta\Delta E^{\ddagger}$) upon application of an oriented electric field (10.0×10^{-3} a.u.) predicted using different atomic ordering methods.

Reaction Index	$\Delta\Delta E^{\ddagger}$ (kcal mol^{-1}) [a]		
	Method 0	Method 1	Method 2
1	–7.9452	–7.9452	–7.8910
2	–5.2021	--	--
3	--	–1.3925	–1.9680
4	–6.1202	–5.7293	–5.0451
5	–1.8541	–1.1671	–0.7680
6	–2.0819	–1.5486	–1.9011
7	–1.2147	--	–1.2149
8	--	–5.3893	--
9	--	–6.1209	--
10	–0.9285	–0.9328	–1.2682
Success Rate	70%	80%	70%

[a] Electronic energies computed in the gas phase at 298 K at the B3LYP/def2-TZVP level of theory.

4. A.V.E.D.A. Output & Key Results

4.1 Reaction 1

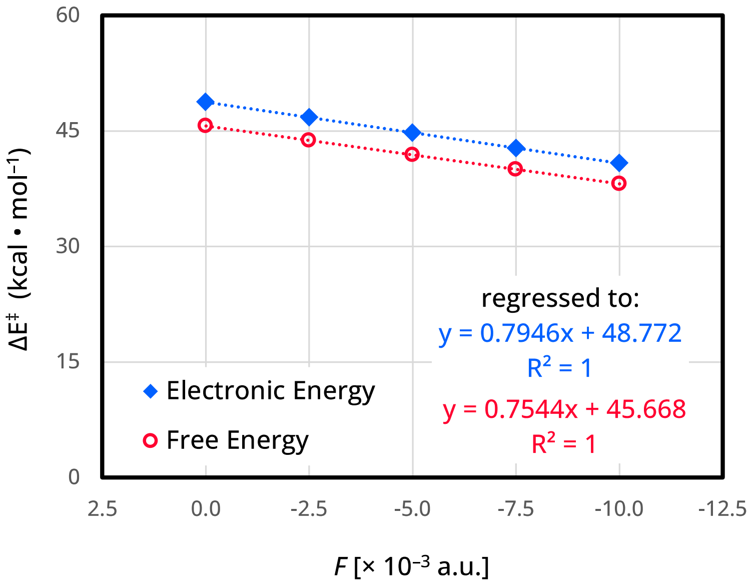

Figure S4. Effective activation energy ($\Delta E^{\ddagger}$ or $\Delta G^{\ddagger}$, kcal/mol) as a function of OEF magnitude for Reaction 1 computed at the B3LYP/def2-TZVP level of theory (gas phase, 298 K).

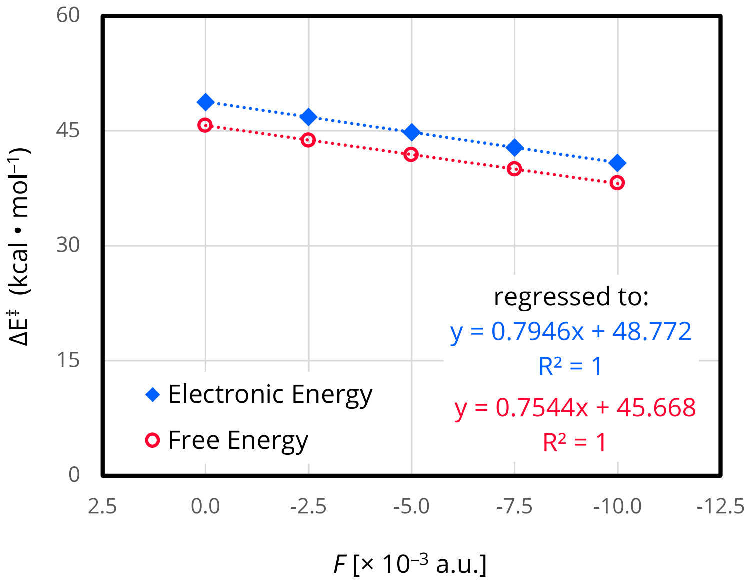

Figure S5. Effective activation energy ($\Delta E^{\ddagger}$ or $\Delta G^{\ddagger}$, kcal/mol) as a function of OEF magnitude for Reaction 1 computed at the B3LYP-D3/def2-TZVP level of theory (gas phase, 298 K).

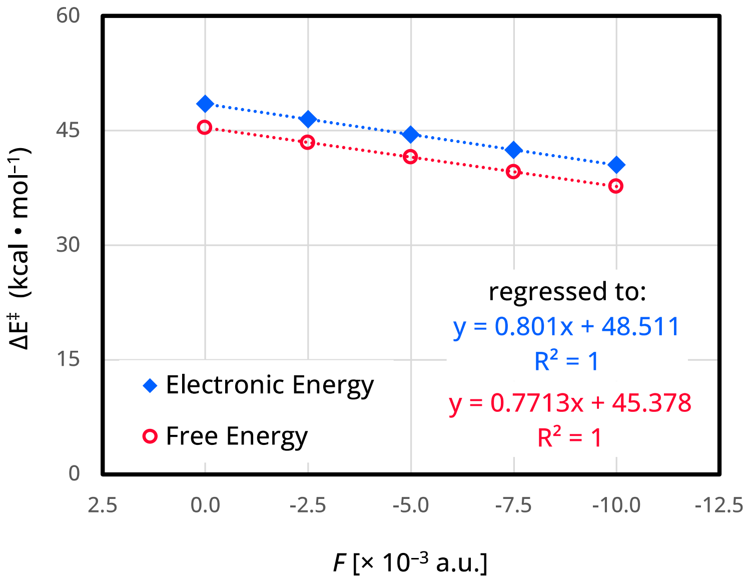

Figure S6. Effective activation energy (Δ$E^‡$ or Δ$G^‡$, kcal/mol) as a function of OEF magnitude for Reaction 1 computed at the M06-2X/def2-TZVP level of theory (gas phase, 298 K).

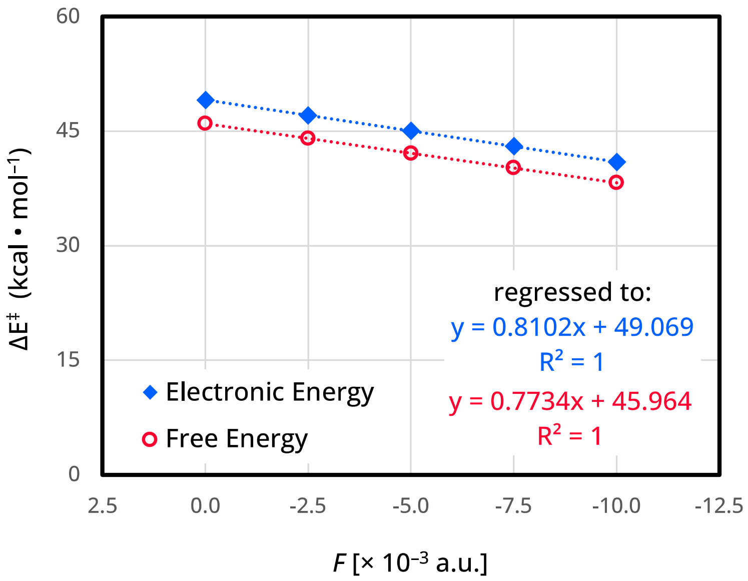

Figure S7. Effective activation energy (Δ$E^‡$ or Δ$G^‡$, kcal/mol) as a function of OEF magnitude for Reaction 1 computed at the ωB97X-D/def2-TZVP level of theory (gas phase, 298 K).

Table S2. Summary of A.V.E.D.A. output data for Reaction 1 computed at the B3LYP/def2-TZVP level of theory (gas phase, 298 K).

**Dipole Moments (debeye)**					
μ(Int) (x,y,z)	[0.2358, -1.8921, -0.0767]			‖μ(Int)‖	1.9083
μ(TS) (x,y,z)	[0.3881, 1.2417, 0.6959]			‖μ(TS)‖	1.4754
μ(rxn) (x,y,z)	[0.1523, 3.1338, 0.7726]			‖μ(rxn)‖	3.2312

**Oriented Electric Field**					
F [× 10^{-3} a.u.]	0	-2.5	-5	-7.5	-10
**Electronic Energies**					
Int [a.u.]	-210.2534	-210.2518	-210.2506	-210.2497	-210.2491
TS [a.u.]	-210.1757	-210.1773	-210.1792	-210.1814	-210.1841
$\Delta E^{\ddagger}$ [kcal/mol]	48.78	46.78	44.79	42.81	40.83
$\Delta\Delta E^{\ddagger}$ [kcal/mol]	0.00	-1.99	-3.98	-5.97	-7.95
**Free Energies**					
Int [a.u.]	-210.1976	-210.1960	-210.1949	-210.1942	-210.1939
TS [a.u.]	-210.1248	-210.1263	-210.1282	-210.1304	-210.1331
$\Delta G^{\ddagger}$ [kcal/mol]	45.69	43.77	41.88	40.00	38.14
$\Delta\Delta G^{\ddagger}$ [kcal/mol]	0.00	-1.91	-3.81	-5.69	-7.54
**RMSD from Zero-Field**					
Int (Å)	-	0.0023	0.0055	0.0091	0.0081
TS (Å)	-	0.0024	0.0048	0.0073	0.0098

Table S3. Summary of A.V.E.D.A. output data for Reaction 1 computed at the B3LYP-D3/def2-TZVP level of theory (gas phase, 298 K).

**Dipole Moments (debeye)**					
μ(Int) (x,y,z)	[0.2481, -1.89, -0.0889]			‖μ(Int)‖	1.9083
μ(TS) (x,y,z)	[0.3813, 1.243, 0.6952]			‖μ(TS)‖	1.4744
μ(rxn) (x,y,z)	[0.1332, 3.133, 0.7841]			‖μ(rxn)‖	3.2324

**Oriented Electric Field**					
F [× 10^{-3} a.u.]	0	-2.5	-5	-7.5	-10
**Electronic Energies**					
Int [a.u.]	-210.2570	-210.2553	-210.2541	-210.2532	-210.2527
TS [a.u.]	-210.1791	-210.1806	-210.1825	-210.1848	-210.1874
$\Delta E^{\ddagger}$ [kcal/mol]	48.88	46.89	44.90	42.91	40.93
$\Delta\Delta E^{\ddagger}$ [kcal/mol]	0	-1.99	-3.98	-5.97	-7.95
**Free Energies**					
Int [a.u.]	-210.2011	-210.1996	-210.1985	-210.1978	-210.1974
TS [a.u.]	-210.1282	-210.1297	-210.1316	-210.1339	-210.1365
$\Delta G^{\ddagger}$ [kcal/mol]	45.75	43.85	41.95	40.07	38.22
$\Delta\Delta G^{\ddagger}$ [kcal/mol]	0	-1.91	-3.8	-5.68	-7.54
**RMSD from Zero-Field**					
Int (Å)	-	0.0023	0.0055	0.0092	0.0081
TS (Å)	-	0.0024	0.0049	0.0074	0.0098

Table S4. Summary of A.V.E.D.A. output data for Reaction 1 computed at the M06-2X/def2-TZVP level of theory (gas phase, 298 K).

**Dipole Moments (debeye)**					
μ(Int) (x,y,z)	[0.2409, -1.9106, -0.0873]			||μ(Int)||	1.9277
μ(TS) (x,y,z)	[0.4308, 1.2389, 0.721]			||μ(TS)||	1.4968
μ(rxn) (x,y,z)	[0.1899, 3.1495, 0.8083]			||μ(rxn)||	3.2571

**Oriented Electric Field**					
F [× 10^{-3} a.u.]	0	-2.5	-5	-7.5	-10
**Electronic Energies**					
Int [a.u.]	-210.1584	-210.1568	-210.1555	-210.1546	-210.1540
TS [a.u.]	-210.0811	-210.0827	-210.0846	-210.0869	-210.0895
$\Delta E^{‡}$ [kcal/mol]	48.52	46.51	44.50	42.50	40.51
$\Delta\Delta E^{‡}$ [kcal/mol]	0	-2.01	-4.01	-6.01	-8.01
**Free Energies**					
Int [a.u.]	-210.1014	-210.0998	-210.0986	-210.0978	-210.0974
TS [a.u.]	-210.0290	-210.0306	-210.0325	-210.0347	-210.0374
$\Delta G^{‡}$ [kcal/mol]	45.38	43.45	41.52	39.59	37.67
$\Delta\Delta G^{‡}$ [kcal/mol]	0	-1.93	-3.86	-5.79	-7.71
**RMSD from 0-Field**					
Int (Å)	-	0.0021	0.0047	0.0082	0.0121
TS (Å)	-	0.0024	0.0047	0.0070	0.0093

Table S5. Summary of A.V.E.D.A. output data for Reaction 1 computed at the ωB97X-D/def2-TZVP level of theory (gas phase, 298 K).

**Dipole Moments (debeye)**					
μ(Int) (x,y,z)	[0.2889, -1.9325, -0.0901]			||μ(Int)||	1.9561
μ(TS) (x,y,z)	[0.384, 1.2573, 0.7208]			||μ(TS)||	1.4993
μ(rxn) (x,y,z)	[0.0951, 3.1898, 0.8109]			||μ(rxn)||	3.2926

**Oriented Electric Field**					
F [× 10^{-3} a.u.]	0	-2.5	-5	-7.5	-10
**Electronic Energies**					
Int [a.u.]	-210.1767	-210.1750	-210.1737	-210.1728	-210.1722
TS [a.u.]	-210.0985	-210.1001	-210.1020	-210.1043	-210.1069
$\Delta E^{\ddagger}$ [kcal/mol]	49.07	47.04	45.01	42.99	40.97
$\Delta\Delta E^{\ddagger}$ [kcal/mol]	0	-2.03	-4.06	-6.08	-8.10
**Free Energies**					
Int [a.u.]	-210.1197	-210.1181	-210.1170	-210.1162	-210.1158
TS [a.u.]	-210.0465	-210.0480	-210.0499	-210.0522	-210.0548
$\Delta G^{\ddagger}$ [kcal/mol]	45.98	44.03	42.08	40.15	38.25
$\Delta\Delta G^{\ddagger}$ [kcal/mol]	0	-1.95	-3.90	-5.83	-7.73
**RMSD from 0-Field**					
Int (Å)	-	0.0022	0.0047	0.0084	0.0128
TS (Å)	-	0.0024	0.0048	0.0072	0.0096

Table S6. Summary of A.V.E.D.A. output data for Reaction 1 computed in the gas phase at 298 K.

Level of Theory	F = 0 a.u.			F = 2.5 10^{-3} a.u.				F = 5.0 10^{-3} a.u.			
	E_{Int}	E_{TS}	$\Delta E^{\ddagger}$	E_{Int}	E_{TS}	$\Delta E^{\ddagger}$	$\Delta\Delta E^{\ddagger}$	E_{Int}	E_{TS}	$\Delta E^{\ddagger}$	$\Delta\Delta E^{\ddagger}$
B3LYP/def2-TZVP											
Electronic Energy	-210.2534	-210.1757	48.78	-210.2518	-210.1773	46.78	-1.99	-210.2506	-210.1792	44.80	-3.98
Enthalpy	-210.1662	-210.0938	45.44	-210.1647	-210.0954	43.50	-1.94	-210.1635	-210.0972	41.56	-3.88
Free Energy	-210.1976	-210.1248	45.69	-210.1960	-210.1263	43.77	-1.91	-210.1949	-210.1282	41.88	-3.81
B3LYP-D3/def2-TZVP											
Electronic Energy	-210.2570	-210.1791	48.88	-210.2553	-210.1806	46.89	-1.99	-210.2541	-210.1825	44.90	-3.98
Enthalpy	-210.1698	-210.0973	45.51	-210.1682	-210.0988	43.57	-1.94	-210.1670	-210.1007	41.63	-3.88
Free Energy	-210.2011	-210.1282	45.76	-210.1996	-210.1297	43.85	-1.91	-210.1985	-210.1316	41.95	-3.80
M06-2X/def2-TZVP											
Electronic Energy	-210.1584	-210.0811	48.52	-210.1568	-210.0827	46.51	-2.01	-210.1555	-210.0846	44.50	-4.01
Enthalpy	-210.0702	-209.9982	45.19	-210.0686	-209.9997	43.23	-1.96	-210.0674	-210.0016	41.27	-3.92
Free Energy	-210.1014	-210.0290	45.38	-210.0998	-210.0306	43.45	-1.93	-210.0986	-210.0325	41.52	-3.86
ωB97X-D/def2-TZVP											
Electronic Energy	-210.1767	-210.0985	49.07	-210.1750	-210.1001	47.04	-2.03	-210.1737	-210.1020	45.01	-4.06
Enthalpy	-210.0885	-210.0156	45.77	-210.0869	-210.0171	43.78	-1.98	-210.0856	-210.0190	41.80	-3.96
Free Energy	-210.1197	-210.0465	45.98	-210.1181	-210.0480	44.03	-1.95	-210.1170	-210.0499	42.08	-3.90

Table S7. Summary of A.V.E.D.A. output data for Reaction 1 computed in the gas phase at 298 K, continued.

Level of Theory	F = 0 a.u.			F = 7.5 10^{-3} a.u.				F = 10.0 10^{-3} a.u.			
	E_{Int}	E_{TS}	$\Delta E^{\ddagger}$	E_{Int}	E_{TS}	$\Delta E^{\ddagger}$	$\Delta\Delta E^{\ddagger}$	E_{Int}	E_{TS}	$\Delta E^{\ddagger}$	$\Delta\Delta E^{\ddagger}$
B3LYP/def2-TZVP											
Electronic Energy	-210.2534	-210.1757	48.78	-210.2497	-210.1814	42.81	-5.97	-210.2491	-210.1841	40.83	-7.95
Enthalpy	-210.1662	-210.0938	45.44	-210.1627	-210.0995	39.62	-5.82	-210.1622	-210.1022	37.70	-7.74
Free Energy	-210.1976	-210.1248	45.69	-210.1942	-210.1304	40.00	-5.69	-210.1939	-210.1331	38.14	-7.54
B3LYP-D3/def2-TZVP											
Electronic Energy	-210.2570	-210.1791	48.88	-210.2532	-210.1848	42.91	-5.97	-210.2527	-210.1874	40.93	-7.95
Enthalpy	-210.1698	-210.0973	45.51	-210.1662	-210.1030	39.70	-5.81	-210.1658	-210.1056	37.77	-7.74
Free Energy	-210.2011	-210.1282	45.76	-210.1978	-210.1339	40.08	-5.68	-210.1974	-210.1365	38.22	-7.54
M06-2X/def2-TZVP											
Electronic Energy	-210.1584	-210.0811	48.52	-210.1546	-210.0869	42.50	-6.01	-210.1540	-210.0895	40.51	-8.01
Enthalpy	-210.0702	-209.9982	45.19	-210.0665	-210.0039	39.31	-5.88	-210.0660	-210.0065	37.35	-7.84
Free Energy	-210.1014	-210.0290	45.38	-210.0978	-210.0347	39.59	-5.79	-210.0974	-210.0374	37.67	-7.71
ωB97X-D/def2-TZVP											
Electronic Energy	-210.1767	-210.0985	49.07	-210.1728	-210.1043	42.99	-6.08	-210.1722	-210.1069	40.97	-8.10
Enthalpy	-210.0885	-210.0156	45.77	-210.0848	-210.0213	39.83	-5.93	-210.0843	-210.0240	37.87	-7.90
Free Energy	-210.1197	-210.0465	45.98	-210.1162	-210.0522	40.15	-5.83	-210.1158	-210.0548	38.25	-7.73

4.2 Reaction 2

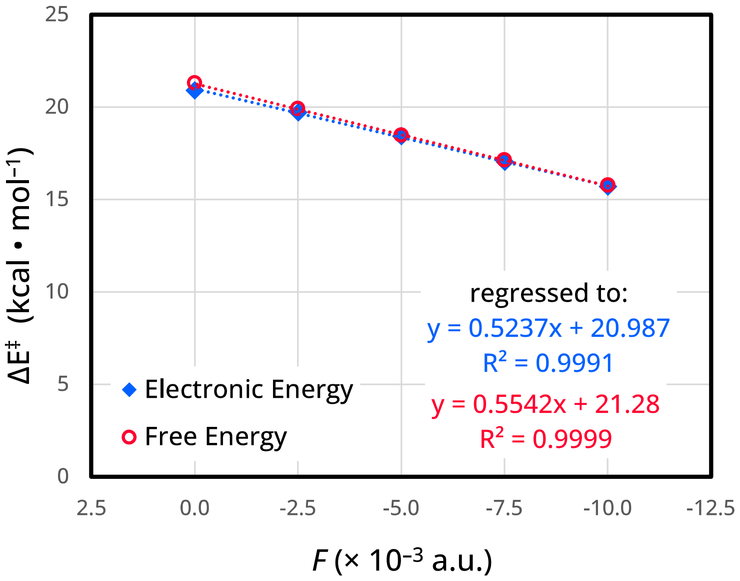

Figure S8. Effective activation energy ($\Delta E^{\ddagger}$ or $\Delta G^{\ddagger}$, kcal/mol) as a function of OEF magnitude for Reaction 2 computed at the B3LYP/def2-TZVP level of theory (gas phase, 298 K).

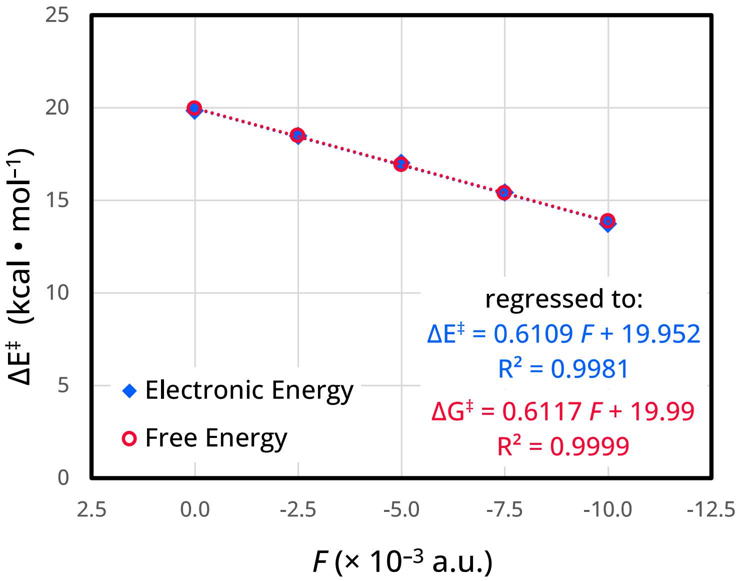

Figure S9. Effective activation energy ($\Delta E^{\ddagger}$ or $\Delta G^{\ddagger}$, kcal/mol) as a function of OEF magnitude for Reaction 2 computed at the B3LYP-D3/def2-TZVP level of theory (gas phase, 298 K).

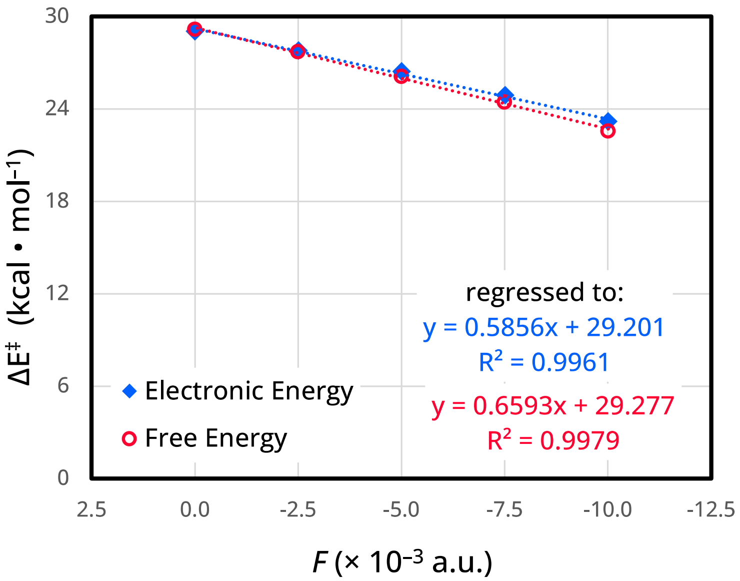

Figure S10. Effective activation energy (Δ*E*‡ or Δ*G*‡, kcal/mol) as a function of OEF magnitude for Reaction 2 computed at the M06-2X/def2-TZVP level of theory (gas phase, 298 K).

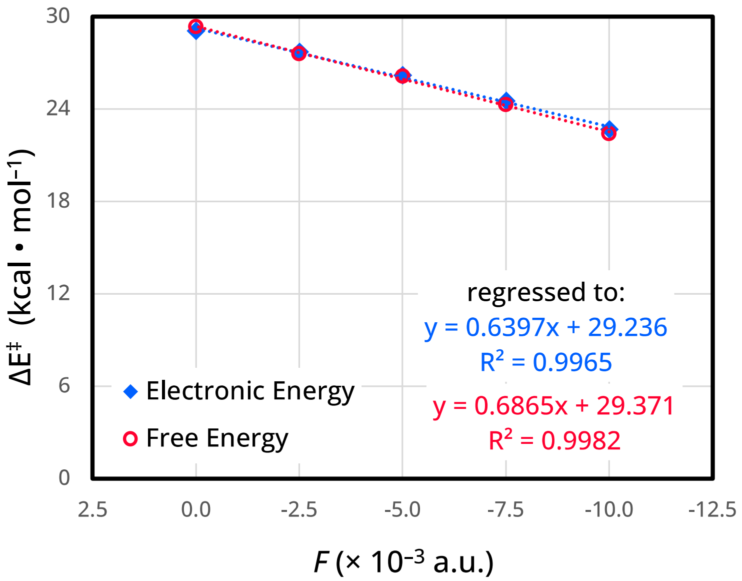

Figure S11. Effective activation energy (Δ*E*‡ or Δ*G*‡, kcal/mol) as a function of OEF magnitude for Reaction 2 computed at the ωB97X-D/def2-TZVP level of theory (gas phase, 298 K).

Table S8. Summary of A.V.E.D.A. output data for Reaction 2 computed at the B3LYP/def2-TZVP level of theory (gas phase, 298 K).

**Dipole Moments (debeye)**					
μ(Int) (x,y,z)	[0.0644, -1.8643, 1.6177]			||μ(Int)||	2.4692
μ(TS) (x,y,z)	[1.5778, -2.8512, 1.3992]			||μ(TS)||	3.5463
μ(rxn) (x,y,z)	[1.5134, -0.9869, -0.2185]			||μ(rxn)||	1.8199
**Oriented Electric Field**					
F [× 10^{-3} a.u.]	0	-2.5	-5	-7.5	-10
**Electronic Energies**					
Int [a.u.]	-669.4711	-669.4724	-669.4747	-669.4780	-669.4826
TS [a.u.]	-669.4378	-669.4410	-669.4453	-669.4509	-669.4576
$\Delta E^{\ddagger}$ [kcal/mol]	20.91	19.73	18.44	17.04	15.71
$\Delta\Delta E^{\ddagger}$ [kcal/mol]	0.00	-1.18	-2.48	-3.87	-5.20
**Free Energies**					
Int [a.u.]	-669.2904	-669.2917	-669.2940	-669.2976	-669.3026
TS [a.u.]	-669.2565	-669.2599	-669.2645	-669.2703	-669.2775
$\Delta G^{\ddagger}$ [kcal/mol]	21.29	19.90	18.48	17.12	15.76
$\Delta\Delta G^{\ddagger}$ [kcal/mol]	0.00	-1.39	-2.82	-4.17	-5.54
**RMSD from 0-Field**					
Int (Å)	-	0.0056	0.0079	0.0091	0.0356
TS (Å)	-	0.0188	0.0549	0.1038	0.1493

Table S9. Summary of A.V.E.D.A. output data for Reaction 2 computed at the B3LYP-D3/def2-TZVP level of theory (gas phase, 298 K).

**Dipole Moments (debeye)**					
μ(Int) (x,y,z)	[–0.1313, –1.6860, 1.8263]			‖μ(Int)‖	2.4890
μ(TS) (x,y,z)	[1.5291, –2.9343, 1.4541]			‖μ(TS)‖	3.6142
μ(rxn) (x,y,z)	[1.6604, -1.2483, -0.3722]			‖μ(rxn)‖	2.1104
**Oriented Electric Field**					
F [× 10^{-3} a.u.]	0	-2.5	-5	-7.5	-10
**Electronic Energies**					
Int [a.u.]	-669.5006	-669.5016	-669.5035	-669.5064	-669.5104
TS [a.u.]	-669.4689	-669.4721	-669.4764	-669.4819	-669.4885
$\Delta E^{\ddagger}$ [kcal/mol]	19.84	18.48	17.01	15.42	13.73
$\Delta\Delta E^{\ddagger}$ [kcal/mol]	0.00	-1.36	-2.83	-4.42	-6.10
**Free Energies**					
Int [a.u.]	-669.3190	-669.3200	-669.3221	-669.3253	-669.3298
TS [a.u.]	-669.2872	-669.2906	-669.2951	-669.3008	-669.3077
$\Delta G^{\ddagger}$ [kcal/mol]	19.97	18.49	16.93	15.38	13.88
$\Delta\Delta G^{\ddagger}$ [kcal/mol]	0.00	-1.48	-3.04	-4.59	-6.09
**RMSD from 0-Field**					
Int (Å)	-	0.0038	0.0074	0.0076	0.0190
TS (Å)	-	0.0195	0.0699	0.1121	0.1613

Table S10. Summary of A.V.E.D.A. output data for Reaction 2 computed at the M06-2X/def2-TZVP level of theory (gas phase, 298 K).

**Dipole Moments (debeye)**					
μ(Int) (x,y,z)	[-0.1348, -1.6172, 1.6697]			‖μ(Int)‖	2.3284
μ(TS) (x,y,z)	[1.4443, -2.6652, 1.3312]			‖μ(TS)‖	3.3108
μ(rxn) (x,y,z)	[1.5791, -1.048, -0.3385]			‖μ(rxn)‖	1.9252

**Oriented Electric Field**					
F [× 10^{-3} a.u.]	0	-2.5	-5	-7.5	-10
**Electronic Energies**					
Int [a.u.]	-669.2025	-669.2034	-669.2051	-669.2078	-669.2113
TS [a.u.]	-669.1562	-669.1591	-669.1630	-669.1681	-669.1743
$\Delta E^{\ddagger}$ [kcal/mol]	29.05	27.80	26.43	24.90	23.19
$\Delta\Delta E^{\ddagger}$ [kcal/mol]	0.00	-1.25	-2.63	-4.16	-5.87
**Free Energies**					
Int [a.u.]	-669.0173	-669.0181	-669.0199	-669.0226	-669.0263
TS [a.u.]	-668.9709	-668.9740	-668.9783	-668.9837	-668.9904
$\Delta G^{\ddagger}$ [kcal/mol]	29.15	27.68	26.10	24.42	22.55
$\Delta\Delta G^{\ddagger}$ [kcal/mol]	0.00	-1.47	-3.05	-4.74	-6.61
**RMSD from 0-Field**					
Int (Å)	-	0.0151	0.0284	0.0395	0.0584
TS (Å)	-	0.0199	0.0418	0.0664	0.0950

Table S11. Summary of A.V.E.D.A. output data for Reaction 2 computed at the ωB97X-D /def2-TZVP level of theory (gas phase, 298 K).

**Dipole Moments (debeye)**					
μ(Int) (x,y,z)	[-0.182, -1.6169, 1.7893]			‖μ(Int)‖	2.4185
μ(TS) (x,y,z)	[1.5589, -2.7801, 1.4374]			‖μ(TS)‖	3.4965
μ(rxn) (x,y,z)	[1.7409, -1.1632, -0.3519]			‖μ(rxn)‖	2.1231
**Oriented Electric Field**					
F [$\times 10^{-3}$ a.u.]	0	-2.5	-5	-7.5	-10
**Electronic Energies**					
Int [a.u.]	-669.2632	-669.2640	-669.2658	-669.2684	-669.2719
TS [a.u.]	-669.2168	-669.2199	-669.2240	-669.2293	-669.2358
$\Delta E^{‡}$ [kcal/mol]	29.08	27.71	26.20	24.53	22.68
$\Delta\Delta E^{‡}$ [kcal/mol]	0.00	-1.37	-2.89	-4.55	-6.41
**Free Energies**					
Int [a.u.]	-669.0788	-669.0796	-669.0814	-669.0843	-669.0881
TS [a.u.]	-669.0321	-669.0356	-669.0398	-669.0456	-669.0524
$\Delta G^{‡}$ [kcal/mol]	29.32	27.59	26.11	24.28	22.39
$\Delta\Delta G^{‡}$ [kcal/mol]	0.00	-1.73	-3.21	-5.04	-6.93
**RMSD from 0-Field**					
Int (Å)	-	0.0093	0.0202	0.0353	0.0505
TS (Å)	-	0.0176	0.0567	0.0895	0.1322

Table S12. Summary of A.V.E.D.A. output data for Reaction 2 computed in the gas phase at 298 K.

Level of Theory	F = 0 a.u.			F = 2.5 10^{-3} a.u.				F = 5.0 10^{-3} a.u.			
	E_{Int}	E_{TS}	$\Delta E^{\ddagger}$	E_{Int}	E_{TS}	$\Delta E^{\ddagger}$	$\Delta\Delta E^{\ddagger}$	E_{Int}	E_{TS}	$\Delta E^{\ddagger}$	$\Delta\Delta E^{\ddagger}$
B3LYP/def2-TZVP											
Electronic Energy	-669.4711	-669.4378	20.91	-669.4724	-669.4410	19.73	-1.18	-669.4747	-669.4454	18.44	-2.48
Enthalpy	-669.2367	-669.2051	19.81	-669.2380	-669.2084	18.58	-1.22	-669.2403	-669.2128	17.25	-2.56
Free Energy	-669.2904	-669.2565	21.29	-669.2917	-669.2600	19.90	-1.39	-669.2940	-669.2645	18.48	-2.82
B3LYP-D3/def2-TZVP											
Electronic Energy	-669.5006	-669.4689	19.84	-669.5016	-669.4721	18.48	-1.36	-669.5035	-669.4764	17.01	-2.83
Enthalpy	-669.2659	-669.2360	18.72	-669.2669	-669.2393	17.33	-1.40	-669.2688	-669.2436	15.82	-2.90
Free Energy	-669.3190	-669.2872	19.97	-669.3200	-669.2906	18.49	-1.48	-669.3221	-669.2951	16.94	-3.04
M06-2X/def2-TZVP											
Electronic Energy	-669.2025	-669.1562	29.05	-669.2034	-669.1591	27.80	-1.25	-669.2051	-669.1630	26.43	-2.63
Enthalpy	-668.9649	-668.9206	27.83	-668.9658	-668.9235	26.53	-1.30	-668.9676	-668.9276	25.10	-2.73
Free Energy	-669.0173	-668.9709	29.16	-669.0181	-668.9740	27.68	-1.47	-669.0199	-668.9783	26.10	-3.05
ωB97X-D/def2-TZVP											
Electronic Energy	-669.2632	-669.2168	29.08	-669.2640	-669.2199	27.71	-1.37	-669.2658	-669.2240	26.20	-2.89
Enthalpy	-669.0259	-668.9813	27.99	-669.0268	-668.9846	26.47	-1.52	-669.0286	-668.9888	24.98	-3.01
Free Energy	-669.0788	-669.0321	29.32	-669.0796	-669.0356	27.59	-1.73	-669.0814	-669.0398	26.11	-3.21

Table S13. Summary of A.V.E.D.A. output data for Reaction 2 computed in the gas phase at 298 K, continued

Level of Theory	F = 0 a.u.			F = 7.5 10^{-3} a.u.				F = 10.0 10^{-3} a.u.			
	E_{Int}	E_{TS}	$\Delta E^{‡}$	E_{Int}	E_{TS}	$\Delta E^{‡}$	$\Delta\Delta E^{‡}$	E_{Int}	E_{TS}	$\Delta E^{‡}$	$\Delta\Delta E^{‡}$
B3LYP/def2-TZVP											
Electronic Energy	-669.4711	-669.4378	20.91	-669.4780	-669.4509	17.04	-3.87	-669.4826	-669.4576	15.71	-5.20
Enthalpy	-669.2367	-669.2051	19.81	-669.2437	-669.2185	15.82	-3.99	-669.2484	-669.2254	14.45	-5.36
Free Energy	-669.2904	-669.2565	21.29	-669.2976	-669.2704	17.12	-4.17	-669.3026	-669.2775	15.76	-5.54
B3LYP-D3/def2-TZVP											
Electronic Energy	-669.5006	-669.4689	19.84	-669.5064	-669.4819	15.42	-4.42	-669.5104	-669.4885	13.73	-6.10
Enthalpy	-669.2659	-669.2360	18.72	-669.2718	-669.2492	14.20	-4.52	-669.2758	-669.2559	12.48	-6.24
Free Energy	-669.3190	-669.2872	19.97	-669.3253	-669.3008	15.38	-4.59	-669.3298	-669.3077	13.88	-6.09
M06-2X/def2-TZVP											
Electronic Energy	-669.2025	-669.1562	29.05	-669.2078	-669.1681	24.90	-4.16	-669.2113	-669.1743	23.19	-5.87
Enthalpy	-668.9649	-668.9206	27.83	-668.9703	-668.9328	23.51	-4.31	-668.9739	-668.9393	21.74	-6.08
Free Energy	-669.0173	-668.9709	29.16	-669.0226	-668.9837	24.42	-4.74	-669.0263	-668.9904	22.55	-6.61
ωB97X-D/def2-TZVP											
Electronic Energy	-669.2632	-669.2168	29.08	-669.2684	-669.2293	24.53	-4.55	-669.2719	-669.2358	22.68	-6.41
Enthalpy	-669.0259	-668.9813	27.99	-669.0313	-668.9942	23.30	-4.69	-669.0350	-669.0009	21.39	-6.60
Free Energy	-669.0788	-669.0321	29.32	-669.0842	-669.0456	24.28	-5.04	-669.0881	-669.0524	22.39	-6.93

4.3 Reaction 3

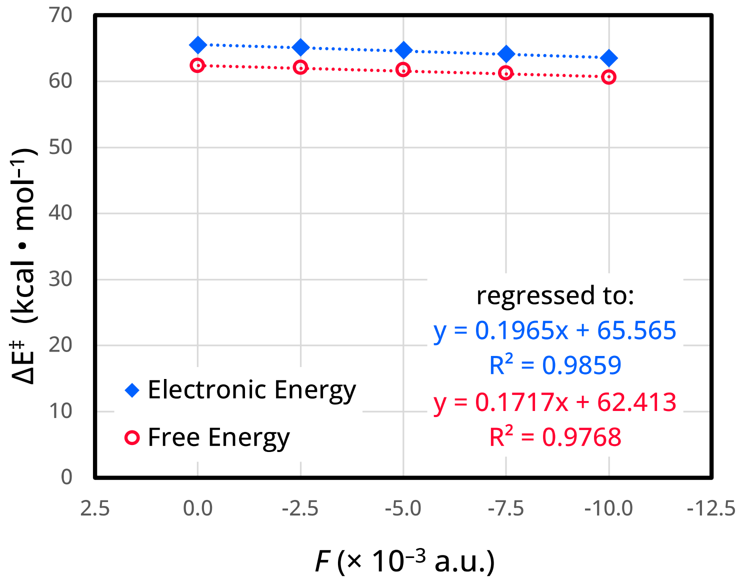

Figure S12. Effective activation energy ($\Delta E^‡$ or $\Delta G^‡$, kcal/mol) as a function of OEF magnitude for Reaction 3 computed at the B3LYP/def2-TZVP level of theory (gas phase, 298 K).

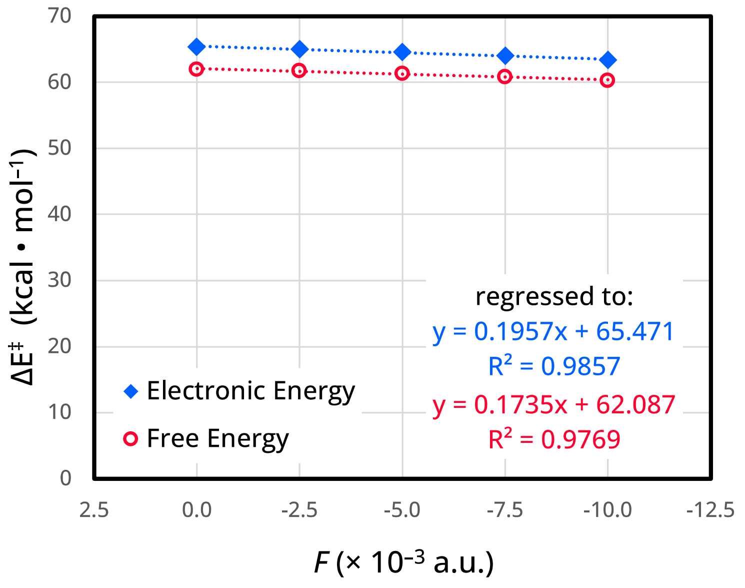

Figure S13. Effective activation energy ($\Delta E^‡$ or $\Delta G^‡$, kcal/mol) as a function of OEF magnitude for Reaction 3 computed at the B3LYP-D3/def2-TZVP level of theory (gas phase, 298 K).

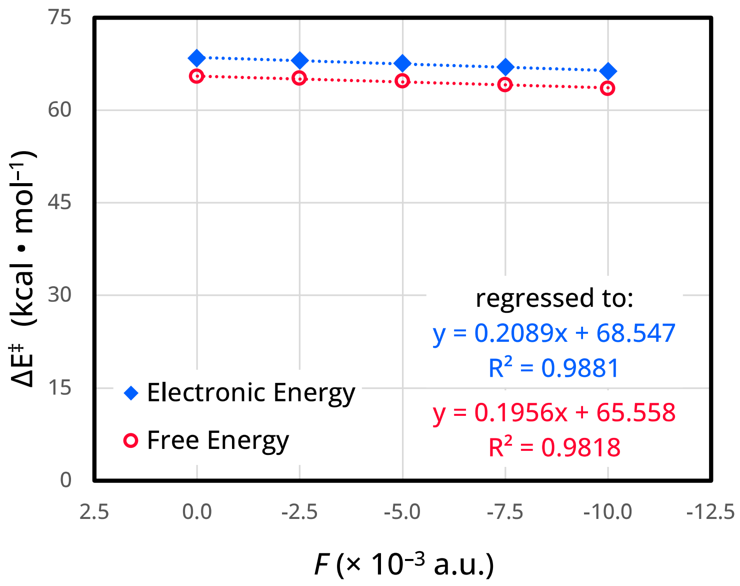

Figure S14. Effective activation energy ($\Delta E^{\ddagger}$ or $\Delta G^{\ddagger}$, kcal/mol) as a function of OEF magnitude for Reaction 3 computed at the M06-2X/def2-TZVP level of theory (gas phase, 298 K).

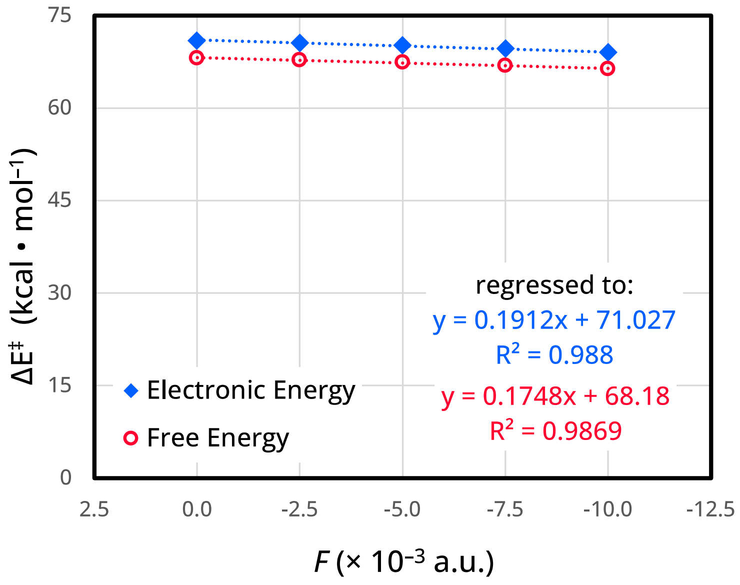

Figure S15. Effective activation energy ($\Delta E^{\ddagger}$ or $\Delta G^{\ddagger}$, kcal/mol) as a function of OEF magnitude for Reaction 3 computed at the ωB97X-D/def2-TZVP level of theory (gas phase, 298 K).

Table S14. Summary of A.V.E.D.A. output data for Reaction 3 computed at the B3LYP/def2-TZVP level of theory (gas phase, 298 K).

**Dipole Moments (debeye)**					
μ(Int) (x,y,z)	[-0.182, -1.6169, 1.7893]			||μ(Int)||	2.4185
μ(TS) (x,y,z)	[1.5589, -2.7801, 1.4374]			||μ(TS)||	3.4965
μ(rxn) (x,y,z)	[1.7409, -1.1632, -0.3519]			||μ(rxn)||	2.1231

**Oriented Electric Field**					
F [× 10^{-3} a.u.]	0	-2.5	-5	-7.5	-10
**Electronic Energies**					
Int [a.u.]	-669.2632	-669.2640	-669.2658	-669.2684	-669.2719
TS [a.u.]	-669.2168	-669.2199	-669.2240	-669.2293	-669.2358
$\Delta E^{\ddagger}$ [kcal/mol]	29.08	27.71	26.20	24.53	22.68
$\Delta\Delta E^{\ddagger}$ [kcal/mol]	0.00	-1.37	-2.89	-4.55	-6.41
**Free Energies**					
Int [a.u.]	-669.0788	-669.0796	-669.0814	-669.0842	-669.0881
TS [a.u.]	-669.0321	-669.0356	-669.0398	-669.0456	-669.0524
$\Delta G^{\ddagger}$ [kcal/mol]	29.32	27.59	26.11	24.28	22.39
$\Delta\Delta G^{\ddagger}$ [kcal/mol]	0.00	-1.73	-3.21	-5.04	-6.93
**RMSD from 0-Field**					
Int (Å)	-	0.0093	0.0202	0.0353	0.0505
TS (Å)	-	0.0176	0.0567	0.0895	0.1322

Table S15. Summary of A.V.E.D.A. output data for Reaction 3 computed at the B3LYP-D3/def2-TZVP level of theory (gas phase, 298 K).

**Dipole Moments (debeye)**					
μ(Int) (x,y,z)	[-1.3707, 0.4511, -0.1829]			‖μ(Int)‖	1.4546
μ(TS) (x,y,z)	[-1.7898, 0.2059, -0.2096]			‖μ(TS)‖	1.8138
μ(rxn) (x,y,z)	[-0.4191, -0.2452, -0.0267]			‖μ(rxn)‖	0.4863

**Oriented Electric Field**					
F [× 10^{-3} a.u.]	0	-2.5	-5	-7.5	-10
**Electronic Energies**					
Int [a.u.]	-387.4075	-387.4087	-387.4106	-387.4130	-387.4161
TS [a.u.]	-387.3033	-387.3051	-387.3076	-387.3110	-387.3150
$\Delta E^‡$ [kcal/mol]	65.37	65.03	64.59	64.06	63.41
$\Delta\Delta E^‡$ [kcal/mol]	0.00	-0.34	-0.78	-1.32	-1.96
**Free Energies**					
Int [a.u.]	-387.2606	-387.2619	-387.2638	-387.2663	-387.2695
TS [a.u.]	-387.1619	-387.1636	-387.1661	-387.1694	-387.1735
$\Delta G^‡$ [kcal/mol]	61.97	61.71	61.33	60.84	60.24
$\Delta\Delta G^‡$ [kcal/mol]	0.00	-0.26	-0.64	-1.14	-1.73
**RMSD from 0-Field**					
Int (Å)	-	0.0025	0.0111	0.0170	0.0208
TS (Å)	-	0.0117	0.0238	0.0363	0.0494

Table S16. Summary of A.V.E.D.A. output data for Reaction 3 computed at the M06-2X/def2-TZVP level of theory (gas phase, 298 K).

**Dipole Moments (debeye)**					
μ(Int) (x,y,z)	[-1.3994, 0.4553, -0.1308]			||μ(Int)||	1.4774
μ(TS) (x,y,z)	[-1.8431, 0.1605, -0.2111]			||μ(TS)||	1.8621
μ(rxn) (x,y,z)	[-0.4437, -0.2948, -0.0803]			||μ(rxn)||	0.5387

**Oriented Electric Field**					
F [$\times 10^{-3}$ a.u.]	0	-2.5	-5	-7.5	-10
**Electronic Energies**					
Int [a.u.]	-387.2101	-387.2113	-387.2131	-387.2155	-387.2185
TS [a.u.]	-387.1010	-387.1028	-387.1054	-387.1087	-387.1128
$\Delta E^{\ddagger}$ [kcal/mol]	68.45	68.07	67.60	67.03	66.36
$\Delta\Delta E^{\ddagger}$ [kcal/mol]	0.00	-0.38	-0.85	-1.42	-2.09
**Free Energies**					
Int [a.u.]	-387.0610	-387.0624	-387.0641	-387.0665	-387.0696
TS [a.u.]	-386.9568	-386.9585	-386.9610	-386.9643	-386.9684
$\Delta G^{\ddagger}$ [kcal/mol]	65.43	65.16	64.69	64.12	63.51
$\Delta\Delta G^{\ddagger}$ [kcal/mol]	0.00	-0.28	-0.74	-1.32	-1.93
**RMSD from 0-Field**					
Int (Å)	-	0.0037	0.0062	0.0107	0.0295
TS (Å)	-	0.0101	0.0201	0.0303	0.0408

Table S17. Summary of A.V.E.D.A. output data for Reaction 3 computed at the ωB97X-D/def2-TZVP level of theory (gas phase, 298 K).

**Dipole Moments (debeye)**					
μ(Int) (x,y,z)	[-1.3471, 0.4244, -0.1657]			‖μ(Int)‖	1.4221
μ(TS) (x,y,z)	[-1.7819, 0.182, -0.2006]			‖μ(TS)‖	1.8024
μ(rxn) (x,y,z)	[-0.4348, -0.2424, -0.0349]			‖μ(rxn)‖	0.4990
**Oriented Electric Field**					
F [× 10^{-3} a.u.]	0	-2.5	-5	-7.5	-10
**Electronic Energies**					
Int [a.u.]	-387.2609	-387.2622	-387.2641	-387.2665	-387.2696
TS [a.u.]	-387.1479	-387.1497	-387.1523	-387.1556	-387.1596
$\Delta E^{\ddagger}$ [kcal/mol]	70.94	70.59	70.16	69.64	69.03
$\Delta\Delta E^{\ddagger}$ [kcal/mol]	0.00	-0.35	-0.78	-1.30	-1.91
**Free Energies**					
Int [a.u.]	-387.1122	-387.1134	-387.1154	-387.1178	-387.1210
TS [a.u.]	-387.0037	-387.0054	-387.0080	-387.0112	-387.0153
$\Delta G^{\ddagger}$ [kcal/mol]	68.10	67.77	67.41	66.90	66.35
$\Delta\Delta G^{\ddagger}$ [kcal/mol]	0.00	-0.33	-0.69	-1.20	-1.75
**RMSD from 0-Field**					
Int (Å)	-	0.0043	0.0106	0.0168	0.0289
TS (Å)	-	0.0112	0.0226	0.0343	0.0461

Table S18. Summary of A.V.E.D.A. output data for Reaction 3 computed in the gas phase at 298 K.

Level of Theory	F = 0 a.u.			F = 2.5 10^{-3} a.u.				F = 5.0 10^{-3} a.u.			
	E_{Int}	E_{TS}	$\Delta E^{\ddagger}$	E_{Int}	E_{TS}	$\Delta E^{\ddagger}$	$\Delta\Delta E^{\ddagger}$	E_{Int}	E_{TS}	$\Delta E^{\ddagger}$	$\Delta\Delta E^{\ddagger}$
B3LYP/def2-TZVP											
Electronic Energy	-387.3914	-387.2871	65.47	-387.3927	-387.2889	65.12	-0.35	-387.3946	-387.2915	64.68	-0.78
Enthalpy	-387.1993	-387.1015	61.38	-387.2006	-387.1032	61.04	-0.35	-387.2024	-387.1059	60.59	-0.79
Free Energy	-387.2449	-387.1456	62.30	-387.2462	-387.1474	62.04	-0.26	-387.2481	-387.1499	61.67	-0.63
B3LYP-D3/def2-TZVP											
Electronic Energy	-387.4075	-387.3033	65.37	-387.4087	-387.3051	65.03	-0.34	-387.4106	-387.3076	64.59	-0.78
Enthalpy	-387.2152	-387.1177	61.16	-387.2164	-387.1298	54.39	-6.77	-387.2183	-387.1221	60.38	-0.78
Free Energy	-387.2606	-387.1619	61.97	-387.2619	-387.1636	61.71	-0.26	-387.2638	-387.1661	61.34	-0.64
M06-2X/def2-TZVP											
Electronic Energy	-387.2101	-387.1010	68.45	-387.2113	-387.1028	68.07	-0.38	-387.2131	-387.1054	67.60	-0.85
Enthalpy	-387.0159	-386.9131	64.52	-387.0172	-386.9149	64.17	-0.35	-387.0190	-386.9175	63.69	-0.83
Free Energy	-387.0610	-386.9568	65.43	-387.0624	-386.9585	65.16	-0.28	-387.0641	-386.9610	64.69	-0.74
ωB97X-D/def2-TZVP											
Electronic Energy	-387.2609	-387.1479	70.94	-387.2622	-387.1497	70.59	-0.35	-387.2641	-387.1523	70.16	-0.78
Enthalpy	-387.0668	-386.9600	67.05	-387.0681	-386.9618	66.69	-0.36	-387.0700	-386.9644	66.27	-0.78
Free Energy	-387.1122	-387.0037	68.10	-387.1134	-387.0054	67.77	-0.33	-387.1154	-387.0080	67.41	-0.69

Table S19. Summary of A.V.E.D.A. output data for Reaction 3 computed in the gas phase at 298 K, continued.

Level of Theory	F = 0 a.u.			F = 7.5 10^{-3} a.u.				F = 10.0 10^{-3} a.u.			
	E_{Int}	E_{TS}	$\Delta E^{\ddagger}$	E_{Int}	E_{TS}	$\Delta E^{\ddagger}$	$\Delta\Delta E^{\ddagger}$	E_{Int}	E_{TS}	$\Delta E^{\ddagger}$	$\Delta\Delta E^{\ddagger}$
B3LYP/def2-TZVP											
Electronic Energy	-387.3914	-387.2871	65.47	-387.3971	-387.2948	64.15	-1.32	-387.4008	-387.2987	64.08	-1.39
Enthalpy	-387.1993	-387.1015	61.38	-387.2049	-387.1093	60.04	-1.35	-387.2087	-387.1133	59.85	-1.54
Free Energy	-387.2449	-387.1456	62.30	-387.2507	-387.1532	61.17	-1.13	-387.2542	-387.1574	60.76	-1.54
B3LYP-D3/def2-TZVP											
Electronic Energy	-387.4075	-387.3033	65.37	-387.4130	-387.3110	64.06	-1.32	-387.4161	-387.3150	63.41	-1.96
Enthalpy	-387.2152	-387.1177	61.16	-387.2208	-387.1255	59.83	-1.34	-387.2239	-387.1297	59.16	-2.00
Free Energy	-387.2606	-387.1619	61.97	-387.2663	-387.1694	60.84	-1.14	-387.2695	-387.1735	60.24	-1.73
M06-2X/def2-TZVP											
Electronic Energy	-387.2101	-387.1010	68.45	-387.2155	-387.1087	67.03	-1.42	-387.2185	-387.1128	66.36	-2.09
Enthalpy	-387.0159	-386.9131	64.52	-387.0214	-386.9208	63.10	-1.41	-387.0244	-386.9250	62.42	-2.10
Free Energy	-387.0610	-386.9568	65.43	-387.0665	-386.9643	64.12	-1.32	-387.0696	-386.9684	63.51	-1.93
ωB97X-D/def2-TZVP											
Electronic Energy	-387.2609	-387.1479	70.94	-387.2665	-387.1556	69.64	-1.30	-387.2696	-387.1596	69.03	-1.91
Enthalpy	-387.0668	-386.9600	67.05	-387.0725	-386.9677	65.72	-1.33	-387.0756	-386.9718	65.10	-1.95
Free Energy	-387.1122	-387.0037	68.10	-387.1178	-387.0112	66.90	-1.20	-387.1210	-387.0153	66.35	-1.75

4.4 Reaction 4

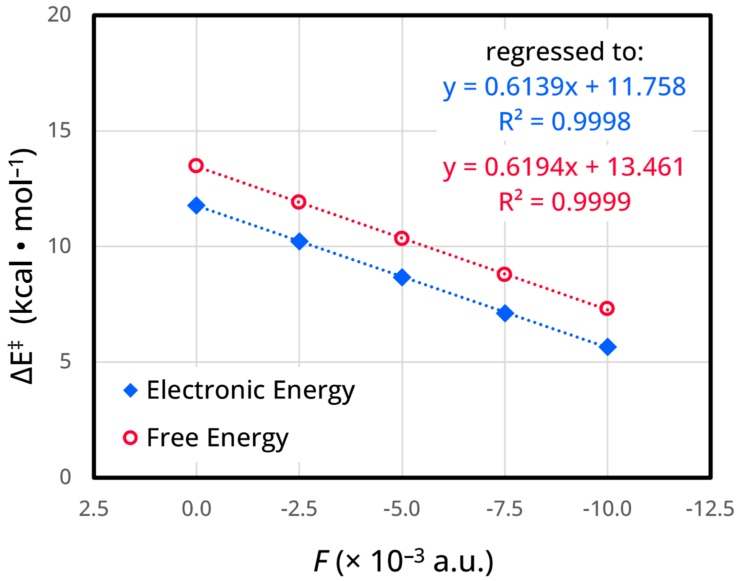

Figure S16. Effective activation energy ($\Delta E^{\ddagger}$ or $\Delta G^{\ddagger}$, kcal/mol) as a function of OEF magnitude for Reaction 4 computed at the B3LYP/def2-TZVP level of theory (gas phase, 298 K).

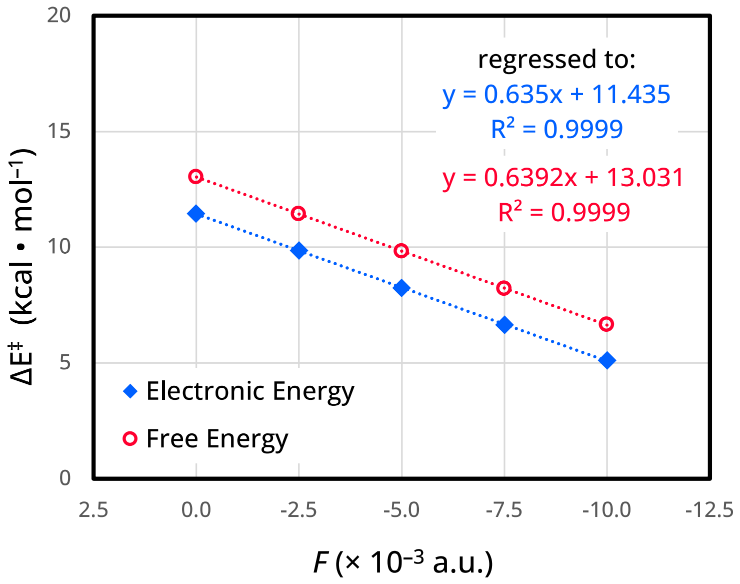

Figure S17. Effective activation energy ($\Delta E^{\ddagger}$ or $\Delta G^{\ddagger}$, kcal/mol) as a function of OEF magnitude for Reaction 4 computed at the B3LYP-D3/def2-TZVP level of theory (gas phase, 298 K).

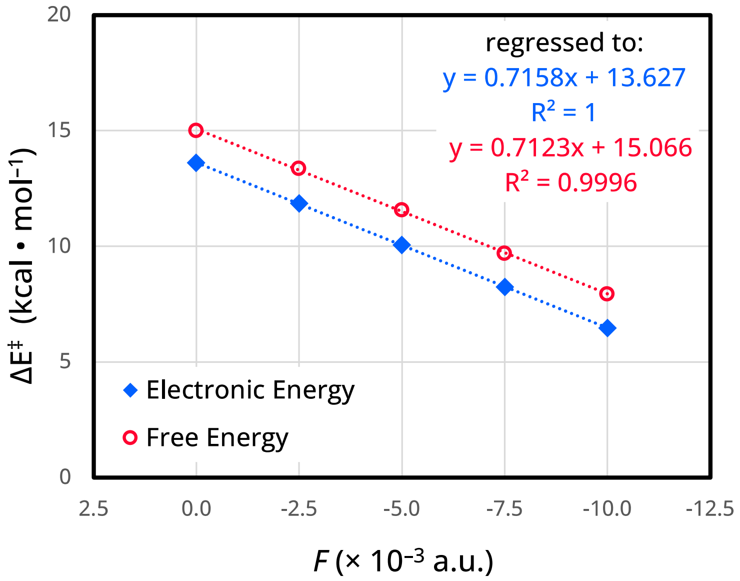

Figure S18. Effective activation energy ($\Delta E^{‡}$ or $\Delta G^{‡}$, kcal/mol) as a function of OEF magnitude for Reaction 4 computed at the M06-2X/def2-TZVP level of theory (gas phase, 298 K).

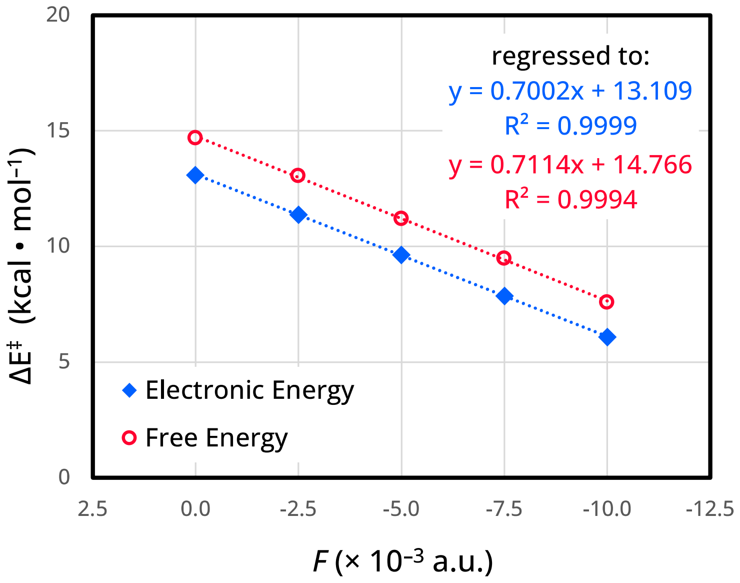

Figure S19. Effective activation energy ($\Delta E^{‡}$ or $\Delta G^{‡}$, kcal/mol) as a function of OEF magnitude for Reaction 4 computed at the ωB97X-D/def2-TZVP level of theory (gas phase, 298 K).

Table S20. Summary of A.V.E.D.A. output data for Reaction 4 computed at the B3LYP/def2-TZVP level of theory (gas phase, 298 K).

**Dipole Moments (debeye)**					
μ(Int) (x,y,z)	[0.4784, 2.2828, -0.5163]			||μ(Int)||	2.3889
μ(TS) (x,y,z)	[2.9086, 2.8419, -0.6207]			||μ(TS)||	4.1136
μ(rxn) (x,y,z)	[2.4302, 0.5591, -0.1044]			||μ(rxn)||	2.4959

**Oriented Electric Field**					
F [× 10^{-3} a.u.]	0	-2.5	-5	-7.5	-10
**Electronic Energies**					
Int [a.u.]	-439.1539	-439.1553	-439.1575	-439.1607	-439.1648
TS [a.u.]	-439.1351	-439.1390	-439.1437	-439.1493	-439.1558
$\Delta E^{\ddagger}$ [kcal/mol]	11.78	10.23	8.66	7.12	5.66
$\Delta\Delta E^{\ddagger}$ [kcal/mol]	0	-1.55	-3.12	-4.66	-6.12
**Free Energies**					
Int [a.u.]	-439.0590	-439.0603	-439.0626	-439.0658	-439.0699
TS [a.u.]	-439.0375	-439.0414	-439.0461	-439.0518	-439.0583
$\Delta G^{\ddagger}$ [kcal/mol]	13.48	11.91	10.33	8.79	7.3035
$\Delta\Delta G^{\ddagger}$ [kcal/mol]	0	-1.57	-3.15	-4.70	-6.18
**RMSD from 0-Field**					
Int (Å)	-	0.0228	0.0393	0.0479	0.0486
TS (Å)	-	0.0192	0.0567	0.0652	0.0915

Table S21. Summary of A.V.E.D.A. output data for Reaction 4 computed at the B3LYP-D3/def2-TZVP level of theory (gas phase, 298 K).

**Dipole Moments (debeye)**					
μ(Int) (x,y,z)	[0.42, 2.2306, -0.5578]			‖μ(Int)‖	2.3373
μ(TS) (x,y,z)	[2.9099, 2.8466, -0.6364]			‖μ(TS)‖	4.1202
μ(rxn) (x,y,z)	[2.4899, 0.6160, -0.0786]			‖μ(rxn)‖	2.5662

**Oriented Electric Field**					
F [× 10^{-3} a.u.]	0	-2.5	-5	-7.5	-10
**Electronic Energies**					
Int [a.u.]	-439.1657	-439.1670	-439.1692	-439.1723	-439.1764
TS [a.u.]	-439.1474	-439.1513	-439.1561	-439.1617	-439.1682
$\Delta E^{\ddagger}$ [kcal/mol]	11.45	9.85	8.24	6.64	5.12
$\Delta\Delta E^{\ddagger}$ [kcal/mol]	0	-1.60	-3.22	-4.81	-6.33
**Free Energies**					
Int [a.u.]	-439.0706	-439.0719	-439.0741	-439.0772	-439.0813
TS [a.u.]	-439.0498	-439.0537	-439.0585	-439.0642	-439.0707
$\Delta G^{\ddagger}$ [kcal/mol]	13.04	11.44	9.82	8.21	6.66
$\Delta\Delta G^{\ddagger}$ [kcal/mol]	0	-1.59	-3.22	-4.83	-6.37
**RMSD from 0-Field**					
Int (Å)	-	0.0124	0.0357	0.0519	0.0381
TS (Å)	-	0.0194	0.0415	0.0649	0.0912

Table S22. Summary of A.V.E.D.A. output data for Reaction 4 computed at the M06-2X/def2-TZVP level of theory (gas phase, 298 K).

**Dipole Moments (debeye)**					
μ(Int) (x,y,z)	[0.2195, 2.1643, -0.6313]			‖μ(Int)‖	2.2652
μ(TS) (x,y,z)	[2.9029, 2.9371, -0.6454]			‖μ(TS)‖	4.1797
μ(rxn) (x,y,z)	[2.6834, 0.7728, -0.0141]			‖μ(rxn)‖	2.7925

**Oriented Electric Field**					
F [$\times 10^{-3}$ a.u.]	0	-2.5	-5	-7.5	-10
**Electronic Energies**					
Int [a.u.]	-438.9681	-438.9693	-438.9712	-438.9740	-438.9775
TS [a.u.]	-438.9464	-438.9504	-438.9552	-438.9608	-438.9672
$\Delta E^{\ddagger}$ [kcal/mol]	13.61	11.86	10.06	8.25	6.47
$\Delta\Delta E^{\ddagger}$ [kcal/mol]	0	-1.75	-3.55	-5.36	-7.14
**Free Energies**					
Int [a.u.]	-438.8711	-438.8724	-438.8743	-438.8770	-438.8806
TS [a.u.]	-438.8472	-438.8511	-438.8559	-438.8615	-438.8680
$\Delta G^{\ddagger}$ [kcal/mol]	15.00	13.35	11.55	9.69	7.92
$\Delta\Delta G^{\ddagger}$ [kcal/mol]	0	-1.65	-3.45	-5.31	-7.08
**RMSD from 0-Field**					
Int (Å)	-	0.0122	0.0353	0.0502	0.0647
TS (Å)	-	0.0167	0.0358	0.0563	0.0800

Table S23. Summary of A.V.E.D.A. output data for Reaction 4 computed at the ωB97X-D /def2-TZVP level of theory (gas phase, 298 K).

**Dipole Moments (debeye)**					
μ(Int) (x,y,z)	[0.1706, 2.1642, -0.7482]			‖μ(Int)‖	2.2962
μ(TS) (x,y,z)	[2.8022, 2.892, -0.6811]			‖μ(TS)‖	4.0841
μ(rxn) (x,y,z)	[2.6316, 0.7278, 0.06710]			‖μ(rxn)‖	2.7312
**Oriented Electric Field**					
F [× 10^{-3} a.u.]	0	-2.5	-5	-7.5	-10
**Electronic Energies**					
Int [a.u.]	-438.9948	-438.9959	-438.9977	-439.0004	-439.0039
TS [a.u.]	-438.9739	-438.9778	-438.9824	-438.9879	-438.9942
$\Delta E^{\ddagger}$ [kcal/mol]	13.08	11.38	9.63	7.86	6.09
$\Delta\Delta E^{\ddagger}$ [kcal/mol]	0	-1.70	-3.45	-5.22	-6.99
**Free Energies**					
Int [a.u.]	-438.8981	-438.8993	-438.9010	-438.9038	-438.9070
TS [a.u.]	-438.8747	-438.8785	-438.8831	-438.8886	-438.8950
$\Delta G^{\ddagger}$ [kcal/mol]	14.69	13.06	11.21	9.49	7.59
$\Delta\Delta G^{\ddagger}$ [kcal/mol]	0	-1.63	-3.48	-5.20	-7.11
**RMSD from 0-Field**					
Int (Å)	-	0.0095	0.0256	0.0349	0.0483
TS (Å)	-	0.0189	0.0386	0.0613	0.0855

Table S24. Summary of A.V.E.D.A. output data for Reaction 4 computed in the gas phase at 298 K.

Level of Theory	F = 0 a.u.			F = 2.5 10^{-3} a.u.				F = 5.0 10^{-3} a.u.			
	E_{Int}	E_{TS}	$\Delta E^{\ddagger}$	E_{Int}	E_{TS}	$\Delta E^{\ddagger}$	$\Delta\Delta E^{\ddagger}$	E_{Int}	E_{TS}	$\Delta E^{\ddagger}$	$\Delta\Delta E^{\ddagger}$
B3LYP/def2-TZVP											
Electronic Energy	-439.1539	-439.1351	11.78	-439.1553	-439.1390	10.23	-1.55	-439.1575	-439.1437	8.66	-3.12
Enthalpy	-439.0139	-438.9966	10.83	-439.0153	-439.0005	9.30	-1.53	-439.0175	-439.0052	7.76	-3.07
Free Energy	-439.0590	-439.0375	13.48	-439.0603	-439.0414	11.91	-1.57	-439.0626	-439.0461	10.34	-3.15
B3LYP-D3/def2-TZVP											
Electronic Energy	-439.1657	-439.1474	11.45	-439.1670	-439.1513	9.85	-1.60	-439.1692	-439.1561	8.24	-3.22
Enthalpy	-439.0257	-439.0090	10.48	-439.0270	-439.0128	8.91	-1.58	-439.0292	-439.0176	7.32	-3.16
Free Energy	-439.0706	-439.0498	13.04	-439.0719	-439.0537	11.45	-1.59	-439.0741	-439.0585	9.82	-3.22
M06-2X/def2-TZVP											
Electronic Energy	-438.9681	-438.9464	13.61	-438.9693	-438.9504	11.86	-1.75	-438.9713	-438.9552	10.06	-3.55
Enthalpy	-438.8265	-438.8065	12.53	-438.8277	-438.8104	10.83	-1.70	-438.8296	-438.8152	9.07	-3.46
Free Energy	-438.8711	-438.8472	15.00	-438.8724	-438.8511	13.35	-1.65	-438.8743	-438.8559	11.55	-3.45
ωB97X-D/def2-TZVP											
Electronic Energy	-438.9948	-438.9739	13.08	-438.9959	-438.9778	11.38	-1.70	-438.9978	-438.9824	9.63	-3.45
Enthalpy	-438.8532	-438.8339	12.09	-438.8543	-438.8377	10.43	-1.66	-438.8561	-438.8423	8.67	-3.42
Free Energy	-438.8981	-438.8747	14.69	-438.8993	-438.8785	13.06	-1.63	-438.9010	-438.8831	11.21	-3.48

Table S25. Summary of A.V.E.D.A. output data for Reaction 4 computed in the gas phase at 298 K, continued.

Level of Theory	F = 0 a.u.			F = 7.5 10^{-3} a.u.				F = 10.0 10^{-3} a.u.			
	E_{Int}	E_{TS}	$\Delta E^{‡}$	E_{Int}	E_{TS}	$\Delta E^{‡}$	$\Delta\Delta E^{‡}$	E_{Int}	E_{TS}	$\Delta E^{‡}$	$\Delta\Delta E^{‡}$
B3LYP/def2-TZVP											
Electronic Energy	-439.1539	-439.1351	11.78	-439.1607	-439.1493	7.12	-4.66	-439.1648	-439.1558	5.66	-6.12
Enthalpy	-439.0139	-438.9966	10.83	-439.0207	-439.0108	6.26	-4.57	-439.0250	-439.0173	4.84	-5.99
Free Energy	-439.0590	-439.0375	13.48	-439.0658	-439.0518	8.79	-4.70	-439.0699	-439.0583	7.30	-6.18
B3LYP-D3/def2-TZVP											
Electronic Energy	-439.1657	-439.1474	11.45	-439.1723	-439.1617	6.64	-4.81	-439.1764	-439.1682	5.12	-6.33
Enthalpy	-439.0257	-439.0090	10.48	-439.0324	-439.0232	5.77	-4.72	-439.0365	-439.0297	4.29	-6.19
Free Energy	-439.0706	-439.0498	13.04	-439.0772	-439.0642	8.21	-4.83	-439.0813	-439.0707	6.66	-6.37
M06-2X/def2-TZVP											
Electronic Energy	-438.9681	-438.9464	13.61	-438.9740	-438.9608	8.25	-5.36	-438.9775	-438.9672	6.47	-7.14
Enthalpy	-438.8265	-438.8065	12.53	-438.8324	-438.8208	7.29	-5.24	-438.8361	-438.8271	5.59	-6.94
Free Energy	-438.8711	-438.8472	15.00	-438.8770	-438.8615	9.70	-5.31	-438.8806	-438.8680	7.92	-7.08
ωB97X-D/def2-TZVP											
Electronic Energy	-438.9948	-438.9739	13.08	-439.0004	-438.9879	7.86	-5.22	-439.0039	-438.9942	6.09	-6.99
Enthalpy	-438.8532	-438.8339	12.09	-438.8589	-438.8478	6.99	-5.10	-438.8624	-438.8540	5.25	-6.83
Free Energy	-438.8981	-438.8747	14.69	-438.9038	-438.8886	9.49	-5.20	-438.9070	-438.8949	7.59	-7.11

4.5 Reaction 5

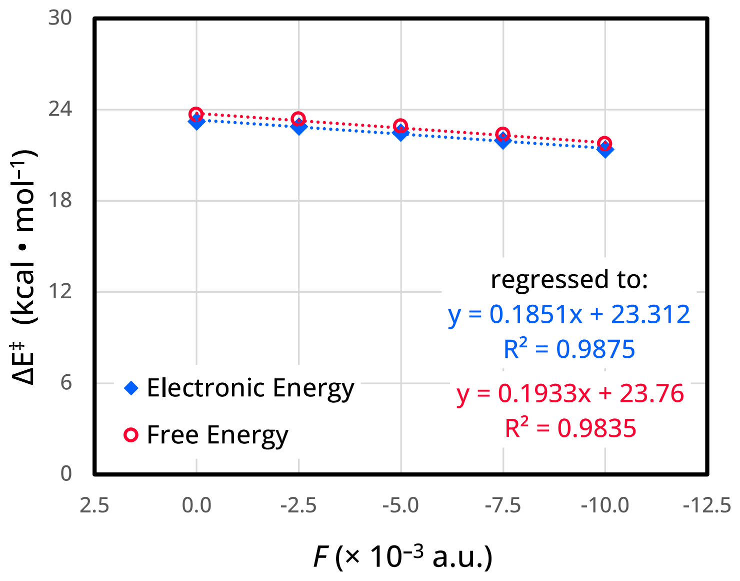

Figure S20. Effective activation energy ($\Delta E^{\ddagger}$ or $\Delta G^{\ddagger}$, kcal/mol) as a function of OEF magnitude for Reaction 5 computed at the B3LYP/def2-TZVP level of theory (gas phase, 298 K).

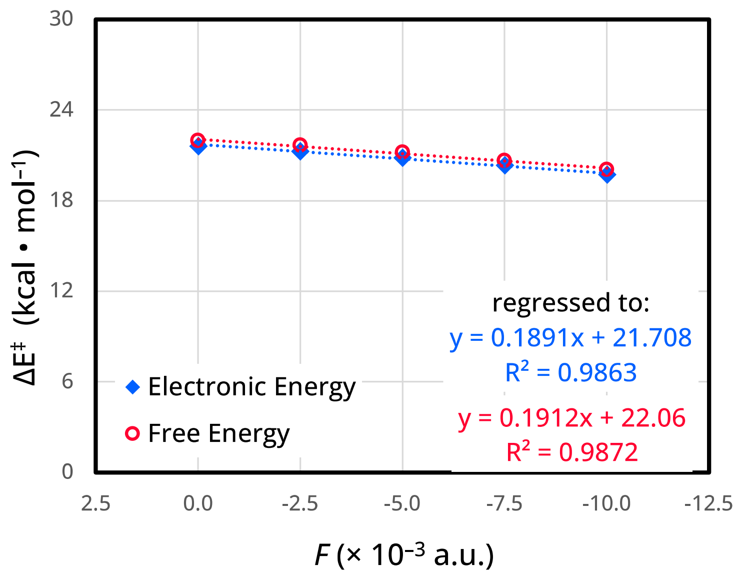

Figure S21. Effective activation energy ($\Delta E^{\ddagger}$ or $\Delta G^{\ddagger}$, kcal/mol) as a function of OEF magnitude for Reaction 5 computed at the B3LYP-D3/def2-TZVP level of theory (gas phase, 298 K).

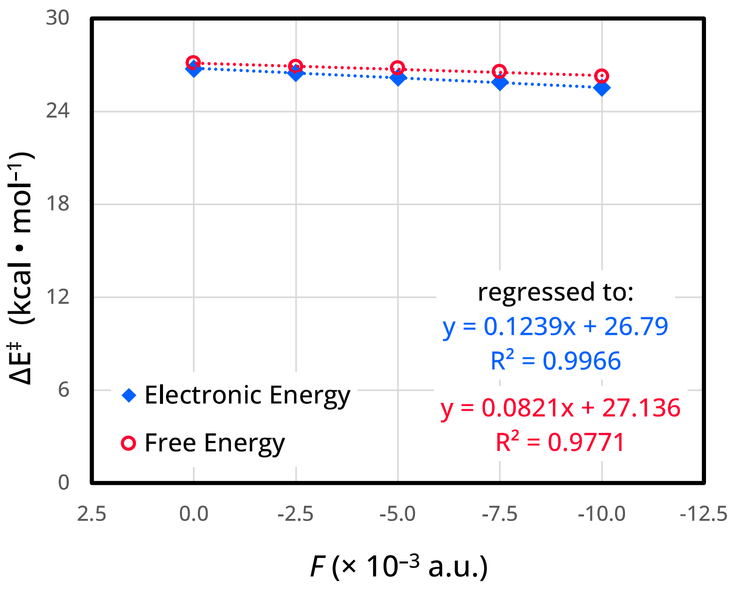

Figure S22. Effective activation energy ($\Delta E^{\ddagger}$ or $\Delta G^{\ddagger}$, kcal/mol) as a function of OEF magnitude for Reaction 5 computed at the M06-2X/def2-TZVP level of theory (gas phase, 298 K).

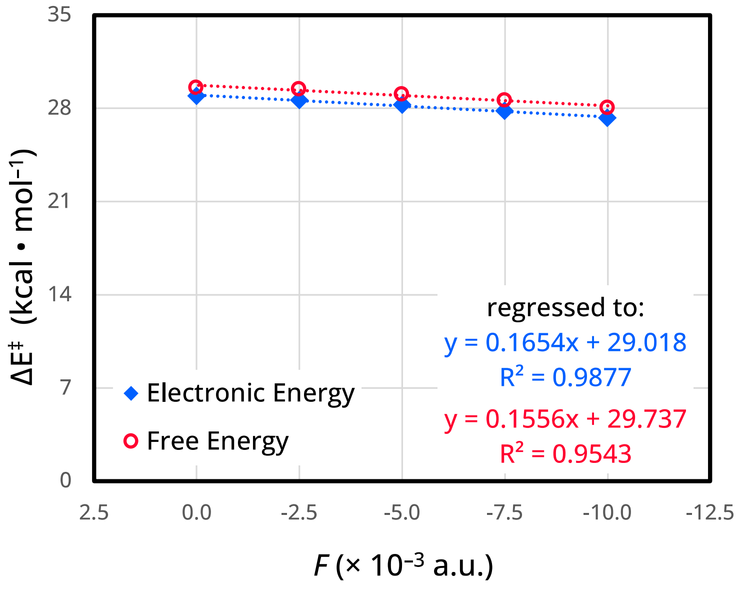

Figure S23. Effective activation energy ($\Delta E^{\ddagger}$ or $\Delta G^{\ddagger}$, kcal/mol) as a function of OEF magnitude for Reaction 5 computed at the ωB97X-D/def2-TZVP level of theory (gas phase, 298 K).

Table S26. Summary of A.V.E.D.A. output data for Reaction 5 computed at the B3LYP/def2-TZVP level of theory (gas phase, 298 K).

**Dipole Moments (debeye)**					
μ(Int) (x,y,z)	[0.4039, -0.0914, -0.3558]			||μ(Int)||	0.5460
μ(TS) (x,y,z)	[0.4405, -0.0, 0.1124]			||μ(TS)||	0.4546
μ(rxn) (x,y,z)	[0.0366, 0.0914, 0.4682]			||μ(rxn)||	0.4784

**Oriented Electric Field**					
F [× 10^{-3} a.u.]	0	-2.5	-5	-7.5	-10
**Electronic Energies**					
Int [a.u.]	-312.1168	-312.1168	-312.1173	-312.1185	-312.1203
TS [a.u.]	-312.0798	-312.0803	-312.0815	-312.0835	-312.0862
ΔE‡ [kcal/mol]	23.23	22.89	22.47	21.97	21.37
ΔΔE‡ [kcal/mol]	0.00	-0.33	-0.75	-1.25	-1.85
**Free Energies**					
Int [a.u.]	-311.9735	-311.9735	-311.9741	-311.9753	-311.9772
TS [a.u.]	-311.9358	-311.9363	-311.9376	-311.9397	-311.9426
ΔG‡ [kcal/mol]	23.65	23.34	22.90	22.35	21.73
ΔΔG‡ [kcal/mol]	0.00	-0.30	-0.75	-1.30	-1.92
**RMSD from 0-Field**					
Int (Å)	-	0.0052	0.0099	0.0140	0.0176
TS (Å)	-	0.0088	0.0201	0.0385	0.0704

Table S27. Summary of A.V.E.D.A. output data for Reaction 5 computed at the B3LYP-D3/def2-TZVP level of theory (gas phase, 298 K).

**Dipole Moments (debeye)**					
μ(Int) (x,y,z)	[0.3951, -0.0987, -0.3456]			‖μ(Int)‖	0.5341
μ(TS) (x,y,z)	[0.4431, -0.0, 0.1192]			‖μ(TS)‖	0.4589
μ(rxn) (x,y,z)	[0.0480, 0.0987, 0.4648]			‖μ(rxn)‖	0.4776

**Oriented Electric Field**					
F [× 10^{-3} a.u.]	0	-2.5	-5	-7.5	-10
**Electronic Energies**					
Int [a.u.]	-312.1321	-312.1321	-312.1327	-312.1339	-312.1356
TS [a.u.]	-312.0977	-312.0982	-312.0995	-312.1014	-312.1042
$\Delta E^‡$ [kcal/mol]	21.62	21.28	20.86	20.34	19.72
$\Delta\Delta E^‡$ [kcal/mol]	0.00	-0.34	-0.76	-1.28	-1.89
**Free Energies**					
Int [a.u.]	-311.9886	-311.9886	-311.9892	-311.9905	-311.9923
TS [a.u.]	-311.9536	-311.9541	-311.9555	-311.9576	-311.9604
$\Delta G^‡$ [kcal/mol]	21.96	21.65	21.19	20.65	20.07
$\Delta\Delta G^‡$ [kcal/mol]	0.00	-0.31	-0.77	-1.31	-1.89
**RMSD from 0-Field**					
Int (Å)	-	0.0049	0.0093	0.0128	0.0160
TS (Å)	-	0.0115	0.0291	0.0718	0.0938

Table S28. Summary of A.V.E.D.A. output data for Reaction 5 computed at the M06-2X/def2-TZVP level of theory (gas phase, 298 K).

**Dipole Moments (debeye)**					
μ(Int) (x,y,z)	[-0.3704, 0.0674, -0.2994]			‖μ(Int)‖	0.4810
μ(TS) (x,y,z)	[-0.4175, -0.0487, 0.076]			‖μ(TS)‖	0.4271
μ(rxn) (x,y,z)	[-0.04710, -0.1161, 0.3754]			‖μ(rxn)‖	0.3958

**Oriented Electric Field**					
F [× 10^{-3} a.u.]	0	-2.5	-5	-7.5	-10
**Electronic Energies**					
Int [a.u.]	-311.9601	-311.9601	-311.9608	-311.9621	-311.9639
TS [a.u.]	-311.9175	-311.9179	-311.9190	-311.9208	-311.9233
$\Delta E^{\ddagger}$ [kcal/mol]	26.76	26.50	26.20	25.88	25.52
$\Delta\Delta E^{\ddagger}$ [kcal/mol]	0.00	-0.26	-0.56	-0.88	-1.24
**Free Energies**					
Int [a.u.]	-311.8146	-311.8146	-311.8153	-311.8168	-311.8188
TS [a.u.]	-311.7713	-311.7718	-311.7727	-311.7744	-311.7770
$\Delta G^{\ddagger}$ [kcal/mol]	27.12	26.89	26.78	26.57	26.26
$\Delta\Delta G^{\ddagger}$ [kcal/mol]	0.00	-0.23	-0.34	-0.56	-0.86
**RMSD from 0-Field**					
Int (Å)	-	0.0039	0.0095	0.0126	0.0157
TS (Å)	-	0.0265	0.0403	0.0501	0.0611

Table S29. Summary of A.V.E.D.A. output data for Reaction 5 computed at the ωB97X-D/def2-TZVP level of theory (gas phase, 298 K).

**Dipole Moments (debeye)**					
μ(Int) (x,y,z)	[0.3907, -0.1075, -0.3418]			$\|\|\mu(Int)\|\|$	0.5301
μ(TS) (x,y,z)	[0.4039, -0.0, 0.0703]			$\|\|\mu(TS)\|\|$	0.4100
μ(rxn) (x,y,z)	[0.0132, 0.1075, 0.4121]			$\|\|\mu(rxn)\|\|$	0.4261

**Oriented Electric Field**					
F [× 10^{-3} a.u.]	0	-2.5	-5	-7.5	-10
**Electronic Energies**					
Int [a.u.]	-312.0117	-312.0117	-312.0122	-312.0133	-312.0150
TS [a.u.]	-311.9656	-311.9660	-311.9671	-311.9690	-311.9715
$\Delta E^‡$ [kcal/mol]	28.94	28.64	28.27	27.82	27.29
$\Delta\Delta E^‡$ [kcal/mol]	0.00	-0.30	-0.67	-1.12	-1.66
**Free Energies**					
Int [a.u.]	-311.8665	-311.8665	-311.8671	-311.8682	-311.8700
TS [a.u.]	-311.8193	-311.8195	-311.8207	-311.8226	-311.8253
$\Delta G^‡$ [kcal/mol]	29.58	29.46	29.09	28.61	28.06
$\Delta\Delta G^‡$ [kcal/mol]	0.00	-0.11	-0.49	-0.96	-1.52
**RMSD from 0-Field**					
Int (Å)	-	0.0041	0.0080	0.0114	0.0144
TS (Å)	-	0.0096	0.0271	0.0512	0.0814

Table S30. Summary of A.V.E.D.A. output data for Reaction 5 computed in the gas phase at 298 K.

Level of Theory	F = 0 a.u.			F = 2.5 10^{-3} a.u.				F = 5.0 10^{-3} a.u.			
	E_{Int}	E_{TS}	$\Delta E^{‡}$	E_{Int}	E_{TS}	$\Delta E^{‡}$	$\Delta\Delta E^{‡}$	E_{Int}	E_{TS}	$\Delta E^{‡}$	$\Delta\Delta E^{‡}$
B3LYP/def2-TZVP											
Electronic Energy	-312.1168	-312.0798	23.23	-312.1168	-312.0803	22.89	-0.34	-312.1173	-312.0815	22.47	-0.75
Enthalpy	-311.9308	-311.8956	22.07	-311.9308	-311.8962	21.74	-0.33	-311.9314	-311.8974	21.32	-0.75
Free Energy	-311.9735	-311.9358	23.65	-311.9735	-311.9363	23.34	-0.30	-311.9741	-311.9376	22.90	-0.75
B3LYP-D3/def2-TZVP											
Electronic Energy	-312.1321	-312.0977	21.62	-312.1321	-312.0982	21.28	-0.34	-312.1327	-312.0995	20.86	-0.76
Enthalpy	-311.9460	-311.9134	20.46	-311.9460	-311.9139	20.13	-0.34	-311.9466	-311.9152	19.70	-0.76
Free Energy	-311.9886	-311.9536	21.96	-311.9886	-311.9541	21.65	-0.31	-311.9892	-311.9555	21.19	-0.77
M06-2X/def2-TZVP											
Electronic Energy	-311.9601	-311.9175	26.76	-311.9601	-311.9179	26.50	-0.26	-311.9608	-311.9190	26.20	-0.56
Enthalpy	-311.7724	-311.7313	25.76	-311.7724	-311.7318	25.48	-0.28	-311.7731	-311.7329	25.20	-0.56
Free Energy	-311.8146	-311.7713	27.12	-311.8146	-311.7718	26.89	-0.23	-311.8153	-311.7727	26.78	-0.34
ωB97X-D/def2-TZVP											
Electronic Energy	-312.0117	-311.9656	28.94	-312.0117	-311.9660	28.64	-0.30	-312.0122	-311.9671	28.27	-0.67
Enthalpy	-311.8240	-311.7795	27.88	-311.8240	-311.7799	27.70	-0.18	-311.8246	-311.7810	27.32	-0.56
Free Energy	-311.8665	-311.8193	29.58	-311.8665	-311.8195	29.46	-0.11	-311.8671	-311.8207	29.09	-0.49

Table S31. Summary of A.V.E.D.A. output data for Reaction 5 computed in the gas phase at 298 K, continued

Level of Theory	F = 0 a.u.			F = 7.5 10^{-3} a.u.				F = 10.0 10^{-3} a.u.			
	E_{Int}	E_{TS}	$\Delta E^{\ddagger}$	E_{Int}	E_{TS}	$\Delta E^{\ddagger}$	$\Delta\Delta E^{\ddagger}$	E_{Int}	E_{TS}	$\Delta E^{\ddagger}$	$\Delta\Delta E^{\ddagger}$
B3LYP/def2-TZVP											
Electronic Energy	-312.1168	-312.0798	23.23	-312.1185	-312.0835	21.97	-1.26	-312.1203	-312.0862	21.37	-1.86
Enthalpy	-311.9308	-311.8956	22.07	-311.9326	-311.8995	20.81	-1.26	-311.9345	-311.9022	20.21	-1.86
Free Energy	-311.9735	-311.9358	23.65	-311.9753	-311.9397	22.35	-1.30	-311.9772	-311.9426	21.73	-1.92
B3LYP-D3/def2-TZVP											
Electronic Energy	-312.1321	-312.0977	21.62	-312.1339	-312.1014	20.34	-1.28	-312.1356	-312.1042	19.72	-1.89
Enthalpy	-311.9460	-311.9134	20.46	-311.9478	-311.9172	19.18	-1.28	-311.9496	-311.9200	18.58	-1.89
Free Energy	-311.9886	-311.9536	21.96	-311.9905	-311.9576	20.65	-1.31	-311.9923	-311.9604	20.07	-1.89
M06-2X/def2-TZVP											
Electronic Energy	-311.9601	-311.9175	26.76	-311.9621	-311.9208	25.88	-0.88	-311.9639	-311.9233	25.52	-1.24
Enthalpy	-311.7724	-311.7313	25.76	-311.7744	-311.7347	24.89	-0.87	-311.7764	-311.7373	24.54	-1.22
Free Energy	-311.8146	-311.7713	27.12	-311.8168	-311.7744	26.57	-0.56	-311.8188	-311.7770	26.26	-0.86
ωB97X-D/def2-TZVP											
Electronic Energy	-312.0117	-311.9656	28.94	-312.0133	-311.9690	27.82	-1.12	-312.0150	-311.9715	27.29	-1.66
Enthalpy	-311.8240	-311.7795	27.88	-311.8257	-311.7829	26.85	-1.03	-311.8275	-311.7855	26.29	-1.58
Free Energy	-311.8665	-311.8193	29.58	-311.8682	-311.8226	28.61	-0.96	-311.8700	-311.8253	28.06	-1.52

4.6 Reaction 6

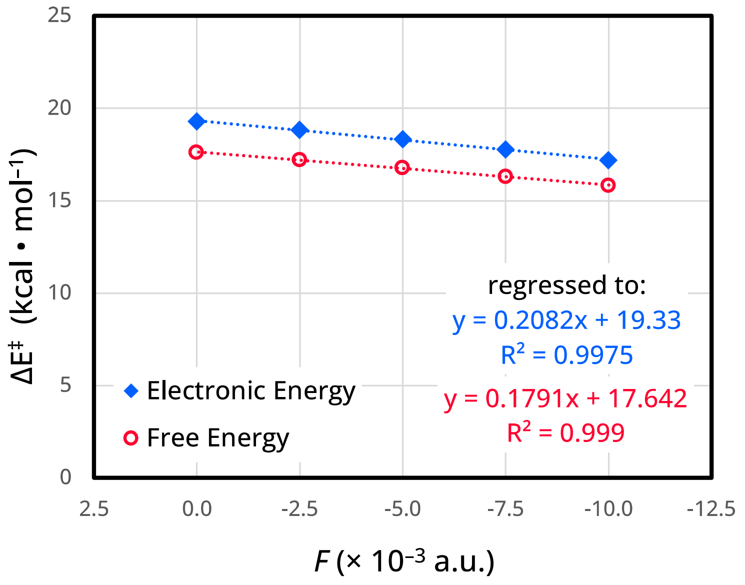

Figure S24. Effective activation energy (Δ*E*‡ or Δ*G*‡, kcal/mol) as a function of OEF magnitude for Reaction 6 computed at the B3LYP/def2-TZVP level of theory (gas phase, 298 K).

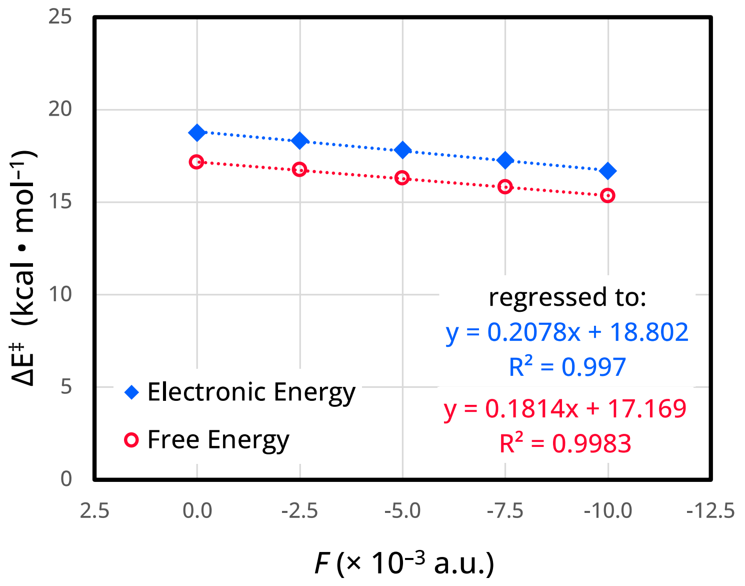

Figure S25. Effective activation energy (Δ*E*‡ or Δ*G*‡, kcal/mol) as a function of OEF magnitude for Reaction 6 computed at the B3LYP-D3/def2-TZVP level of theory (gas phase, 298 K).

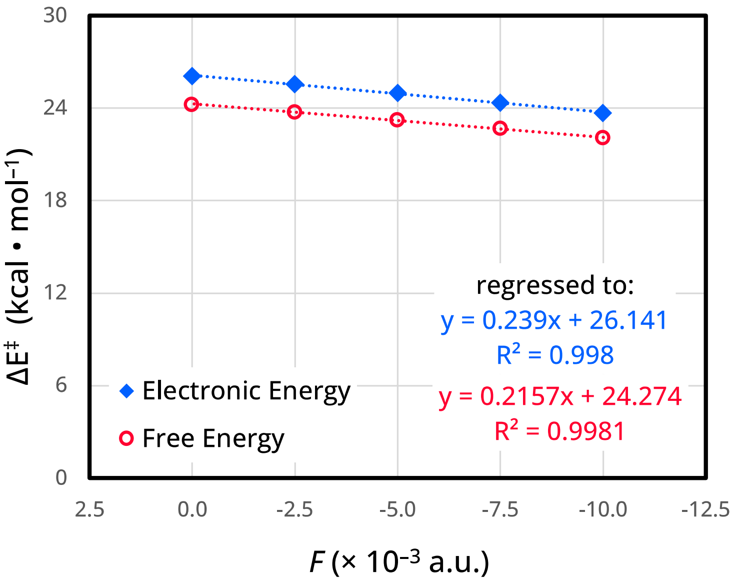

Figure S26. Effective activation energy ($\Delta E^‡$ or $\Delta G^‡$, kcal/mol) as a function of OEF magnitude for Reaction 6 computed at the M06-2X/def2-TZVP level of theory (gas phase, 298 K).

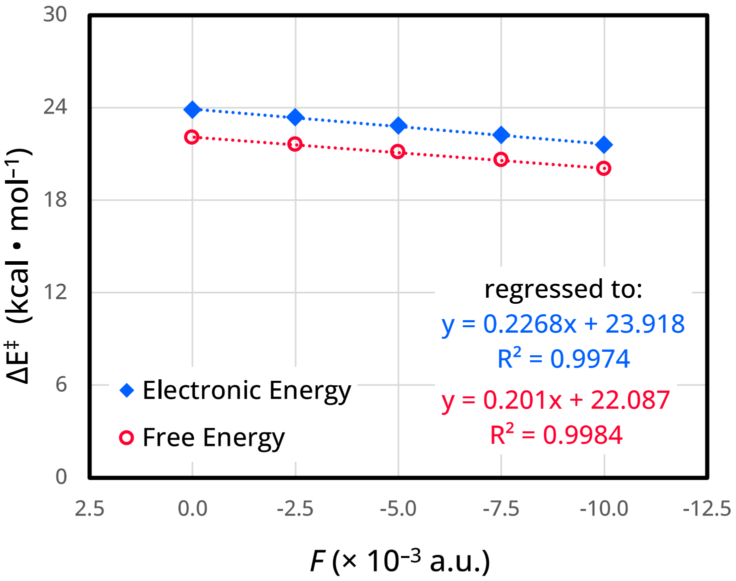

Figure S27. Effective activation energy ($\Delta E^‡$ or $\Delta G^‡$, kcal/mol) as a function of OEF magnitude for Reaction 6 computed at the ωB97X-D/def2-TZVP level of theory (gas phase, 298 K).

Table S32. Summary of A.V.E.D.A. output data for Reaction 6 computed at the B3LYP/def2-TZVP level of theory (gas phase, 298 K).

**Dipole Moments (debeye)**					
μ(Int) (x,y,z)	[0.557, 0.3386, 0.3041]			‖μ(Int)‖	0.7193
μ(TS) (x,y,z)	[0.521, 0.9317, -0.0619]			‖μ(TS)‖	1.0693
μ(rxn) (x,y,z)	[-0.0360, 0.5931, -0.366]			‖μ(rxn)‖	0.6979

**Oriented Electric Field**					
F [× 10^{-3} a.u.]	0	-2.5	-5	-7.5	-10
**Electronic Energies**					
Int [a.u.]	-269.4225	-269.4228	-269.4234	-269.4245	-269.4259
TS [a.u.]	-269.3918	-269.3928	-269.3942	-269.3961	-269.3985
$\Delta E^{\ddagger}$ [kcal/mol]	19.28	18.83	18.33	17.79	17.20
$\Delta\Delta E^{\ddagger}$ [kcal/mol]	0.00	-0.45	-0.95	-1.50	-2.08
**Free Energies**					
Int [a.u.]	-269.3523	-269.3526	-269.3534	-269.3546	-269.3562
TS [a.u.]	-269.3242	-269.3252	-269.3267	-269.3286	-269.3310
$\Delta G^{\ddagger}$ [kcal/mol]	17.62	17.21	16.77	16.30	15.83
$\Delta\Delta G^{\ddagger}$ [kcal/mol]	0.00	-0.40	-0.84	-1.31	-1.79
**RMSD from 0-Field**					
Int (Å)	-	0.0132	0.0288	0.0480	0.0729
TS (Å)	-	0.0074	0.0151	0.0232	0.0318

Table S33. Summary of A.V.E.D.A. output data for Reaction 6 computed at the B3LYP-D3/def2-TZVP level of theory (gas phase, 298 K).

**Dipole Moments (debeye)**					
μ(Int) (x,y,z)	[0.5552, 0.3446, 0.3089]			‖μ(Int)‖	0.7228
μ(TS) (x,y,z)	[0.5205, 0.9162, -0.0645]			‖μ(TS)‖	1.0557
μ(rxn) (x,y,z)	[-0.0347, 0.5716, -0.3734]			‖μ(rxn)‖	0.6836
**Oriented Electric Field**					
F [× 10^{-3} a.u.]	0	-2.5	-5	-7.5	-10
**Electronic Energies**					
Int [a.u.]	-269.4286	-269.4288	-269.4295	-269.4305	-269.4319
TS [a.u.]	-269.3987	-269.3997	-269.4011	-269.4030	-269.4053
$\Delta E^{\ddagger}$ [kcal/mol]	18.75	18.31	17.81	17.27	16.68
$\Delta\Delta E^{\ddagger}$ [kcal/mol]	0.00	-0.45	-0.94	-1.49	-2.08
**Free Energies**					
Int [a.u.]	-269.3584	-269.3587	-269.3594	-269.3606	-269.3622
TS [a.u.]	-269.3311	-269.3320	-269.3335	-269.3354	-269.3378
$\Delta G^{\ddagger}$ [kcal/mol]	17.14	16.73	16.29	15.82	15.33
$\Delta\Delta G^{\ddagger}$ [kcal/mol]	0.00	-0.40	-0.84	-1.32	-1.81
**RMSD from 0-Field**					
Int (Å)	-	0.0117	0.0267	0.0449	0.0672
TS (Å)	-	0.0073	0.0151	0.0233	0.0321

Table S34. Summary of A.V.E.D.A. output data for Reaction 6 computed at the M06-2X/def2-TZVP level of theory (gas phase, 298 K).

**Dipole Moments (debeye)**					
μ(Int) (x,y,z)	[0.5633, 0.3629, 0.3664]			||μ(Int)||	0.7637
μ(TS) (x,y,z)	[0.4155, 1.0308, -0.0841]			||μ(TS)||	1.1146
μ(rxn) (x,y,z)	[-0.1478, 0.6679, -0.4505]			||μ(rxn)||	0.8191

**Oriented Electric Field**					
F [× 10^{-3} a.u.]	0	-2.5	-5	-7.5	-10
**Electronic Energies**					
Int [a.u.]	-269.3014	-269.3015	-269.3020	-269.3029	-269.3041
TS [a.u.]	-269.2598	-269.2608	-269.2622	-269.2640	-269.2663
$\Delta E^{‡}$ [kcal/mol]	26.09	25.57	24.99	24.37	23.71
$\Delta\Delta E^{‡}$ [kcal/mol]	0.00	-0.53	-1.10	-1.72	-2.39
**Free Energies**					
Int [a.u.]	-269.2297	-269.2299	-269.2305	-269.2314	-269.2328
TS [a.u.]	-269.1911	-269.1920	-269.1934	-269.1953	-269.1976
$\Delta G^{‡}$ [kcal/mol]	24.23	23.76	23.24	22.68	22.08
$\Delta\Delta G^{‡}$ [kcal/mol]	0.00	-0.48	-1.00	-1.56	-2.16
**RMSD from 0-Field**					
Int (Å)	-	0.0090	0.0204	0.0337	0.0502
TS (Å)	-	0.0067	0.0132	0.0199	0.0270

Table S35. Summary of A.V.E.D.A. output data for Reaction 6 computed at the ωB97X-D/def2-TZVP level of theory (gas phase, 298 K).

**Dipole Moments (debeye)**					
μ(Int) (x,y,z)	[0.552, 0.3046, 0.3166]			‖μ(Int)‖	0.7055
μ(TS) (x,y,z)	[0.4617, 0.9468, -0.0743]			‖μ(TS)‖	1.0560
μ(rxn) (x,y,z)	[-0.0903, 0.6422, -0.3909]			‖μ(rxn)‖	0.7572

**Oriented Electric Field**					
F [× 10^{-3} a.u.]	0	-2.5	-5	-7.5	-10
**Electronic Energies**					
Int [a.u.]	-269.3249	-269.3251	-269.3257	-269.3266	-269.3278
TS [a.u.]	-269.2869	-269.2879	-269.2893	-269.2911	-269.2934
$\Delta E^{‡}$ [kcal/mol]	23.87	23.38	22.83	22.24	21.60
$\Delta\Delta E^{‡}$ [kcal/mol]	0.00	-0.49	-1.04	-1.63	-2.27
**Free Energies**					
Int [a.u.]	-269.2535	-269.2537	-269.2544	-269.2554	-269.2568
TS [a.u.]	-269.2183	-269.2193	-269.2207	-269.2225	-269.2248
$\Delta G^{‡}$ [kcal/mol]	22.05	21.60	21.12	20.60	20.04
$\Delta\Delta G^{‡}$ [kcal/mol]	0.00	-0.45	-0.94	-1.46	-2.01
**RMSD from 0-Field**					
Int (Å)	-	0.0089	0.0230	0.0392	0.0581
TS (Å)	-	0.0068	0.0137	0.0208	0.0284

Table S36. Summary of A.V.E.D.A. output data for Reaction 6 computed in the gas phase at 298 K.

Level of Theory	F = 0 a.u.			F = 2.5 10^{-3} a.u.				F = 5.0 10^{-3} a.u.			
	E_{Int}	E_{TS}	$\Delta E^{\ddagger}$	E_{Int}	E_{TS}	$\Delta E^{\ddagger}$	$\Delta\Delta E^{\ddagger}$	E_{Int}	E_{TS}	$\Delta E^{\ddagger}$	$\Delta\Delta E^{\ddagger}$
B3LYP/def2-TZVP											
Electronic Energy	-269.4225	-269.3918	19.29	-269.4228	-269.3928	18.83	-0.45	-269.4234	-269.3942	18.33	-0.95
Enthalpy	-269.3183	-269.2904	17.53	-269.3186	-269.2914	17.10	-0.43	-269.3193	-269.2928	16.63	-0.90
Free Energy	-269.3523	-269.3242	17.62	-269.3526	-269.3252	17.21	-0.40	-269.3534	-269.3267	16.77	-0.84
B3LYP-D3/def2-TZVP											
Electronic Energy	-269.4286	-269.3987	18.75	-269.4288	-269.3997	18.31	-0.45	-269.4295	-269.4011	17.81	-0.94
Enthalpy	-269.3244	-269.2973	17.04	-269.3247	-269.2982	16.61	-0.43	-269.3254	-269.2997	16.14	-0.90
Free Energy	-269.3584	-269.3311	17.14	-269.3587	-269.3320	16.73	-0.40	-269.3594	-269.3335	16.29	-0.84
M06-2X/def2-TZVP											
Electronic Energy	-269.3014	-269.2598	26.10	-269.3015	-269.2608	25.57	-0.53	-269.3020	-269.2622	24.99	-1.10
Enthalpy	-269.1959	-269.1573	24.17	-269.1960	-269.1583	23.68	-0.50	-269.1966	-269.1597	23.12	-1.05
Free Energy	-269.2297	-269.1911	24.24	-269.2299	-269.1920	23.76	-0.48	-269.2305	-269.1934	23.24	-1.00
ωB97X-D/def2-TZVP											
Electronic Energy	-269.3249	-269.2869	23.87	-269.3251	-269.2879	23.38	-0.49	-269.3257	-269.2893	22.83	-1.04
Enthalpy	-269.2196	-269.1846	21.98	-269.2198	-269.1856	21.51	-0.47	-269.2204	-269.1869	20.99	-0.99
Free Energy	-269.2535	-269.2183	22.05	-269.2537	-269.2193	21.60	-0.45	-269.2544	-269.2207	21.12	-0.94

Table S37. Summary of A.V.E.D.A. output data for Reaction 6 computed in the gas phase at 298 K, continued

Level of Theory	F = 0 a.u.			F = 7.5 10^{-3} a.u.				F = 10.0 10^{-3} a.u.			
	E_{Int}	E_{TS}	$\Delta E^{‡}$	E_{Int}	E_{TS}	$\Delta E^{‡}$	$\Delta\Delta E^{‡}$	E_{Int}	E_{TS}	$\Delta E^{‡}$	$\Delta\Delta E^{‡}$
B3LYP/def2-TZVP											
Electronic Energy	-269.4225	-269.3918	19.29	-269.4245	-269.3961	17.79	-1.50	-269.4259	-269.3985	17.20	-2.08
Enthalpy	-269.3183	-269.2904	17.53	-269.3204	-269.2947	16.10	-1.43	-269.3218	-269.2971	15.54	-1.99
Free Energy	-269.3523	-269.3242	17.62	-269.3546	-269.3286	16.30	-1.31	-269.3562	-269.3310	15.83	-1.79
B3LYP-D3/def2-TZVP											
Electronic Energy	-269.4286	-269.3987	18.75	-269.4305	-269.4030	17.27	-1.49	-269.4319	-269.4053	16.68	-2.08
Enthalpy	-269.3244	-269.2973	17.04	-269.3264	-269.3015	15.62	-1.42	-269.3279	-269.3039	15.05	-1.99
Free Energy	-269.3584	-269.3311	17.14	-269.3606	-269.3354	15.82	-1.32	-269.3622	-269.3378	15.33	-1.81
M06-2X/def2-TZVP											
Electronic Energy	-269.3014	-269.2598	26.10	-269.3029	-269.2640	24.37	-1.72	-269.3041	-269.2663	23.71	-2.39
Enthalpy	-269.1959	-269.1573	24.17	-269.1974	-269.1615	22.52	-1.65	-269.1987	-269.1638	21.88	-2.30
Free Energy	-269.2297	-269.1911	24.24	-269.2314	-269.1953	22.68	-1.56	-269.2328	-269.1976	22.08	-2.16
ωB97X-D/def2-TZVP											
Electronic Energy	-269.3249	-269.2869	23.87	-269.3266	-269.2911	22.24	-1.63	-269.3278	-269.2934	21.60	-2.27
Enthalpy	-269.2196	-269.1846	21.98	-269.2213	-269.1888	20.42	-1.56	-269.2226	-269.1911	19.80	-2.17
Free Energy	-269.2535	-269.2183	22.05	-269.2554	-269.2225	20.60	-1.46	-269.2568	-269.2248	20.04	-2.01

4.7 Reaction 7

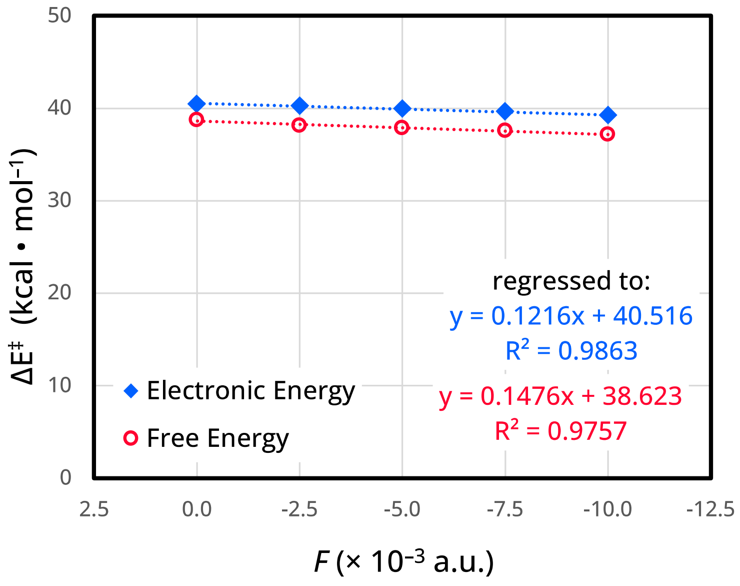

Figure S28. Effective activation energy ($\Delta E^{\ddagger}$ or $\Delta G^{\ddagger}$, kcal/mol) as a function of OEF magnitude for Reaction 7 computed at the B3LYP/def2-TZVP level of theory (gas phase, 298 K).

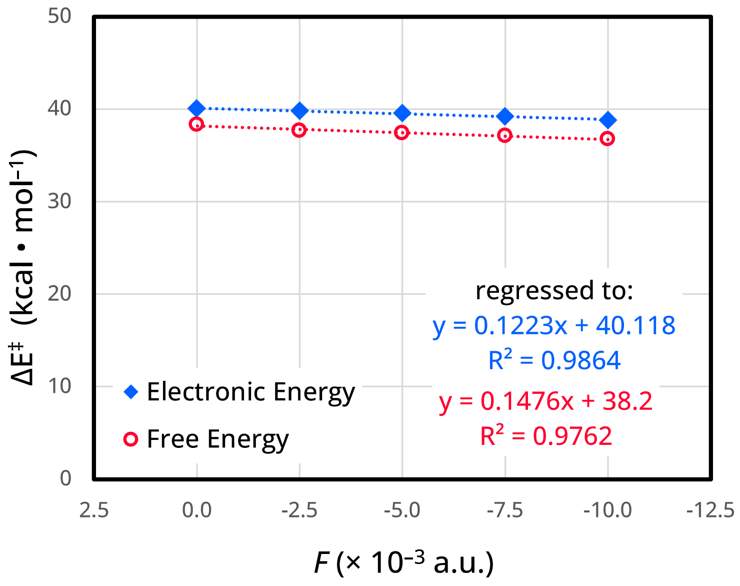

Figure S29. Effective activation energy ($\Delta E^{\ddagger}$ or $\Delta G^{\ddagger}$, kcal/mol) as a function of OEF magnitude for Reaction 7 computed at the B3LYP-D3/def2-TZVP level of theory (gas phase, 298 K).

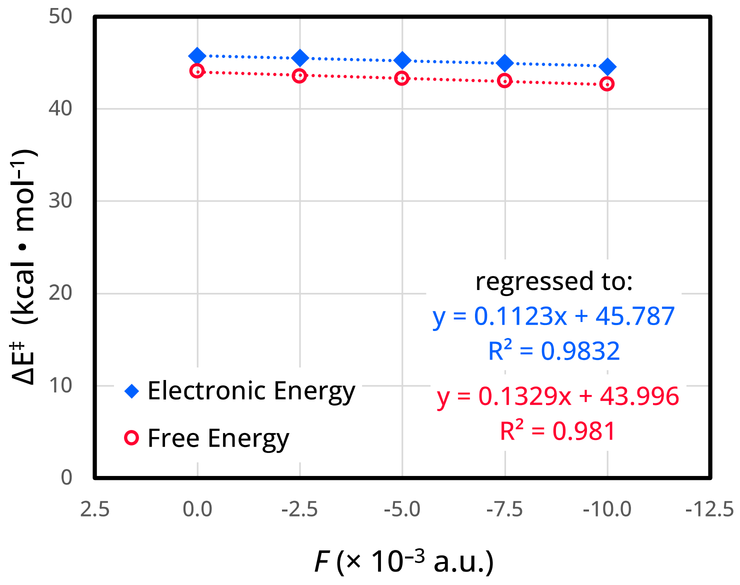

Figure S30. Effective activation energy ($\Delta E^\ddagger$ or $\Delta G^\ddagger$, kcal/mol) as a function of OEF magnitude for Reaction 7 computed at the M06-2X/def2-TZVP level of theory (gas phase, 298 K).

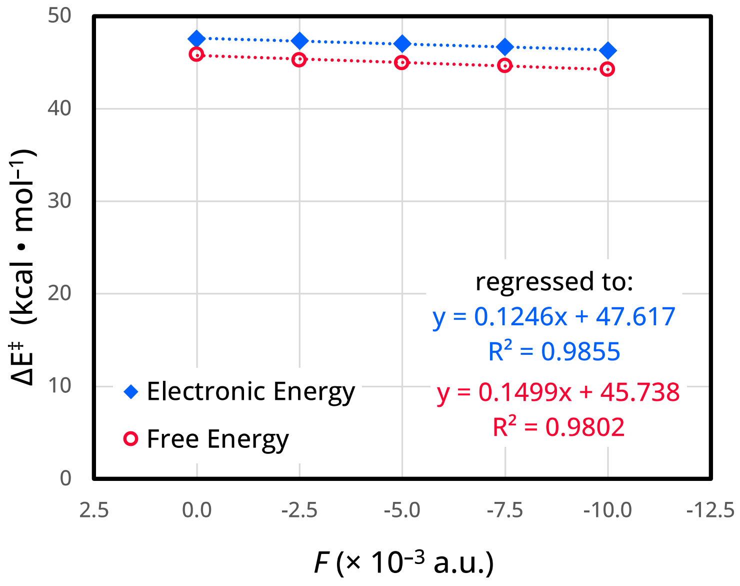

Figure S31. Effective activation energy ($\Delta E^\ddagger$ or $\Delta G^\ddagger$, kcal/mol) as a function of OEF magnitude for Reaction 7 computed at the ωB97X-D/def2-TZVP level of theory (gas phase, 298 K).

Table S38. Summary of A.V.E.D.A. output data for Reaction 7 computed at the B3LYP/def2-TZVP level of theory (gas phase, 298 K).

**Dipole Moments (debeye)**					
μ(Int) (x,y,z)	[0.0, -0.0, 0.4916]			‖μ(Int)‖	0.4916
μ(TS) (x,y,z)	[-0.0, 0.0, 0.1991]			‖μ(TS)‖	0.1991
μ(rxn) (x,y,z)	[-0.0, 0.0, -0.2925]			‖μ(rxn)‖	0.2925
**Oriented Electric Field**					
F [× 10^{-3} a.u.]	0	-2.5	-5	-7.5	-10
**Electronic Energies**					
Int [a.u.]	-309.7428	-309.7427	-309.7433	-309.7446	-309.7466
TS [a.u.]	-309.6783	-309.6786	-309.6796	-309.6814	-309.6840
$\Delta E^{\ddagger}$ [kcal/mol]	40.46	40.24	39.97	39.63	39.24
$\Delta\Delta E^{\ddagger}$ [kcal/mol]	0.00	-0.21	-0.49	-0.82	-1.21
**Free Energies**					
Int [a.u.]	-309.6386	-309.6385	-309.6391	-309.6404	-309.6425
TS [a.u.]	-309.5769	-309.5777	-309.5787	-309.5806	-309.5833
$\Delta G^{\ddagger}$ [kcal/mol]	38.73	38.12	37.86	37.54	37.18
$\Delta\Delta G^{\ddagger}$ [kcal/mol]	0.00	-0.62	-0.88	-1.19	-1.56
**RMSD from 0-Field**					
Int (Å)	-	0.0005	0.0010	0.0016	0.0021
TS (Å)	-	0.0035	0.0068	0.0101	0.0134

Table S39. Summary of A.V.E.D.A. output data for Reaction 7 computed at the B3LYP-D3/def2-TZVP level of theory (gas phase, 298 K).

**Dipole Moments (debeye)**					
μ(Int) (x,y,z)	[0.0, 0.0, 0.4923]			‖μ(Int)‖	0.4923
μ(TS) (x,y,z)	[-0.0, 0.0, 0.1973]			‖μ(TS)‖	0.1973
μ(rxn) (x,y,z)	[-0.0, 0.0, -0.2950]			‖μ(rxn)‖	0.2950

**Oriented Electric Field**					
F [× 10^{-3} a.u.]	0	-2.5	-5	-7.5	-10
**Electronic Energies**					
Int [a.u.]	-309.7517	-309.7515	-309.7521	-309.7534	-309.7554
TS [a.u.]	-309.6878	-309.6880	-309.6890	-309.6909	-309.6935
$\Delta E^{\ddagger}$ [kcal/mol]	40.06	39.84	39.57	39.23	38.83
$\Delta\Delta E^{\ddagger}$ [kcal/mol]	0.00	-0.21	-0.49	-0.83	-1.22
**Free Energies**					
Int [a.u.]	-309.6475	-309.6473	-309.6479	-309.6493	-309.6514
TS [a.u.]	-309.5864	-309.5873	-309.5883	-309.5901	-309.5928
$\Delta G^{\ddagger}$ [kcal/mol]	38.31	37.70	37.43	37.12	36.75
$\Delta\Delta G^{\ddagger}$ [kcal/mol]	0.00	-0.62	-0.88	-1.19	-1.56
**RMSD from 0-Field**					
Int (Å)	-	0.0007	0.0014	0.0022	0.0031
TS (Å)	-	0.0034	0.0068	0.0101	0.0135

Table S40. Summary of A.V.E.D.A. output data for Reaction 7 computed at the M06-2X/def2-TZVP level of theory (gas phase, 298 K).

**Dipole Moments (debeye)**					
μ(Int) (x,y,z)	[0.0, 0.0, 0.5009]			||μ(Int)||	0.5009
μ(TS) (x,y,z)	[0.0, -0.0, 0.2496]			||μ(TS)||	0.2496
μ(rxn) (x,y,z)	[0.0, -0.0, -0.2513]			||μ(rxn)||	0.2513

**Oriented Electric Field**					
F [× 10^{-3} a.u.]	0	-2.5	-5	-7.5	-10
**Electronic Energies**					
Int [a.u.]	-309.5990	-309.5988	-309.5994	-309.6006	-309.6025
TS [a.u.]	-309.5261	-309.5263	-309.5272	-309.5289	-309.5315
$\Delta E^{\ddagger}$ [kcal/mol]	45.72	45.54	45.29	44.97	44.60
$\Delta\Delta E^{\ddagger}$ [kcal/mol]	0.00	-0.19	-0.44	-0.75	-1.12
**Free Energies**					
Int [a.u.]	-309.4933	-309.4932	-309.4938	-309.4951	-309.4971
TS [a.u.]	-309.4231	-309.4238	-309.4248	-309.4265	-309.4291
$\Delta G^{\ddagger}$ [kcal/mol]	44.08	43.55	43.32	43.03	42.68
$\Delta\Delta G^{\ddagger}$ [kcal/mol]	0.00	-0.53	-0.77	-1.06	-1.40
**RMSD from 0-Field**					
Int (Å)	-	0.0004	0.0008	0.0013	0.0019
TS (Å)	-	0.0030	0.0062	0.0093	0.0124

Table S41. Summary of A.V.E.D.A. output data for Reaction 7 computed at the ωB97X-D/def2-TZVP level of theory (gas phase, 298 K).

**Dipole Moments (debeye)**					
μ(Int) (x,y,z)	[0.0, -0.0, 0.5085]			‖μ(Int)‖	0.5085
μ(TS) (x,y,z)	[0.0, -0.0, 0.2135]			‖μ(TS)‖	0.2135
μ(rxn) (x,y,z)	[0.0, 0.0, -0.295]			‖μ(rxn)‖	0.2950

**Oriented Electric Field**					
F [× 10^{-3} a.u.]	0	-2.5	-5	-7.5	-10
**Electronic Energies**					
Int [a.u.]	-309.6313	-309.6312	-309.6317	-309.6329	-309.6349
TS [a.u.]	-309.5555	-309.5557	-309.5567	-309.5585	-309.5611
$\Delta E^\ddagger$ [kcal/mol]	47.55	47.34	47.06	46.71	46.31
$\Delta\Delta E^\ddagger$ [kcal/mol]	0.00	-0.22	-0.50	-0.84	-1.25
**Free Energies**					
Int [a.u.]	-309.5256	-309.5254	-309.5260	-309.5273	-309.5293
TS [a.u.]	-309.4525	-309.4534	-309.4543	-309.4562	-309.4588
$\Delta G^\ddagger$ [kcal/mol]	45.84	45.23	44.97	44.64	44.26
$\Delta\Delta G^\ddagger$ [kcal/mol]	0.00	-0.60	-0.87	-1.20	-1.58
**RMSD from 0-Field**					
Int (Å)	-	0.0004	0.0009	0.0014	0.0020
TS (Å)	-	0.0035	0.0067	0.0099	0.0130

Table S42. Summary of A.V.E.D.A. output data for Reaction 7 computed in the gas phase at 298 K.

Level of Theory	F = 0 a.u.			F = 2.5 10^{-3} a.u.				F = 5.0 10^{-3} a.u.			
	E_{Int}	E_{TS}	$\Delta E^{\ddagger}$	E_{Int}	E_{TS}	$\Delta E^{\ddagger}$	$\Delta\Delta E^{\ddagger}$	E_{Int}	E_{TS}	$\Delta E^{\ddagger}$	$\Delta\Delta E^{\ddagger}$
B3LYP/def2-TZVP											
Electronic Energy	-309.7428	-309.6784	40.45	-309.7427	-309.6786	40.24	-0.21	-309.7433	-309.6796	39.97	-0.48
Enthalpy	-309.6019	-309.5408	38.34	-309.6017	-309.5409	38.14	-0.20	-309.6023	-309.5419	37.88	-0.46
Free Energy	-309.6386	-309.5769	38.74	-309.6385	-309.5777	38.12	-0.62	-309.6391	-309.5787	37.86	-0.88
B3LYP-D3/def2-TZVP											
Electronic Energy	-309.7517	-309.6878	40.06	-309.7515	-309.6880	39.84	-0.21	-309.7521	-309.6890	39.57	-0.49
Enthalpy	-309.6107	-309.5503	37.92	-309.6106	-309.5505	37.72	-0.20	-309.6112	-309.5515	37.46	-0.46
Free Energy	-309.6475	-309.5864	38.31	-309.6473	-309.5873	37.70	-0.62	-309.6479	-309.5883	37.43	-0.88
M06-2X/def2-TZVP											
Electronic Energy	-309.5990	-309.5261	45.73	-309.5988	-309.5263	45.54	-0.19	-309.5994	-309.5272	45.29	-0.44
Enthalpy	-309.4566	-309.3870	43.67	-309.4565	-309.3871	43.52	-0.14	-309.4571	-309.3881	43.29	-0.38
Free Energy	-309.4933	-309.4231	44.08	-309.4932	-309.4238	43.55	-0.53	-309.4938	-309.4248	43.32	-0.77
ωB97X-D/def2-TZVP											
Electronic Energy	-309.6313	-309.5555	47.55	-309.6312	-309.5557	47.34	-0.22	-309.6317	-309.5567	47.06	-0.50
Enthalpy	-309.4889	-309.4165	45.44	-309.4888	-309.4167	45.24	-0.20	-309.4893	-309.4177	44.97	-0.46
Free Energy	-309.5256	-309.4525	45.84	-309.5254	-309.4534	45.23	-0.60	-309.5260	-309.4543	44.97	-0.87

Table S43. Summary of A.V.E.D.A. output data for Reaction 7 computed in the gas phase at 298 K, continued.

Level of Theory	F = 0 a.u.			F = 7.5 10^{-3} a.u.				F = 10.0 10^{-3} a.u.			
	E_{Int}	E_{TS}	$\Delta E^{‡}$	E_{Int}	E_{TS}	$\Delta E^{‡}$	$\Delta\Delta E^{‡}$	E_{Int}	E_{TS}	$\Delta E^{‡}$	$\Delta\Delta E^{‡}$
B3LYP/def2-TZVP											
Electronic Energy	-309.7428	-309.6784	40.45	-309.7446	-309.6814	39.63	-0.82	-309.7466	-309.6840	39.24	-1.21
Enthalpy	-309.6019	-309.5408	38.34	-309.6037	-309.5438	37.57	-0.78	-309.6058	-309.5465	37.20	-1.14
Free Energy	-309.6386	-309.5769	38.74	-309.6404	-309.5806	37.54	-1.19	-309.6425	-309.5833	37.18	-1.56
B3LYP-D3/def2-TZVP											
Electronic Energy	-309.7517	-309.6878	40.06	-309.7534	-309.6909	39.23	-0.83	-309.7554	-309.6935	38.83	-1.22
Enthalpy	-309.6107	-309.5503	37.92	-309.6125	-309.5533	37.15	-0.78	-309.6146	-309.5560	36.78	-1.14
Free Energy	-309.6475	-309.5864	38.31	-309.6493	-309.5901	37.12	-1.19	-309.6514	-309.5928	36.75	-1.56
M06-2X/def2-TZVP											
Electronic Energy	-309.5990	-309.5261	45.73	-309.6006	-309.5289	44.98	-0.75	-309.6025	-309.5315	44.60	-1.12
Enthalpy	-309.4566	-309.3870	43.67	-309.4584	-309.3898	43.00	-0.67	-309.4604	-309.3924	42.65	-1.01
Free Energy	-309.4933	-309.4231	44.08	-309.4951	-309.4265	43.03	-1.06	-309.4971	-309.4291	42.68	-1.40
ωB97X-D/def2-TZVP											
Electronic Energy	-309.6313	-309.5555	47.55	-309.6329	-309.5585	46.71	-0.84	-309.6349	-309.5611	46.31	-1.25
Enthalpy	-309.4889	-309.4165	45.44	-309.4906	-309.4195	44.65	-0.79	-309.4926	-309.4221	44.27	-1.17
Free Energy	-309.5256	-309.4525	45.84	-309.5273	-309.4562	44.64	-1.20	-309.5293	-309.4588	44.26	-1.58

4.8 Reaction 8

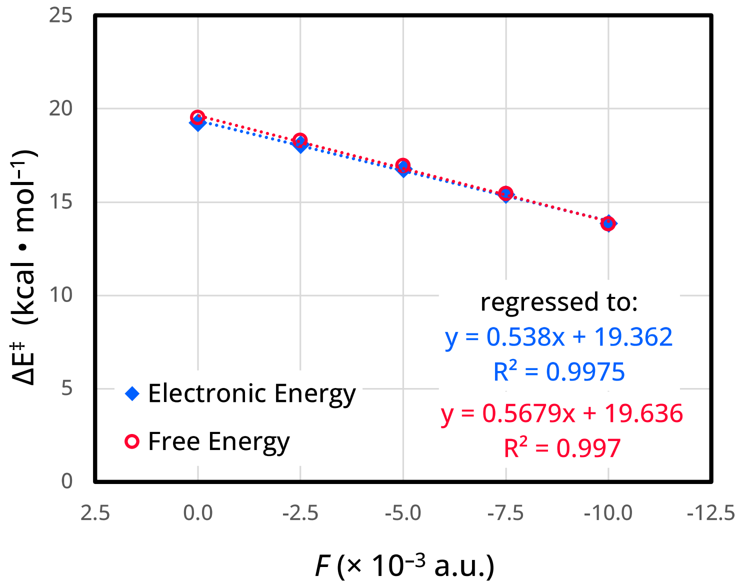

Figure S32. Effective activation energy ($\Delta E^‡$ or $\Delta G^‡$, kcal/mol) as a function of OEF magnitude for Reaction 8 computed at the B3LYP/def2-TZVP level of theory (gas phase, 298 K).

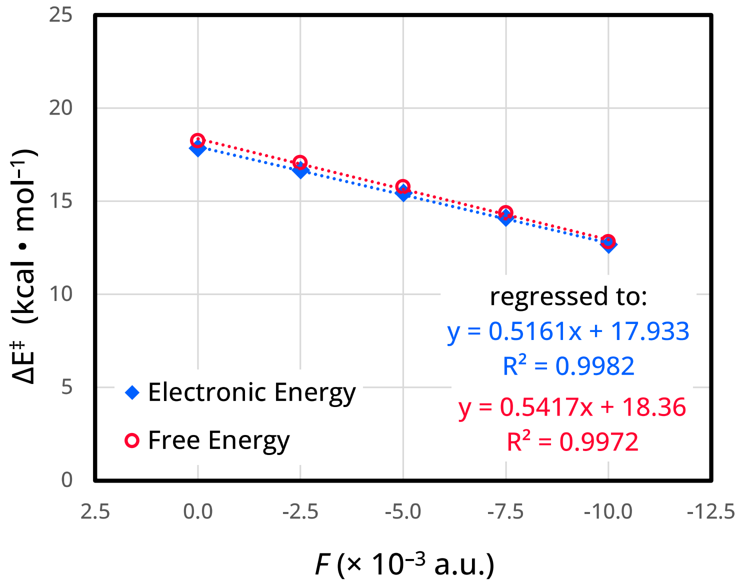

Figure S33. Effective activation energy ($\Delta E^‡$ or $\Delta G^‡$, kcal/mol) as a function of OEF magnitude for Reaction 8 computed at the B3LYP-D3/def2-TZVP level of theory (gas phase, 298 K).

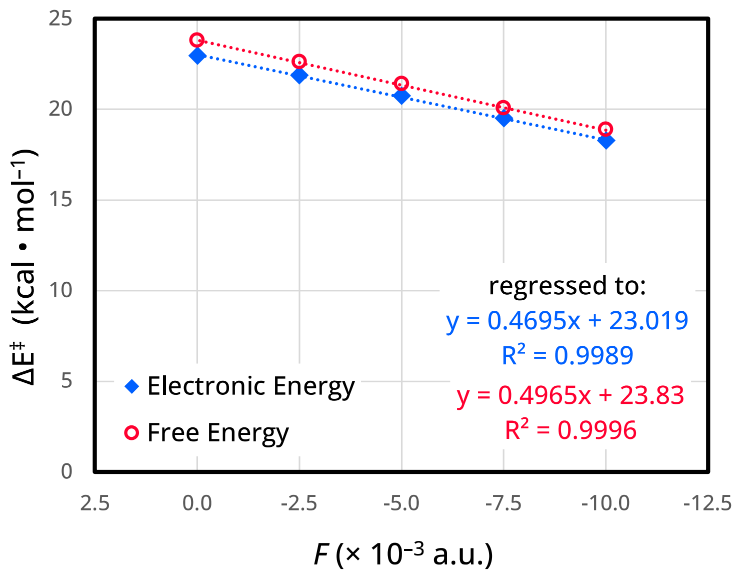

Figure S34. Effective activation energy ($\Delta E^{\ddagger}$ or $\Delta G^{\ddagger}$, kcal/mol) as a function of OEF magnitude for Reaction 8 computed at the M06-2X/def2-TZVP level of theory (gas phase, 298 K).

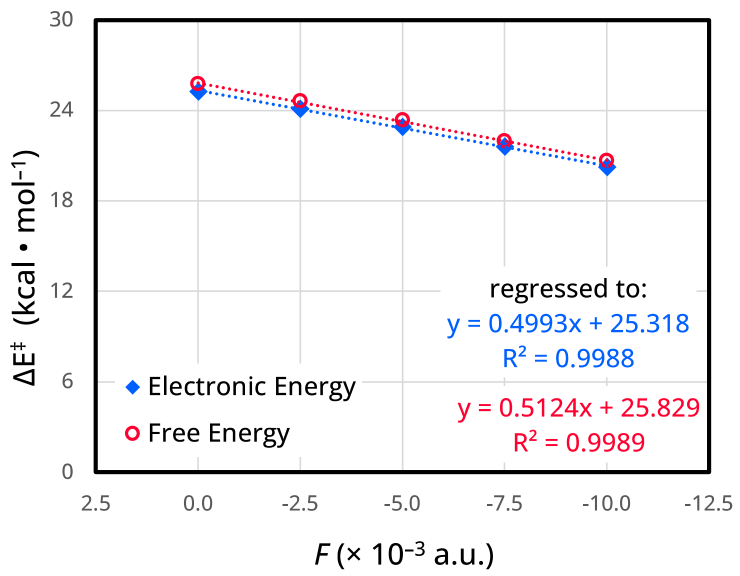

Figure S35. Effective activation energy ($\Delta E^{\ddagger}$ or $\Delta G^{\ddagger}$, kcal/mol) as a function of OEF magnitude for Reaction 8 computed at the ωB97X-D/def2-TZVP level of theory (gas phase, 298 K).

Table S44. Summary of A.V.E.D.A. output data for Reaction 8 computed at the B3LYP/def2-TZVP level of theory (gas phase, 298 K).

**Dipole Moments (debeye)**					
μ(Int) (x,y,z)	[-2.0923, -2.1313, -2.1068]			‖μ(Int)‖	3.6550
μ(TS) (x,y,z)	[-0.9621, -0.7469, -1.6144]			‖μ(TS)‖	2.0223
μ(rxn) (x,y,z)	[1.1302, 1.3844, 0.4924]			‖μ(rxn)‖	1.8537

**Oriented Electric Field**					
F [× 10^{-3} a.u.]	0	-2.5	-5	-7.5	-10
**Electronic Energies**					
Int [a.u.]	-822.5325	-822.5296	-822.5278	-822.5270	-822.5273
TS [a.u.]	-822.5018	-822.5008	-822.5010	-822.5024	-822.5052
$\Delta E^{\ddagger}$ [kcal/mol]	19.25	18.06	16.78	15.39	13.86
$\Delta\Delta E^{\ddagger}$ [kcal/mol]	0.00	-1.19	-2.47	-3.86	-5.39
**Free Energies**					
Int [a.u.]	-822.4046	-822.4018	-822.4001	-822.3995	-822.4000
TS [a.u.]	-822.3735	-822.3727	-822.3731	-822.3749	-822.3780
$\Delta G^{\ddagger}$ [kcal/mol]	19.50	18.29	16.93	15.44	13.83
$\Delta\Delta G^{\ddagger}$ [kcal/mol]	0.00	-1.22	-2.57	-4.06	-5.68
**RMSD from 0-Field**					
Int (Å)	-	0.0164	0.0472	0.0424	0.0722
TS (Å)	-	0.0304	0.0635	0.1065	0.1559

Table S45. Summary of A.V.E.D.A. output data for Reaction 8 computed at the B3LYP-D3/def2-TZVP level of theory (gas phase, 298 K).

**Dipole Moments (debeye)**					
μ(Int) (x,y,z)	[-2.1315, -1.9722, -2.1651]			‖μ(Int)‖	3.6222
μ(TS) (x,y,z)	[-0.9959, -0.6361, -1.6852]			‖μ(TS)‖	2.0582
μ(rxn) (x,y,z)	[1.1356, 1.3361, 0.4799]			‖μ(rxn)‖	1.8180

**Oriented Electric Field**					
F [× 10^{-3} a.u.]	0	-2.5	-5	-7.5	-10
**Electronic Energies**					
Int [a.u.]	-822.5498	-822.5470	-822.5453	-822.5446	-822.5451
TS [a.u.]	-822.5214	-822.5204	-822.5207	-822.5221	-822.5249
$\Delta E^{\ddagger}$ [kcal/mol]	17.84	16.68	15.45	14.12	12.68
$\Delta\Delta E^{\ddagger}$ [kcal/mol]	0.00	-1.16	-2.40	-3.73	-5.17
**Free Energies**					
Int [a.u.]	-822.4217	-822.4191	-822.4174	-822.4170	-822.4177
TS [a.u.]	-822.3927	-822.3919	-822.3923	-822.3941	-822.3972
$\Delta G^{\ddagger}$ [kcal/mol]	18.24	17.07	15.77	14.36	12.82
$\Delta\Delta G^{\ddagger}$ [kcal/mol]	0.00	-1.16	-2.47	-3.88	-5.42
**RMSD from 0-Field**					
Int (Å)	-	0.0190	0.0464	0.0832	0.1334
TS (Å)	-	0.0337	0.0678	0.1139	0.1616

Table S46. Summary of A.V.E.D.A. output data for Reaction 8 computed at the M06-2X/def2-TZVP level of theory (gas phase, 298 K).

**Dipole Moments (debeye)**					
μ(Int) (x,y,z)	[-2.0905, -2.0685, -2.2783]			‖μ(Int)‖	3.7202
μ(TS) (x,y,z)	[-1.4199, -0.766, -1.511]			‖μ(TS)‖	2.2104
μ(rxn) (x,y,z)	[0.6706, 1.3025, 0.7673]			‖μ(rxn)‖	1.8180

**Oriented Electric Field**					
F [× 10^{-3} a.u.]	0	-2.5	-5	-7.5	-10
**Electronic Energies**					
Int [a.u.]	-822.3051	-822.3022	-822.3001	-822.2992	-822.2995
TS [a.u.]	-822.2686	-822.2673	-822.2671	-822.2681	-822.2704
$\Delta E^{\ddagger}$ [kcal/mol]	22.95	21.89	20.74	19.51	18.27
$\Delta\Delta E^{\ddagger}$ [kcal/mol]	0.00	-1.06	-2.21	-3.44	-4.68
**Free Energies**					
Int [a.u.]	-822.1753	-822.1723	-822.1703	-822.1695	-822.1702
TS [a.u.]	-822.1374	-822.1362	-822.1362	-822.1375	-822.1402
$\Delta G^{\ddagger}$ [kcal/mol]	23.7979	22.6157	21.3946	20.0593	18.8702
$\Delta\Delta G^{\ddagger}$ [kcal/mol]	0.00	-1.18	-2.40	-3.74	-4.93
**RMSD from 0-Field**					
Int (Å)	-	0.0428	0.0916	0.1849	0.3627
TS (Å)	-	0.0339	0.0825	0.1429	0.2130

Table S47. Summary of A.V.E.D.A. output data for Reaction 8 computed at the ωB97X-D/def2-TZVP level of theory (gas phase, 298 K).

**Dipole Moments (debeye)**					
μ(Int) (x,y,z)	[-2.0745, -1.9643, -2.3761]			‖μ(Int)‖	3.7159
μ(TS) (x,y,z)	[-1.1994, -0.6343, -1.6181]			‖μ(TS)‖	2.1117
μ(rxn) (x,y,z)	[0.8751, 1.33, 0.758]			‖μ(rxn)‖	1.7633

**Oriented Electric Field**					
F [× 10^{-3} a.u.]	0	-2.5	-5	-7.5	-10
**Electronic Energies**					
Int [a.u.]	-822.3604	-822.3574	-822.3554	-822.3544	-822.3546
TS [a.u.]	-822.3201	-822.3190	-822.3189	-822.3200	-822.3223
$\Delta E^{\ddagger}$ [kcal/mol]	25.24	24.11	22.90	21.61	20.25
$\Delta\Delta E^{\ddagger}$ [kcal/mol]	0.00	-1.13	-2.34	-3.64	-4.99
**Free Energies**					
Int [a.u.]	-822.2303	-822.2274	-822.2255	-822.2246	-822.2252
TS [a.u.]	-822.1893	-822.1882	-822.1883	-822.1896	-822.1923
$\Delta G^{\ddagger}$ [kcal/mol]	25.75	24.60	23.35	21.97	20.66
$\Delta\Delta G^{\ddagger}$ [kcal/mol]	0.00	-1.15	-2.40	-3.78	-5.09
**RMSD from 0-Field**					
Int (Å)	-	0.0210	0.0815	0.1486	0.2897
TS (Å)	-	0.0391	0.0734	0.1277	0.1903

Table S48. Summary of A.V.E.D.A. output data for Reaction 8 computed in the gas phase at 298 K.

Level of Theory	F = 0 a.u.			F = 2.5 10^{-3} a.u.				F = 5.0 10^{-3} a.u.			
	E_{Int}	E_{TS}	$\Delta E^{‡}$	E_{Int}	E_{TS}	$\Delta E^{‡}$	$\Delta\Delta E^{‡}$	E_{Int}	E_{TS}	$\Delta E^{‡}$	$\Delta\Delta E^{‡}$
B3LYP/def2-TZVP											
Electronic Energy	-822.5325	-822.5018	19.25	-822.5296	-822.5008	18.07	-1.19	-822.5278	-822.5010	16.79	-2.47
Enthalpy	-822.3548	-822.3259	18.14	-822.3521	-822.3251	16.95	-1.19	-822.3504	-822.3254	15.64	-2.50
Free Energy	-822.4046	-822.3735	19.50	-822.4018	-822.3727	18.29	-1.22	-822.4001	-822.3731	16.93	-2.57
B3LYP-D3/def2-TZVP											
Electronic Energy	-822.5498	-822.5214	17.84	-822.5470	-822.5204	16.68	-1.16	-822.5453	-822.5207	15.45	-2.40
Enthalpy	-822.3720	-822.3453	16.79	-822.3694	-822.3445	15.63	-1.16	-822.3678	-822.3449	14.38	-2.41
Free Energy	-822.4217	-822.3927	18.24	-822.4191	-822.3919	17.07	-1.16	-822.4174	-822.3923	15.77	-2.47
M06-2X/def2-TZVP											
Electronic Energy	-822.3051	-822.2686	22.95	-822.3022	-822.2673	21.89	-1.06	-822.3001	-822.2671	20.74	-2.21
Enthalpy	-822.1258	-822.0907	22.02	-822.1228	-822.0895	20.93	-1.09	-822.1209	-822.0894	19.76	-2.26
Free Energy	-822.1753	-822.1374	23.80	-822.1723	-822.1362	22.62	-1.18	-822.1703	-822.1362	21.39	-2.40
ωB97X-D/def2-TZVP											
Electronic Energy	-822.3604	-822.3201	25.24	-822.3574	-822.3190	24.11	-1.13	-822.3554	-822.3189	22.90	-2.34
Enthalpy	-822.1808	-822.1424	24.15	-822.1779	-822.1413	23.00	-1.14	-822.1760	-822.1413	21.78	-2.36
Free Energy	-822.2303	-822.1893	25.75	-822.2274	-822.1882	24.60	-1.15	-822.2255	-822.1883	23.35	-2.40

Table S49. Summary of A.V.E.D.A. output data for Reaction 8 computed in the gas phase at 298 K, continued.

Level of Theory	F = 0 a.u.			F = 7.5 10^{-3} a.u.				F = 10.0 10^{-3} a.u.			
	E_{Int}	E_{TS}	$\Delta E^{\ddagger}$	E_{Int}	E_{TS}	$\Delta E^{\ddagger}$	$\Delta\Delta E^{\ddagger}$	E_{Int}	E_{TS}	$\Delta E^{\ddagger}$	$\Delta\Delta E^{\ddagger}$
B3LYP/def2-TZVP											
Electronic Energy	-822.5325	-822.5018	19.25	-822.5270	-822.5024	15.39	-3.86	-822.5273	-822.5052	13.86	-5.39
Enthalpy	-822.3548	-822.3259	18.14	-822.3497	-822.3271	14.22	-3.92	-822.3503	-822.3301	12.66	-5.48
Free Energy	-822.4046	-822.3735	19.50	-822.3995	-822.3749	15.44	-4.06	-822.4000	-822.3780	13.83	-5.68
B3LYP-D3/def2-TZVP											
Electronic Energy	-822.5498	-822.5214	17.84	-822.5446	-822.5221	14.12	-3.73	-822.5451	-822.5249	12.68	-5.17
Enthalpy	-822.3720	-822.3453	16.79	-822.3673	-822.3466	13.02	-3.77	-822.3680	-822.3496	11.55	-5.24
Free Energy	-822.4217	-822.3927	18.24	-822.4170	-822.3941	14.36	-3.88	-822.4177	-822.3972	12.82	-5.42
M06-2X/def2-TZVP											
Electronic Energy	-822.3051	-822.2686	22.95	-822.2992	-822.2681	19.51	-3.44	-822.2995	-822.2704	18.27	-4.68
Enthalpy	-822.1258	-822.0907	22.02	-822.1201	-822.0906	18.50	-3.52	-822.1206	-822.0932	17.20	-4.82
Free Energy	-822.1753	-822.1374	23.80	-822.1695	-822.1375	20.06	-3.74	-822.1702	-822.1402	18.87	-4.93
ωB97X-D/def2-TZVP											
Electronic Energy	-822.3604	-822.3201	25.24	-822.3544	-822.3200	21.61	-3.64	-822.3546	-822.3223	20.25	-4.99
Enthalpy	-822.1808	-822.1424	24.15	-822.1752	-822.1426	20.46	-3.69	-822.1756	-822.1452	19.09	-5.06
Free Energy	-822.2303	-822.1893	25.75	-822.2246	-822.1896	21.97	-3.78	-822.2252	-822.1923	20.66	-5.09

4.9 Reaction 9

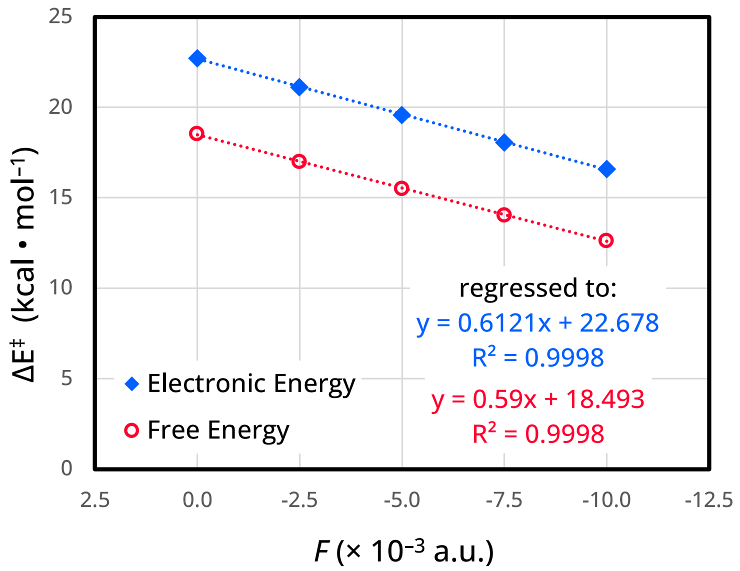

Figure S36. Effective activation energy ($\Delta E^{\ddagger}$ or $\Delta G^{\ddagger}$, kcal/mol) as a function of OEF magnitude for Reaction 9 computed at the B3LYP/def2-TZVP level of theory (gas phase, 298 K).

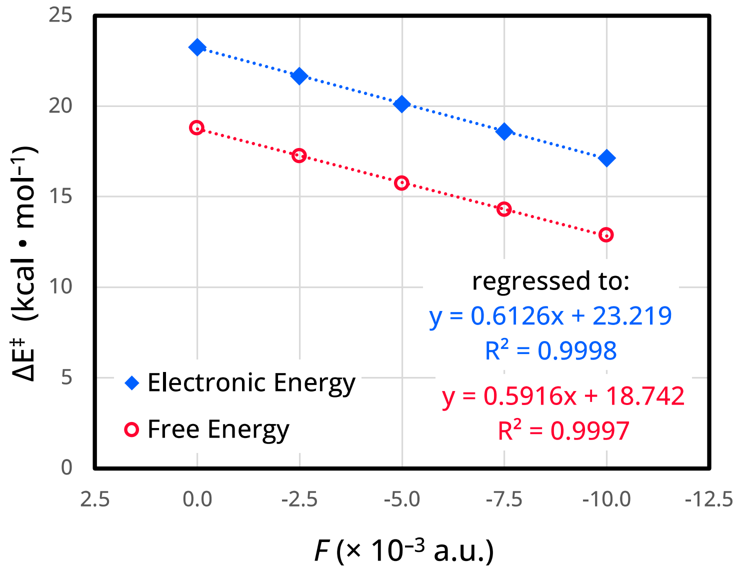

Figure S37. Effective activation energy ($\Delta E^{\ddagger}$ or $\Delta G^{\ddagger}$, kcal/mol) as a function of OEF magnitude for Reaction 9 computed at the B3LYP-D3/def2-TZVP level of theory (gas phase, 298 K).

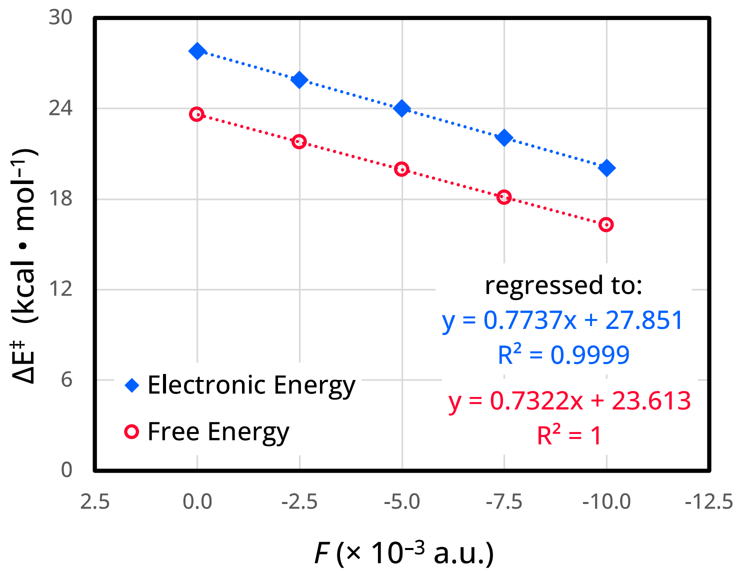

Figure S38. Effective activation energy ($\Delta E^{\ddagger}$ or $\Delta G^{\ddagger}$, kcal/mol) as a function of OEF magnitude for Reaction 9 computed at the M06-2X/def2-TZVP level of theory (gas phase, 298 K).

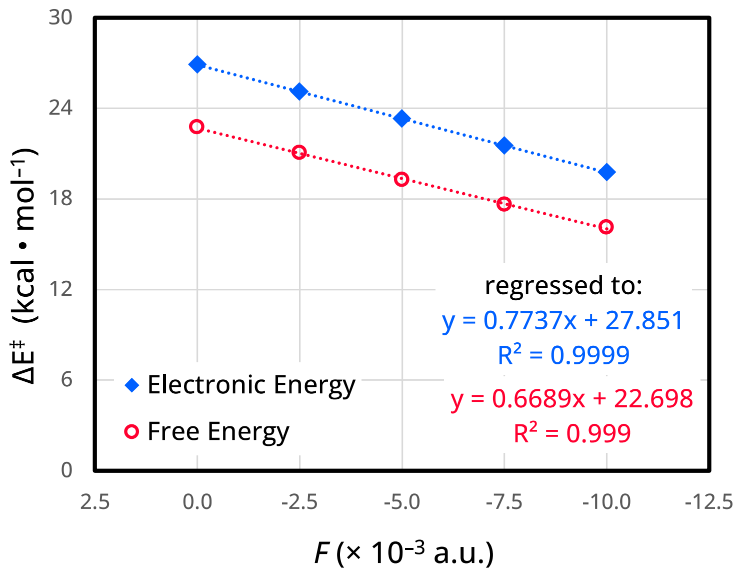

Figure S39. Effective activation energy ($\Delta E^{\ddagger}$ or $\Delta G^{\ddagger}$, kcal/mol) as a function of OEF magnitude for Reaction 9 computed at the ωB97X-D/def2-TZVP level of theory (gas phase, 298 K).

Table S50. Summary of A.V.E.D.A. output data for Reaction 9 computed at the B3LYP/def2-TZVP level of theory (gas phase, 298 K).

**Dipole Moments (debeye)**					
μ(Int) (x,y,z)	[0.0451, -0.9521, -4.3799]			||μ(Int)||	4.4824
μ(TS) (x,y,z)	[1.609, 0.0, -2.5427]			||μ(TS)||	3.0090
μ(rxn) (x,y,z)	[1.5639, 0.9521, 1.8372]			||μ(rxn)||	2.5938

**Oriented Electric Field**					
F [× 10^{-3} a.u.]	0	-2.5	-5	-7.5	-10
**Electronic Energies**					
Int [a.u.]	-289.0551	-289.0519	-289.0492	-289.0470	-289.0452
TS [a.u.]	-289.0189	-289.0182	-289.0180	-289.0182	-289.0187
$\Delta E^{\ddagger}$ [kcal/mol]	22.71	21.13	19.58	18.07	16.59
$\Delta\Delta E^{\ddagger}$ [kcal/mol]	0.00	-1.58	-3.13	-4.65	-6.12
**Free Energies**					
Int [a.u.]	-288.9325	-288.9294	-288.9269	-288.9248	-288.9231
TS [a.u.]	-288.9029	-288.9023	-288.9021	-288.9024	-288.9030
$\Delta G^{\ddagger}$ [kcal/mol]	18.53	17.00	15.51	14.05	12.63
$\Delta\Delta G^{\ddagger}$ [kcal/mol]	0.00	-1.53	-3.02	-4.49	-5.90
**RMSD from 0-Field**					
Int (Å)	-	0.0075	0.0147	0.0216	0.0286
TS (Å)	-	0.0069	0.0132	0.0193	0.0252

Table S51. Summary of A.V.E.D.A. output data for Reaction 9 computed at the B3LYP-D3/def2-TZVP level of theory (gas phase, 298 K).

**Dipole Moments 50ebeye)**					
μ(Int) (x,y,z)	[0.0251, -0.9745, -4.3716]			||μ(Int)||	4.4790
μ(TS) (x,y,z)	[1.5739, -0.0, -2.5413]			||μ(TS)||	2.9892
μ(rxn) (x,y,z)	[1.5488, 0.9745, 1.8303]			||μ(rxn)||	2.5881
**Oriented Electric Field**					
F [× 10^{-3} a.u.]	0	-2.5	-5	-7.5	-10
**Electronic Energies**					
Int [a.u.]	-289.0687	-289.0656	-289.0628	-289.0606	-289.0587
TS [a.u.]	-289.0317	-289.0310	-289.0308	-289.0309	-289.0314
ΔE‡ [kcal/mol]	23.25	21.67	20.12	18.61	17.13
ΔΔE‡ [kcal/mol]	0.00	-1.58	-3.13	-4.65	-6.13
**Free Energies**					
Int [a.u.]	-288.9455	-288.9424	-288.9398	-288.9377	-288.9361
TS [a.u.]	-288.9156	-288.9150	-288.9148	-288.9150	-288.9156
ΔG‡ [kcal/mol]	18.78	17.24	15.74	14.29	12.87
ΔΔG‡ [kcal/mol]	0.00	-1.54	-3.04	-4.50	-5.92
**RMSD from 0-Field**					
Int (Å)	-	0.0066	0.0125	0.0186	0.0246
TS (Å)	-	0.0071	0.0134	0.0192	0.0247

Table S52. Summary of A.V.E.D.A. output data for Reaction 9 computed at the M06-2X/def2-TZVP level of theory (gas phase, 298 K).

**Dipole Moments (debeye)**					
μ(Int) (x,y,z)	[0.075, -1.0442, -4.5117]			‖μ(Int)‖	4.6316
μ(TS) (x,y,z)	[2.2815, 0.0, -2.6207]			‖μ(TS)‖	3.4747
μ(rxn) (x,y,z)	[2.2065, 1.0442, 1.8910]			‖μ(rxn)‖	3.0879

**Oriented Electric Field**					
F [$\times 10^{-3}$ a.u.]	0	-2.5	-5	-7.5	-10
**Electronic Energies**					
Int [a.u.]	-288.9121	-288.9093	-288.9070	-288.9050	-288.9036
TS [a.u.]	-288.8678	-288.8680	-288.8687	-288.8699	-288.8716
$\Delta E^{\ddagger}$ [kcal/mol]	27.83	25.92	24.01	22.08	20.08
$\Delta\Delta E^{\ddagger}$ [kcal/mol]	0.00	-1.90	-3.81	-5.75	-7.75
**Free Energies**					
Int [a.u.]	-288.7870	-288.7842	-288.7820	-288.7802	-288.7788
TS [a.u.]	-288.7494	-288.7495	-288.7502	-288.7513	-288.7529
$\Delta G^{\ddagger}$ [kcal/mol]	23.60	21.79	19.97	18.12	16.28
$\Delta\Delta G^{\ddagger}$ [kcal/mol]	0.00	-1.82	-3.63	-5.48	-7.32
**RMSD from 0-Field**					
Int (Å)	-	0.0068	0.0116	0.0171	0.0227
TS (Å)	-	0.0279	0.0637	0.1100	0.1799

Table S53. Summary of A.V.E.D.A. output data for Reaction 9 computed at the ωB97X-D/def2-TZVP level of theory (gas phase, 298 K).

**Dipole Moments (debeye)**					
μ(Int) (x,y,z)	[0.0662, -1.0033, -4.4753]			||μ(Int)||	4.5869
μ(TS) (x,y,z)	[2.0505, -0.0003, -2.5417]			||μ(TS)||	3.2657
μ(rxn) (x,y,z)	[1.9843, 1.003, 1.9336]			||μ(rxn)||	2.9466

**Oriented Electric Field**					
F [× 10^{-3} a.u.]	0	-2.5	-5	-7.5	-10
**Electronic Energies**					
Int [a.u.]	-288.9608	-288.9579	-288.9554	-288.9533	-288.9516
TS [a.u.]	-288.9180	-288.9179	-288.9182	-288.9189	-288.9201
$\Delta E^{‡}$ [kcal/mol]	26.92	25.11	23.32	21.54	19.78
$\Delta\Delta E^{‡}$ [kcal/mol]	0.00	-1.81	-3.60	-5.37	-7.14
**Free Energies**					
Int [a.u.]	-288.8360	-288.8332	-288.8307	-288.8288	-288.8276
TS [a.u.]	-288.7998	-288.7997	-288.8000	-288.8008	-288.8020
$\Delta G^{‡}$ [kcal/mol]	22.75	21.04	19.25	17.61	16.11
$\Delta\Delta G^{‡}$ [kcal/mol]	0.00	-1.71	-3.50	-5.14	-6.65
**RMSD from 0-Field**					
Int (Å)	-	0.0074	0.0132	0.0168	0.0265
TS (Å)	-	0.0163	0.0342	0.0554	0.0814

Table S54. Summary of A.V.E.D.A. output data for Reaction 9 computed in the gas phase at 298 K.

Level of Theory	F = 0 a.u.			F = 2.5 10^{-3} a.u.				F = 5.0 10^{-3} a.u.			
	E_{Int}	E_{TS}	$\Delta E^{‡}$	E_{Int}	E_{TS}	$\Delta E^{‡}$	$\Delta\Delta E^{‡}$	E_{Int}	E_{TS}	$\Delta E^{‡}$	$\Delta\Delta E^{‡}$
B3LYP/def2-TZVP											
Electronic Energy	-289.0551	-289.0189	22.72	-289.0519	-289.0182	21.13	-1.59	-289.0492	-289.0180	19.58	-3.13
Enthalpy	-288.8936	-288.8642	18.47	-288.8906	-288.8636	16.94	-1.53	-288.8880	-288.8634	15.45	-3.02
Free Energy	-288.9325	-288.9029	18.53	-288.9294	-288.9023	17.00	-1.53	-288.9269	-288.9021	15.51	-3.02
B3LYP-D3/def2-TZVP											
Electronic Energy	-289.0687	-289.0317	23.25	-289.0656	-289.0310	21.67	-1.58	-289.0628	-289.0308	20.12	-3.13
Enthalpy	-288.9070	-288.8769	18.83	-288.9039	-288.8763	17.30	-1.53	-288.9013	-288.8761	15.81	-3.03
Free Energy	-288.9455	-288.9156	18.78	-288.9424	-288.9150	17.24	-1.54	-288.9398	-288.9148	15.74	-3.04
M06-2X/def2-TZVP											
Electronic Energy	-288.9121	-288.8678	27.83	-288.9093	-288.8680	25.92	-1.90	-288.9070	-288.8687	24.01	-3.81
Enthalpy	-288.7487	-288.7110	23.63	-288.7459	-288.7112	21.79	-1.84	-288.7437	-288.7119	19.96	-3.67
Free Energy	-288.7870	-288.7494	23.60	-288.7842	-288.7495	21.79	-1.82	-288.7820	-288.7502	19.97	-3.63
ωB97X-D/def2-TZVP											
Electronic Energy	-288.9608	-288.9180	26.92	-288.9579	-288.9179	25.11	-1.81	-288.9554	-288.9182	23.32	-3.60
Enthalpy	-288.7976	-288.7614	22.70	-288.7948	-288.7614	20.97	-1.74	-288.7923	-288.7617	19.21	-3.49
Free Energy	-288.8360	-288.7998	22.75	-288.8332	-288.7997	21.04	-1.71	-288.8307	-288.8000	19.25	-3.50

Table S55. Summary of A.V.E.D.A. output data for Reaction 9 computed in the gas phase at 298 K.

Level of Theory	F = 0 a.u.			F = 7.5 10^{-3} a.u.				F = 10.0 10^{-3} a.u.			
	E_{Int}	E_{TS}	$\Delta E^‡$	E_{Int}	E_{TS}	$\Delta E^‡$	$\Delta\Delta E^‡$	E_{Int}	E_{TS}	$\Delta E^‡$	$\Delta\Delta E^‡$
B3LYP/def2-TZVP											
Electronic Energy	-289.0551	-289.0189	22.72	-289.0470	-289.0182	18.07	-4.65	-289.0452	-289.0187	16.59	-6.12
Enthalpy	-288.8936	-288.8642	18.47	-288.8859	-288.8636	13.99	-4.48	-288.8843	-288.8642	12.58	-5.90
Free Energy	-288.9325	-288.9029	18.53	-288.9248	-288.9024	14.05	-4.49	-288.9231	-288.9030	12.63	-5.90
B3LYP-D3/def2-TZVP											
Electronic Energy	-289.0687	-289.0317	23.25	-289.0606	-289.0309	18.61	-4.65	-289.0587	-289.0314	17.13	-6.13
Enthalpy	-288.9070	-288.8769	18.83	-288.8992	-288.8763	14.35	-4.49	-288.8975	-288.8769	12.92	-5.91
Free Energy	-288.9455	-288.9156	18.78	-288.9377	-288.9150	14.29	-4.50	-288.9361	-288.9156	12.87	-5.92
M06-2X/def2-TZVP											
Electronic Energy	-288.9121	-288.8678	27.83	-288.9050	-288.8699	22.08	-5.75	-288.9036	-288.8716	20.08	-7.75
Enthalpy	-288.7487	-288.7110	23.63	-288.7419	-288.7131	18.10	-5.53	-288.7405	-288.7146	16.25	-7.38
Free Energy	-288.7870	-288.7494	23.60	-288.7802	-288.7513	18.12	-5.48	-288.7788	-288.7529	16.28	-7.32
ωB97X-D/def2-TZVP											
Electronic Energy	-288.9608	-288.9180	26.92	-288.9533	-288.9189	21.55	-5.37	-288.9516	-288.9201	19.78	-7.14
Enthalpy	-288.7976	-288.7614	22.70	-288.7904	-288.7625	17.54	-5.17	-288.7890	-288.7637	15.91	-6.79
Free Energy	-288.8360	-288.7998	22.75	-288.8288	-288.8008	17.61	-5.14	-288.8276	-288.8020	16.11	-6.65

4.10 Reaction 10

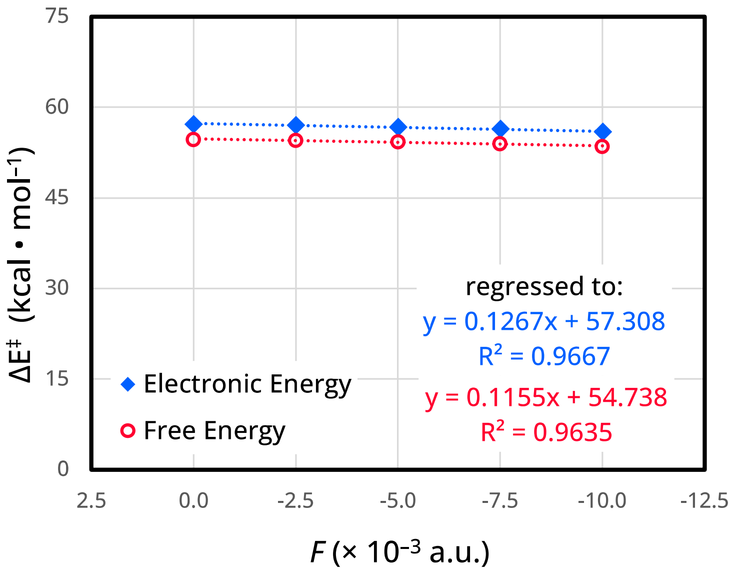

Figure S40. Effective activation energy ($\Delta E^{\ddagger}$ or $\Delta G^{\ddagger}$, kcal/mol) as a function of OEF magnitude for Reaction 10 computed at the B3LYP/def2-TZVP level of theory (gas phase, 298 K).

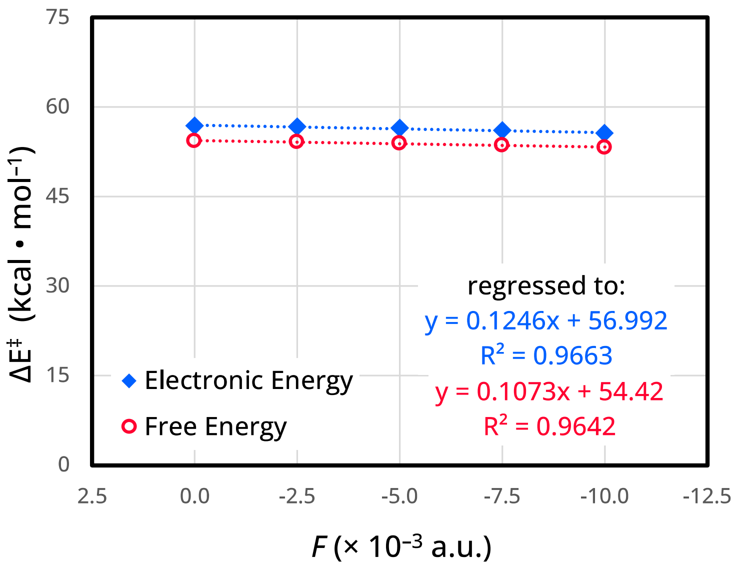

Figure S41. Effective activation energy ($\Delta E^{\ddagger}$ or $\Delta G^{\ddagger}$, kcal/mol) as a function of OEF magnitude for Reaction 10 computed at the B3LYP-D3/def2-TZVP level of theory (gas phase, 298 K).

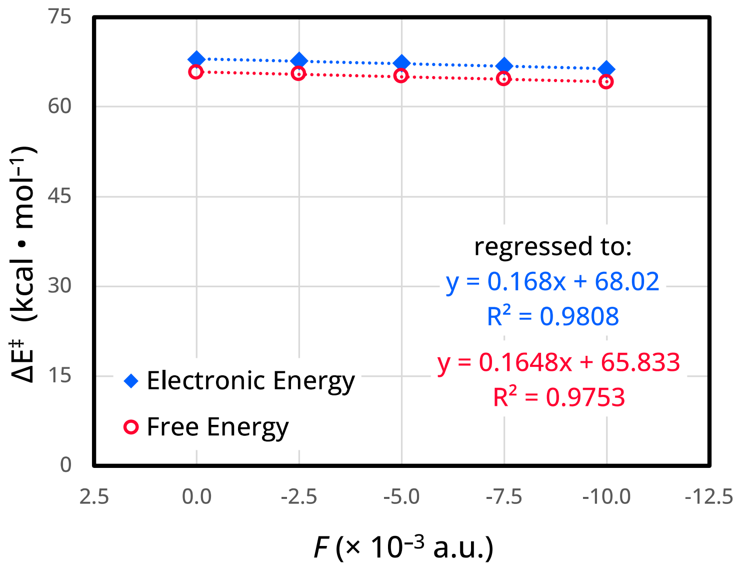

Figure S42. Effective activation energy ($\Delta E^{\ddagger}$ or $\Delta G^{\ddagger}$, kcal/mol) as a function of OEF magnitude for Reaction 10 computed at the M06-2X/def2-TZVP level of theory (gas phase, 298 K).

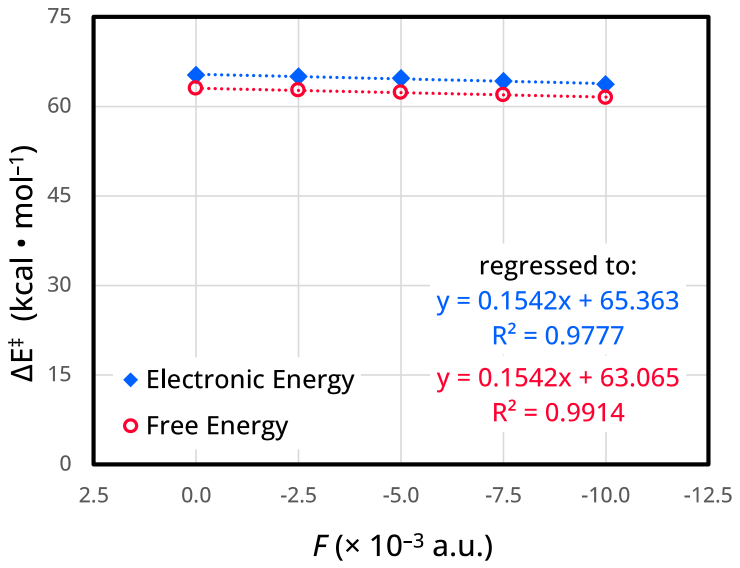

Figure S43. Effective activation energy ($\Delta E^{\ddagger}$ or $\Delta G^{\ddagger}$, kcal/mol) as a function of OEF magnitude for Reaction 10 computed at the ωB97X-D/def2-TZVP level of theory (gas phase, 298 K).

Table S56. Summary of A.V.E.D.A. output data for Reaction 10 computed at the B3LYP/def2-TZVP level of theory (gas phase, 298 K).

**Dipole Moments (debeye)**					
μ(Int) (x,y,z)	[-0.3685, -0.2279, -0.0824]			‖μ(Int)‖	0.4410
μ(TS) (x,y,z)	[-0.5128, -0.3318, -0.0067]			‖μ(TS)‖	0.6108
μ(rxn) (x,y,z)	[-0.1443, -0.1039, 0.0757]			‖μ(rxn)‖	0.1933

**Oriented Electric Field**					
F [× 10^{-3} a.u.]	0	-2.5	-5	-7.5	-10
**Electronic Energies**					
Int [a.u.]	-195.3673	-195.3679	-195.3689	-195.3704	-195.3723
TS [a.u.]	-195.2761	-195.2770	-195.2784	-195.2805	-195.2831
$\Delta E^{\ddagger}$ [kcal/mol]	57.21	57.04	56.77	56.41	55.94
$\Delta\Delta E^{\ddagger}$ [kcal/mol]	0.00	-0.17	-0.44	-0.80	-1.27
**Free Energies**					
Int [a.u.]	-195.2812	-195.2818	-195.2829	-195.2845	-195.2866
TS [a.u.]	-195.1942	-195.1950	-195.1965	-195.1986	-195.2013
$\Delta G^{\ddagger}$ [kcal/mol]	54.64	54.50	54.26	53.92	53.49
$\Delta\Delta G^{\ddagger}$ [kcal/mol]	0.00	-0.15	-0.39	-0.72	-1.15
**RMSD from 0-Field**					
Int (Å)	-	0.0151986	0.01815716	0.02956882	0.0425135
TS (Å)	-	0.00402076	0.00934822	0.01632603	0.02514413

Table S57. Summary of A.V.E.D.A. output data for Reaction 10 computed at the B3LYP-D3/def2-TZVP level of theory (gas phase, 298 K).

**Dipole Moments (debeye)**					
μ(Int) (x,y,z)	[-0.3704, -0.2298, -0.0792]			||μ(Int)||	0.4430
μ(TS) (x,y,z)	[-0.5053, -0.3344, -0.0043]			||μ(TS)||	0.6059
μ(rxn) (x,y,z)	[-0.1349, -0.1046, 0.07490]			||μ(rxn)||	0.1864

**Oriented Electric Field**					
F [× 10^{-3} a.u.]	0	-2.5	-5	-7.5	-10
**Electronic Energies**					
Int [a.u.]	-195.3738	-195.3743	-195.3753	-195.3768	-195.3787
TS [a.u.]	-195.2831	-195.2839	-195.2854	-195.2874	-195.2900
ΔE‡ [kcal/mol]	56.89	56.73	56.47	56.11	55.65
ΔΔE‡ [kcal/mol]	0.00	-0.16	-0.43	-0.79	-1.25
**Free Energies**					
Int [a.u.]	-195.2876	-195.2882	-195.2893	-195.2909	-195.2930
TS [a.u.]	-195.2010	-195.2018	-195.2033	-195.2054	-195.2081
ΔG‡ [kcal/mol]	54.33	54.20	53.97	53.66	53.26
ΔΔG‡ [kcal/mol]	0.00	-0.14	-0.36	-0.68	-1.07
**RMSD from 0-Field**					
Int (Å)	-	0.0094	0.0208	0.0339	0.0497
TS (Å)	-	0.00396	0.0089	0.0156	0.0240

Table S58. Summary of A.V.E.D.A. output data for Reaction 10 computed at the M06-2X/def2-TZVP level of theory (gas phase, 298 K).

**Dipole Moments (debeye)**					
μ(Int) (x,y,z)	[-0.3482, -0.1985, -0.0708]			‖μ(Int)‖	0.4070
μ(TS) (x,y,z)	[-0.708, -0.1529, -0.0331]			‖μ(TS)‖	0.7251
μ(rxn) (x,y,z)	[-0.3598, 0.0456, 0.0377]			‖μ(rxn)‖	0.3646

**Oriented Electric Field**					
F [× 10^{-3} a.u.]	0	-2.5	-5	-7.5	-10
**Electronic Energies**					
Int [a.u.]	-195.2667	-195.2672	-195.2680	-195.2693	-195.2709
TS [a.u.]	-195.1584	-195.1593	-195.1608	-195.1628	-195.1654
$\Delta E^{\ddagger}$ [kcal/mol]	67.92	67.65	67.28	66.81	66.24
$\Delta\Delta E^{\ddagger}$ [kcal/mol]	0.00	-0.27	-0.64	-1.11	-1.68
**Free Energies**					
Int [a.u.]	-195.1793	-195.1798	-195.1807	-195.1820	-195.1838
TS [a.u.]	-195.0745	-195.0754	-195.0769	-195.0790	-195.0817
$\Delta G^{\ddagger}$ [kcal/mol]	65.72	65.48	65.12	64.64	64.08
$\Delta\Delta G^{\ddagger}$ [kcal/mol]	0.00	-0.24	-0.60	-1.08	-1.64
**RMSD from 0-Field**					
Int (Å)	-	1.2242	1.2233	1.2230	1.2221
TS (Å)	-	1.1399	1.1399	1.1402	1.1407

Table S59. Summary of A.V.E.D.A. output data for Reaction 10 computed at the ωB97X-D /def2-TZVP level of theory (gas phase, 298 K).

**Dipole Moments (debeye)**					
μ(Int) (x,y,z)	[-0.36, -0.2167, -0.0756]			‖μ(Int)‖	0.4269
μ(TS) (x,y,z)	[-0.672, -0.2172, -0.0249]			‖μ(TS)‖	0.7067
μ(rxn) (x,y,z)	[-0.3120, -0.0005, 0.0507]			‖μ(rxn)‖	0.3161

**Oriented Electric Field**					
F [× 10^{-3} a.u.]	0	-2.5	-5	-7.5	-10
**Electronic Energies**					
Int [a.u.]	-195.2968	-195.2973	-195.2982	-195.2995	-195.3013
TS [a.u.]	-195.1928	-195.1937	-195.1951	-195.1971	-195.1997
$\Delta E^{\ddagger}$ [kcal/mol]	65.27	65.02	64.69	64.26	63.72
$\Delta\Delta E^{\ddagger}$ [kcal/mol]	0.00	-0.24	-0.57	-1.01	-1.54
**Free Energies**					
Int [a.u.]	-195.2096	-195.2101	-195.2111	-195.2125	-195.2143
TS [a.u.]	-195.1092	-195.1102	-195.1117	-195.1138	-195.1164
$\Delta G^{\ddagger}$ [kcal/mol]	63.00	62.71	62.36	61.94	61.46
$\Delta\Delta G^{\ddagger}$ [kcal/mol]	0.00	-0.30	-0.65	-1.07	-1.54
**RMSD from 0-Field**					
Int (Å)	-	1.2290	1.2283	1.2280	1.2269
TS (Å)	-	1.1391	1.1392	1.1394	1.1397

Table S60. Summary of A.V.E.D.A. output data for Reaction 10 computed in the gas phase at 298 K.

Level of Theory	F = 0 a.u.			F = 2.5 10^{-3} a.u.				F = 5.0 10^{-3} a.u.			
	E_{Int}	E_{TS}	$\Delta E^{\ddagger}$	E_{Int}	E_{TS}	$\Delta E^{\ddagger}$	$\Delta\Delta E^{\ddagger}$	E_{Int}	E_{TS}	$\Delta E^{\ddagger}$	$\Delta\Delta E^{\ddagger}$
B3LYP/def2-TZVP											
Electronic Energy	-195.3673	-195.2761	57.21	-195.3679	-195.2770	57.04	-0.17	-195.3689	-195.2784	56.77	-0.44
Enthalpy	-195.2466	-195.1601	54.29	-195.2472	-195.1609	54.12	-0.17	-195.2482	-195.1624	53.86	-0.43
Free Energy	-195.2812	-195.1942	54.64	-195.2818	-195.1950	54.50	-0.15	-195.2829	-195.1965	54.26	-0.39
B3LYP-D3/def2-TZVP											
Electronic Energy	-195.3738	-195.2831	56.90	-195.3743	-195.2839	56.73	-0.16	-195.3753	-195.2854	56.47	-0.43
Enthalpy	-195.2530	-195.1670	53.98	-195.2536	-195.1678	53.82	-0.16	-195.2546	-195.1693	53.56	-0.42
Free Energy	-195.2876	-195.2010	54.33	-195.2882	-195.2018	54.20	-0.14	-195.2893	-195.2033	53.97	-0.36
M06-2X/def2-TZVP											
Electronic Energy	-195.2667	-195.1584	67.92	-195.2672	-195.1593	67.65	-0.27	-195.2680	-195.1608	67.28	-0.64
Enthalpy	-195.1447	-195.0408	65.23	-195.1452	-195.0417	64.96	-0.26	-195.1461	-195.0432	64.59	-0.64
Free Energy	-195.1793	-195.0745	65.72	-195.1798	-195.0754	65.48	-0.24	-195.1807	-195.0769	65.12	-0.60
ωB97X-D/def2-TZVP											
Electronic Energy	-195.2968	-195.1928	65.27	-195.2973	-195.1937	65.03	-0.24	-195.2982	-195.1951	64.69	-0.57
Enthalpy	-195.1750	-195.0754	62.54	-195.1756	-195.0764	62.24	-0.29	-195.1765	-195.0779	61.88	-0.65
Free Energy	-195.2096	-195.1092	63.01	-195.2101	-195.1102	62.71	-0.30	-195.2111	-195.1117	62.36	-0.65

Table S61. Summary of A.V.E.D.A. output data for Reaction 10 computed in the gas phase at 298 K, continued

Level of Theory	F = 0 a.u.			F = 7.5 10^{-3} a.u.				F = 10.0 10^{-3} a.u.			
	E_{Int}	E_{TS}	$\Delta E^{‡}$	E_{Int}	E_{TS}	$\Delta E^{‡}$	$\Delta\Delta E^{‡}$	E_{Int}	E_{TS}	$\Delta E^{‡}$	$\Delta\Delta E^{‡}$
B3LYP/def2-TZVP											
Electronic Energy	-195.3673	-195.2761	57.21	-195.3704	-195.2805	56.41	-0.80	-195.3720	-195.2824	56.28	-0.93
Enthalpy	-195.2466	-195.1601	54.29	-195.2497	-195.1645	53.50	-0.79	-195.2515	-195.1665	53.33	-0.96
Free Energy	-195.2812	-195.1942	54.64	-195.2845	-195.1986	53.92	-0.72	-195.2862	-195.2006	53.77	-0.88
B3LYP-D3/def2-TZVP											
Electronic Energy	-195.3738	-195.2831	56.90	-195.3768	-195.2874	56.11	-0.79	-195.3787	-195.2900	55.65	-1.25
Enthalpy	-195.2530	-195.1670	53.98	-195.2561	-195.1714	53.20	-0.77	-195.2581	-195.1741	52.75	-1.23
Free Energy	-195.2876	-195.2010	54.33	-195.2909	-195.2054	53.66	-0.68	-195.2930	-195.2081	53.26	-1.07
M06-2X/def2-TZVP											
Electronic Energy	-195.2667	-195.1584	67.92	-195.2693	-195.1628	66.81	-1.11	-195.2709	-195.1654	66.24	-1.68
Enthalpy	-195.1447	-195.0408	65.23	-195.1474	-195.0453	64.10	-1.12	-195.1491	-195.0479	63.52	-1.71
Free Energy	-195.1793	-195.0745	65.72	-195.1820	-195.0790	64.64	-1.08	-195.1838	-195.0817	64.08	-1.64
ωB97X-D/def2-TZVP											
Electronic Energy	-195.2968	-195.1928	65.27	-195.2995	-195.1971	64.26	-1.01	-195.3013	-195.1997	63.72	-1.54
Enthalpy	-195.1750	-195.0754	62.54	-195.1779	-195.0799	61.45	-1.09	-195.1796	-195.0825	60.94	-1.59
Free Energy	-195.2096	-195.1092	63.01	-195.2125	-195.1138	61.94	-1.07	-195.2143	-195.1164	61.46	-1.54

4.11 Reaction 11

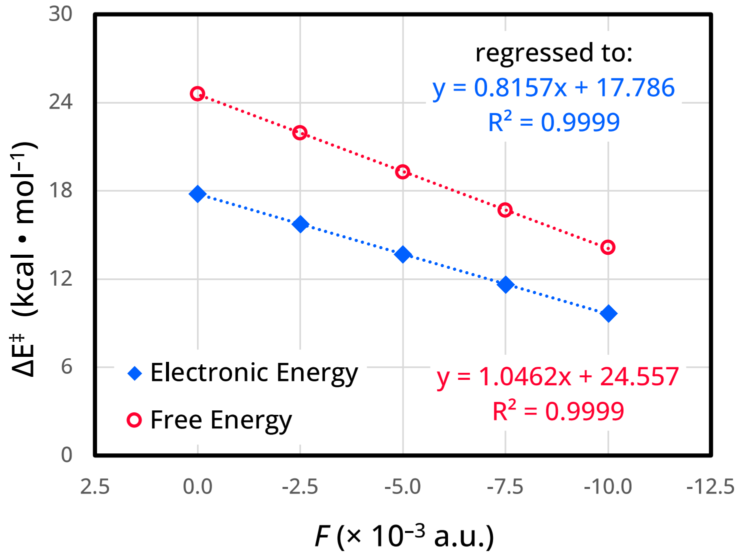

Figure S44. Effective activation energy ($\Delta E^{\ddagger}$ or $\Delta G^{\ddagger}$, kcal/mol) as a function of OEF magnitude for Reaction 11 computed at the B3LYP/def2-TZVP level of theory (gas phase, 298 K).

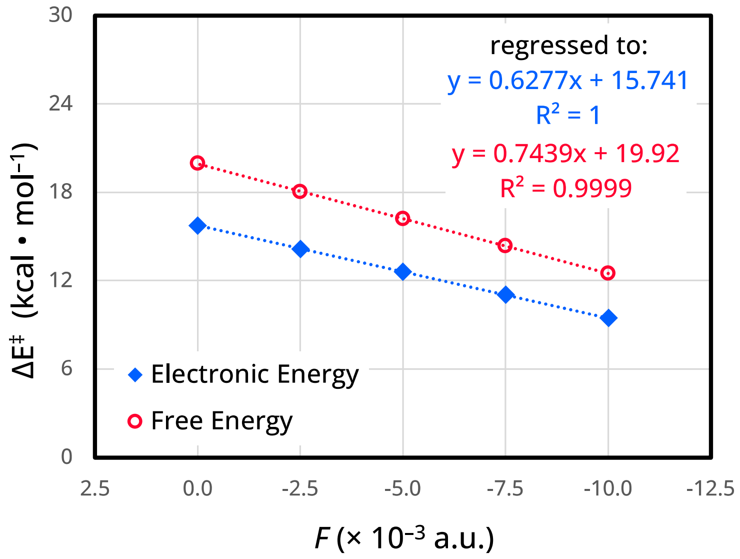

Figure S45. Effective activation energy ($\Delta E^{\ddagger}$ or $\Delta G^{\ddagger}$, kcal/mol) as a function of OEF magnitude for Reaction 11 computed at the B3LYP-D3/def2-TZVP level of theory (gas phase, 298 K).

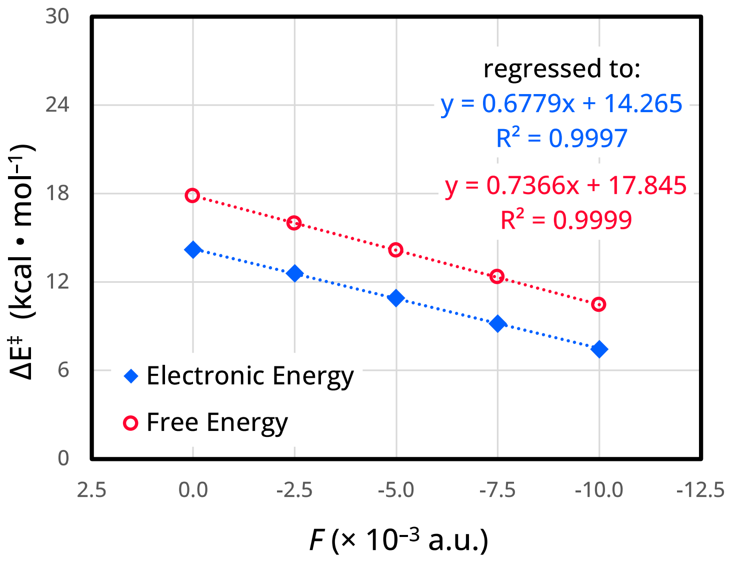

Figure S46. Effective activation energy (Δ*E*‡ or Δ*G*‡, kcal/mol) as a function of OEF magnitude for Reaction 11 computed at the M06-2X/def2-TZVP level of theory (gas phase, 298 K).

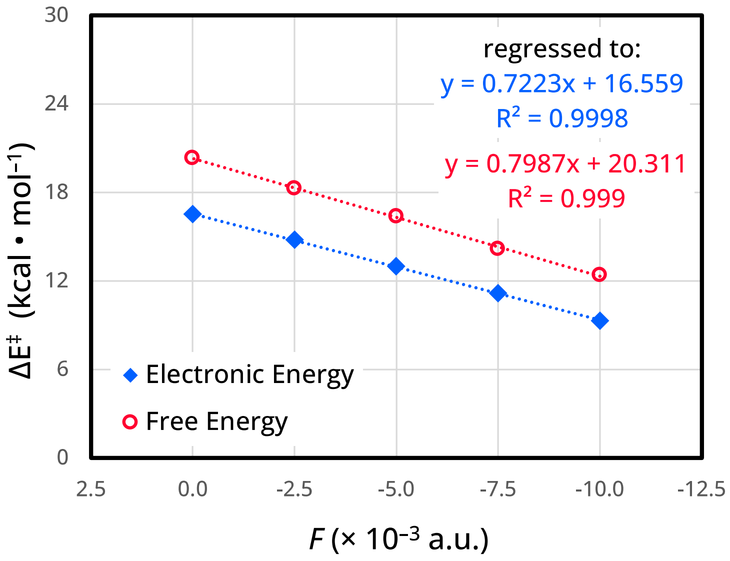

Figure S47. Effective activation energy (Δ*E*‡ or Δ*G*‡, kcal/mol) as a function of OEF magnitude for Reaction 11 computed at the ωB97X-D/def2-TZVP level of theory (gas phase, 298 K).

Table S62. Summary of A.V.E.D.A. output data for Reaction 11 computed at the B3LYP/def2-TZVP level of theory (gas phase, 298 K).

**Dipole Moments (debeye)**					
μ(Int) (x,y,z)	[-3.6431, 2.8032, 0.1641]			||μ(Int)||	4.5997
μ(TS) (x,y,z)	[-2.1473, 5.7273, -0.0]			||μ(TS)||	6.1166
μ(rxn) (x,y,z)	[1.4958, 2.9241, -0.1641]			||μ(rxn)||	3.2886

**Oriented Electric Field**					
F [× 10^{-3} a.u.]	0	-2.5	-5	-7.5	-10
**Electronic Energies**					
Int [a.u.]	-573.6288	-573.6301	-573.6323	-573.6355	-573.6398
TS [a.u.]	-573.6005	-573.6050	-573.6105	-573.6169	-573.6243
$\Delta E^{\ddagger}$ [kcal/mol]	17.81	15.75	13.68	11.63	9.67
$\Delta\Delta E^{\ddagger}$ [kcal/mol]	0.00	-2.05	-4.13	-6.17	-8.14
**Free Energies**					
Int [a.u.]	-573.5234	-573.5238	-573.5251	-573.5276	-573.5312
TS [a.u.]	-573.4842	-573.4888	-573.4944	-573.5010	-573.5087
$\Delta G^{\ddagger}$ [kcal/mol]	24.60	21.94	19.27	16.67	14.15
$\Delta\Delta G^{\ddagger}$ [kcal/mol]	0.00	-2.66	-5.32	-7.93	-10.44
**RMSD from 0-Field**					
Int (Å)	-	0.0757	0.0961	0.1135	0.1391
TS (Å)	-	0.0291	0.0561	0.0816	0.1054

Table S63. Summary of A.V.E.D.A. output data for Reaction 11 computed at the B3LYP-D3/def2-TZVP level of theory (gas phase, 298 K).

**Dipole Moments (debeye)**					
μ(Int) (x,y,z)	[-3.3167, 3.3677, -0.0342]			‖μ(Int)‖	4.7268
μ(TS) (x,y,z)	[-2.1654, 5.6484, -0.0]			‖μ(TS)‖	6.0492
μ(rxn) (x,y,z)	[1.1513, 2.2807, 0.0342]			‖μ(rxn)‖	2.5550
**Oriented Electric Field**					
F [× 10^{-3} a.u.]	0	-2.5	-5	-7.5	-10
**Electronic Energies**					
Int [a.u.]	-573.6462	-573.6481	-573.6510	-573.6548	-573.6597
TS [a.u.]	-573.6211	-573.6256	-573.6309	-573.6372	-573.6446
$\Delta E^{\ddagger}$ [kcal/mol]	15.74	14.17	12.60	11.05	9.46
$\Delta\Delta E^{\ddagger}$ [kcal/mol]	0.00	-1.57	-3.14	-4.70	-6.28
**Free Energies**					
Int [a.u.]	-573.5364	-573.5378	-573.5404	-573.5440	-573.5486
TS [a.u.]	-573.5046	-573.5091	-573.5146	-573.5211	-573.5287
$\Delta G^{\ddagger}$ [kcal/mol]	19.95	18.02	16.20	14.36	12.48
$\Delta\Delta G^{\ddagger}$ [kcal/mol]	0.00	-1.93	-3.74	-5.59	-7.47
**RMSD from 0-Field**					
Int (Å)	-	1.8762	1.8745	1.8734	1.8731
TS (Å)	-	1.7415	1.7450	1.7499	1.7571

Table S64. Summary of A.V.E.D.A. output data for Reaction 11 computed at the M06-2X/def2-TZVP level of theory (gas phase, 298 K).

**Dipole Moments (debeye)**					
μ(Int) (x,y,z)	[-3.3713, 3.2195, -0.049]			||μ(Int)||	4.6619
μ(TS) (x,y,z)	[-2.2422, 5.5205, -0.0]			||μ(TS)||	5.9585
μ(rxn) (x,y,z)	[1.1291, 2.301, 0.049]			||μ(rxn)||	2.5636

**Oriented Electric Field**					
F [× 10^{-3} a.u.]	0	-2.5	-5	-7.5	-10
**Electronic Energies**					
Int [a.u.]	-573.3954	-573.3972	-573.3998	-573.4033	-573.4077
TS [a.u.]	-573.3727	-573.3771	-573.3824	-573.3886	-573.3958
$\Delta E^{\ddagger}$ [kcal/mol]	14.21	12.60	10.92	9.19	7.44
$\Delta\Delta E^{\ddagger}$ [kcal/mol]	0.00	-1.61	-3.29	-5.02	-6.77
**Free Energies**					
Int [a.u.]	-573.2824	-573.2839	-573.2863	-573.2898	-573.2940
TS [a.u.]	-573.2540	-573.2584	-573.2638	-573.2701	-573.2773
$\Delta G^{\ddagger}$ [kcal/mol]	17.85	15.98	14.17	12.36	10.45
$\Delta\Delta G^{\ddagger}$ [kcal/mol]	0.00	-1.87	-3.68	-5.49	-7.40
**RMSD from 0-Field**					
Int (Å)	-	1.8601	1.8613	1.8618	1.8621
TS (Å)	-	1.8383	1.8367	1.8352	1.8339

Table S65. Summary of A.V.E.D.A. output data for Reaction 11 computed at the ωB97X-D/def2-TZVP level of theory (gas phase, 298 K).

**Dipole Moments (debeye)**					
μ(Int) (x,y,z)	[-3.4148, 3.0922, -0.008]			||μ(Int)||	4.6068
μ(TS) (x,y,z)	[-2.1961, 5.5615, -0.0]			||μ(TS)||	5.9794
μ(rxn) (x,y,z)	[1.2187, 2.4693, 0.008]			||μ(rxn)||	2.7537

**Oriented Electric Field**					
F [× 10^{-3} a.u.]	0	-2.5	-5	-7.5	-10
**Electronic Energies**					
Int [a.u.]	-573.4345	-573.4362	-573.4387	-573.4420	-573.4463
TS [a.u.]	-573.4082	-573.4126	-573.4180	-573.4243	-573.4315
ΔE‡ [kcal/mol]	16.52	14.78	12.99	11.15	9.30
ΔΔE‡ [kcal/mol]	0.00	-1.74	-3.53	-5.36	-7.22
**Free Energies**					
Int [a.u.]	-573.3224	-573.3235	-573.3260	-573.3289	-573.3335
TS [a.u.]	-573.2900	-573.2944	-573.2999	-573.3063	-573.3137
ΔG‡ [kcal/mol]	20.34	18.27	16.39	14.17	12.41
ΔΔG‡ [kcal/mol]	0.00	-2.07	-3.95	-6.18	-7.93
**RMSD from 0-Field**					
Int (Å)	-	1.8855	1.8846	1.8831	1.8826
TS (Å)	-	1.8346	1.8329	1.8315	1.8302

Table S66. Summary of A.V.E.D.A. output data for Reaction 11 computed in the gas phase at 298 K.

Level of Theory	F = 0 a.u.			F = 2.5 10^{-3} a.u.				F = 5.0 10^{-3} a.u.			
	E_{Int}	E_{TS}	$\Delta E^{‡}$	E_{Int}	E_{TS}	$\Delta E^{‡}$	$\Delta\Delta E^{‡}$	E_{Int}	E_{TS}	$\Delta E^{‡}$	$\Delta\Delta E^{‡}$
B3LYP/def2-TZVP											
Electronic Energy	-573.6288	-573.6005	17.81	-573.6301	-573.6050	15.75	-2.06	-573.6323	-573.6105	13.68	-4.13
Enthalpy	-573.4677	-573.4395	17.69	-573.4690	-573.4440	15.66	-2.03	-573.4712	-573.4495	13.61	-4.09
Free Energy	-573.5234	-573.4842	24.60	-573.6301	-573.6050	15.75	-8.85	-573.6323	-573.6105	13.68	-10.92
B3LYP-D3/def2-TZVP											
Electronic Energy	-573.6462	-573.6211	15.74	-573.6481	-573.6256	14.17	-1.57	-573.6510	-573.6309	12.60	-3.14
Enthalpy	-573.4850	-573.4601	15.63	-573.4869	-573.4645	14.06	-1.57	-573.4898	-573.4699	12.50	-3.13
Free Energy	-573.5364	-573.5046	19.95	-573.5378	-573.5091	18.02	-1.93	-573.5404	-573.5146	16.20	-3.74
M06-2X/def2-TZVP											
Electronic Energy	-573.3954	-573.3727	14.21	-573.3972	-573.3771	12.60	-1.61	-573.3998	-573.3824	10.92	-3.29
Enthalpy	-573.2322	-573.2099	14.03	-573.2340	-573.2142	12.43	-1.61	-573.2367	-573.2195	10.76	-3.27
Free Energy	-573.2824	-573.2540	17.85	-573.2839	-573.2584	15.98	-1.87	-573.2863	-573.2638	14.17	-3.68
ωB97X-D/def2-TZVP											
Electronic Energy	-573.4345	-573.4082	16.52	-573.4362	-573.4126	14.78	-1.74	-573.4387	-573.4180	12.99	-3.53
Enthalpy	-573.2714	-573.2455	16.28	-573.2731	-573.2498	14.59	-1.68	-573.2756	-573.2552	12.82	-3.46
Free Energy	-573.3224	-573.2900	20.34	-573.3235	-573.2944	18.27	-2.07	-573.3260	-573.2999	16.39	-3.95

Table S67. Summary of A.V.E.D.A. output data for Reaction 11 computed in the gas phase at 298 K, continued.

Level of Theory	F = 0 a.u.			F = 7.5 10^{-3} a.u.				F = 5.0 10^{-3} a.u.			
	E_{Int}	E_{TS}	$\Delta E^{\ddagger}$	E_{Int}	E_{TS}	$\Delta E^{\ddagger}$	$\Delta\Delta E^{\ddagger}$	E_{Int}	E_{TS}	$\Delta E^{\ddagger}$	$\Delta\Delta E^{\ddagger}$
B3LYP/def2-TZVP											
Electronic Energy	-573.6288	-573.6005	17.81	-573.6355	-573.6169	11.63	-6.17	-573.6398	-573.6243	9.67	-8.14
Enthalpy	-573.4677	-573.4395	17.69	-573.4744	-573.4560	11.58	-6.12	-573.4788	-573.4634	9.63	-8.06
Free Energy	-573.5234	-573.4842	24.60	-573.6355	-573.6169	11.63	-12.96	-573.5312	-573.5087	14.15	-10.44
B3LYP-D3/def2-TZVP											
Electronic Energy	-573.6462	-573.6211	15.74	-573.6548	-573.6372	11.05	-4.70	-573.6597	-573.6446	9.46	-6.29
Enthalpy	-573.4850	-573.4601	15.63	-573.4937	-573.4763	10.95	-4.68	-573.4986	-573.4837	9.36	-6.27
Free Energy	-573.5364	-573.5046	19.95	-573.5440	-573.5211	14.36	-5.59	-573.5486	-573.5287	12.48	-7.47
M06-2X/def2-TZVP											
Electronic Energy	-573.3954	-573.3727	14.21	-573.4033	-573.3886	9.20	-5.02	-573.4077	-573.3958	7.44	-6.77
Enthalpy	-573.2322	-573.2099	14.03	-573.2402	-573.2258	9.06	-4.97	-573.2447	-573.2330	7.32	-6.72
Free Energy	-573.2824	-573.2540	17.85	-573.2898	-573.2701	12.37	-5.49	-573.2940	-573.2773	10.45	-7.40
ωB97X-D/def2-TZVP											
Electronic Energy	-573.4345	-573.4082	16.52	-573.4420	-573.4243	11.15	-5.36	-573.4463	-573.4315	9.30	-7.22
Enthalpy	-573.2714	-573.2455	16.28	-573.2790	-573.2615	10.94	-5.34	-573.2834	-573.2688	9.16	-7.12
Free Energy	-573.3224	-573.2900	20.34	-573.3289	-573.3063	14.17	-6.18	-573.3335	-573.3137	12.41	-7.93

4.12 Reaction 12 (Acetylene Dibromination)

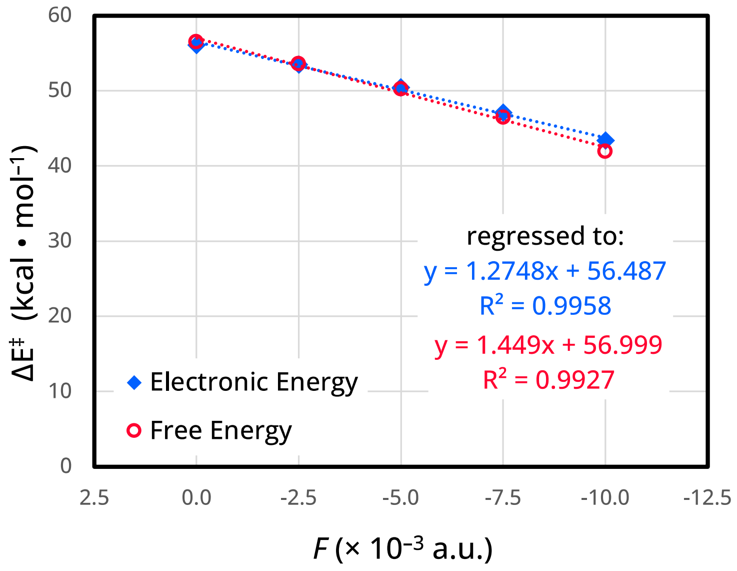

Figure S48. Effective activation energy ($\Delta E^{\ddagger}$ or $\Delta G^{\ddagger}$, kcal/mol) as a function of OEF magnitude for Reaction 12 computed at the B3LYP/def2-TZVP level of theory (gas phase, 298 K).

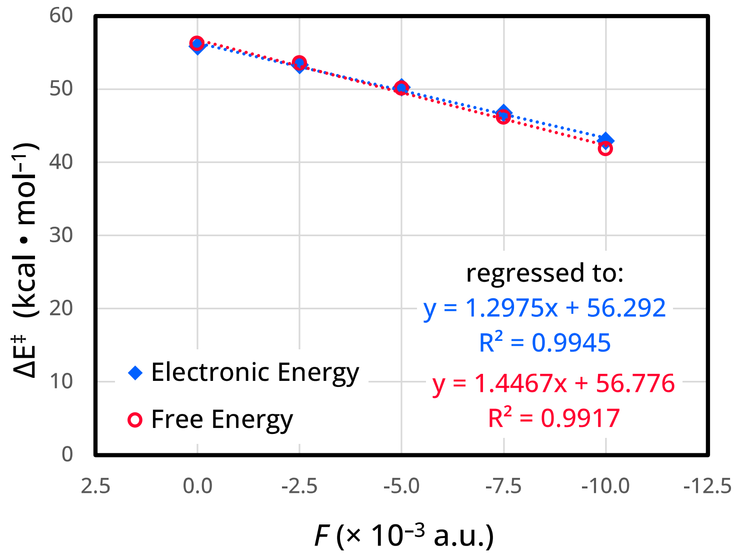

Figure S49. Effective activation energy ($\Delta E^{\ddagger}$ or $\Delta G^{\ddagger}$, kcal/mol) as a function of OEF magnitude for Reaction 12 computed at the B3LYP-D3/def2-TZVP level of theory (gas phase, 298 K).

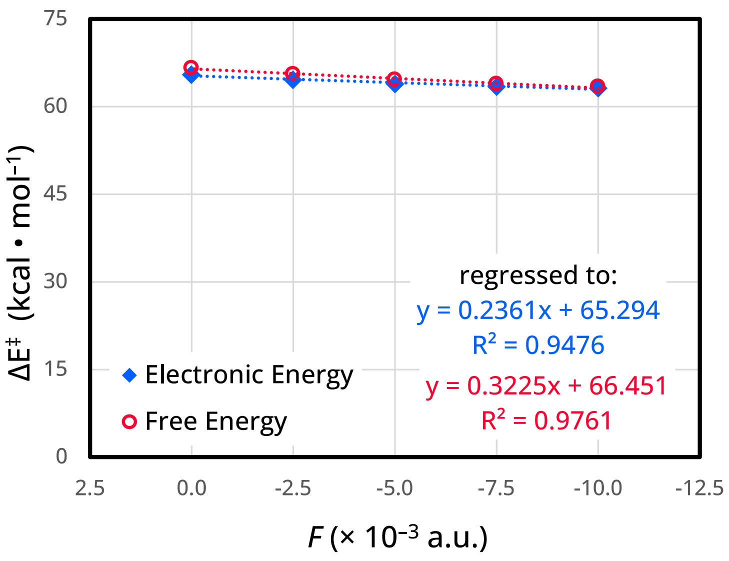

Figure S50. Effective activation energy ($\Delta E^{\ddagger}$ or $\Delta G^{\ddagger}$, kcal/mol) as a function of OEF magnitude for Reaction 12 computed at the M06-2X/def2-TZVP level of theory (gas phase, 298 K).

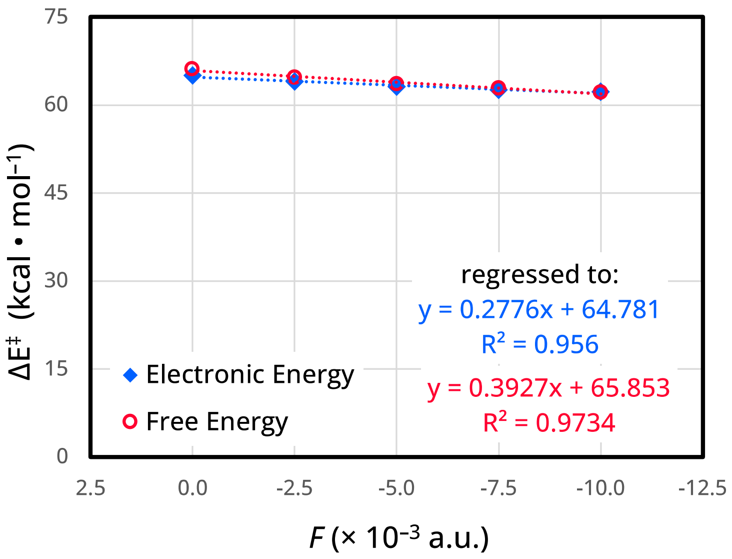

Figure S51. Effective activation energy ($\Delta E^{\ddagger}$ or $\Delta G^{\ddagger}$, kcal/mol) as a function of OEF magnitude for Reaction 12 computed at the ωB97X-D/def2-TZVP level of theory (gas phase, 298 K).

Table S68. Summary of A.V.E.D.A. output data for Reaction 12 computed at the B3LYP/def2-TZVP level of theory (gas phase, 298 K).

**Dipole Moments (debeye)**					
μ(Int) (x,y,z)	[-1.1653, 0.8181, -0.0002]			||μ(Int)||	1.4238
μ(TS) (x,y,z)	[-4.7285, 2.3755, 0.0]			||μ(TS)||	5.2917
μ(rxn) (x,y,z)	[-3.5632, 1.5574, 0.0002]			||μ(rxn)||	3.8887

**Oriented Electric Field**					
F [× 10^{-3} a.u.]	0	-2.5	-5	-7.5	-10
**Electronic Energies**					
Int [a.u.]	-5225.7273	-5225.7292	-5225.7322	-5225.7364	-5225.7420
TS [a.u.]	-5225.6378	-5225.6439	-5225.6518	-5225.6613	-5225.6728
$\Delta E^{\ddagger}$ [kcal/mol]	56.13	53.49	50.46	47.09	43.39
$\Delta\Delta E^{\ddagger}$ [kcal/mol]	0.00	-2.64	-5.68	-9.04	-12.74
**Free Energies**					
Int [a.u.]	-5225.7331	-5225.7344	-5225.7369	-5225.7408	-5225.7461
TS [a.u.]	-5225.6430	-5225.6490	-5225.6568	-5225.6667	-5225.6793
$\Delta G^{\ddagger}$ [kcal/mol]	56.50	53.60	50.24	46.49	41.95
$\Delta\Delta G^{\ddagger}$ [kcal/mol]	0.00	-2.91	-6.27	-10.02	-14.56
**RMSD from 0-Field**					
Int (Å)	-	0.0453	0.0858	0.1199	0.1509
TS (Å)	-	0.0776	0.0493	0.0564	0.0572

Table S69. Summary of A.V.E.D.A. output data for Reaction 12 computed at the B3LYP-D3/def2-TZVP level of theory (gas phase, 298 K).

**Dipole Moments (debeye)**					
μ(Int) (x,y,z)	[-1.0474, 0.776, -0.0003]			||μ(Int)||	1.3035
μ(TS) (x,y,z)	[-4.3027, 2.3054, -0.0]			||μ(TS)||	4.8814
μ(rxn) (x,y,z)	[-3.2553, 1.5294, 0.0003]			||μ(rxn)||	3.5967

**Oriented Electric Field**					
F [× 10^{-3} a.u.]	0	-2.5	-5	-7.5	-10
**Electronic Energies**					
Int [a.u.]	-5225.7303	-5225.7321	-5225.7349	-5225.7390	-5225.7445
TS [a.u.]	-5225.6413	-5225.6471	-5225.6549	-5225.6645	-5225.6761
$\Delta E^‡$ [kcal/mol]	55.87	53.29	50.20	46.73	42.93
$\Delta\Delta E^‡$ [kcal/mol]	0.00	-2.57	-5.66	-9.13	-12.94
**Free Energies**					
Int [a.u.]	-5225.7363	-5225.7376	-5225.7400	-5225.7436	-5225.7488
TS [a.u.]	-5225.6468	-5225.6523	-5225.6602	-5225.6701	-5225.6822
$\Delta G^‡$ [kcal/mol]	56.18	53.53	50.09	46.12	41.80
$\Delta\Delta G^‡$ [kcal/mol]	0.00	-2.65	-6.09	-10.06	-14.38
**RMSD from 0-Field**					
Int (Å)	-	0.0442	0.0928	0.1351	0.1703
TS (Å)	-	0.1083	0.0652	0.0702	0.0648

Table S70. Summary of A.V.E.D.A. output data for Reaction 12 computed at the M06-2X/def2-TZVP level of theory (gas phase, 298 K).

Dipole Moments (debeye)					
μ(Int) (x,y,z)	[-0.3068, 0.9904, -0.0021]		‖μ(Int)‖	1.0368	
μ(TS) (x,y,z)	[-0.4164, 2.549, 0.0]		‖μ(TS)‖	2.5828	
μ(rxn) (x,y,z)	[-0.1096, 1.5586, 0.0021]		‖μ(rxn)‖	1.5625	

**Oriented Electric Field**					
F [× 10^{-3} a.u.]	0	-2.5	-5	-7.5	-10
**Electronic Energies**					
Int [a.u.]	-5225.7058	-5225.7072	-5225.7093	-5225.7124	-5225.7165
TS [a.u.]	-5225.6014	-5225.6042	-5225.6075	-5225.6114	-5225.6158
ΔE‡ [kcal/mol]	65.51	64.62	63.89	63.37	63.18
ΔΔE‡ [kcal/mol]	0.00	-0.89	-1.63	-2.14	-2.33
**Free Energies**					
Int [a.u.]	-5225.7104	-5225.7117	-5225.7135	-5225.7163	-5225.7202
TS [a.u.]	-5225.6043	-5225.6071	-5225.6105	-5225.6145	-5225.6191
ΔG‡ [kcal/mol]	66.62	65.63	64.61	63.88	63.46
ΔΔG‡ [kcal/mol]	0.00	-0.99	-2.01	-2.74	-3.16
**RMSD from 0-Field**					
Int (Å)	-	0.0223	0.0467	0.0743	0.1102
TS (Å)	-	0.0063	0.0131	0.0198	0.0274

Table S71. Summary of A.V.E.D.A. output data for Reaction 12 computed at the ωB97X-D/def2-TZVP level of theory (gas phase, 298 K).

**Dipole Moments (debeye)**					
μ(Int) (x,y,z)	[-0.2949, 0.9043, -0.0006]			||μ(Int)||	0.9512
μ(TS) (x,y,z)	[-0.4571, 2.6509, 0.0005]			||μ(TS)||	2.6900
μ(rxn) (x,y,z)	[-0.1622, 1.7466, 0.0011]			||μ(rxn)||	1.7541

**Oriented Electric Field**					
F [× 10^{-3} a.u.]	0	-2.5	-5	-7.5	-10
**Electronic Energies**					
Int [a.u.]	-5225.7542	-5225.7555	-5225.7576	-5225.7607	-5225.7649
TS [a.u.]	-5225.6506	-5225.6535	-5225.6570	-5225.6610	-5225.6657
$\Delta E^{\ddagger}$ [kcal/mol]	65.01	64.00	63.15	62.54	62.27
$\Delta\Delta E^{\ddagger}$ [kcal/mol]	0.00	-1.02	-1.87	-2.48	-2.74
**Free Energies**					
Int [a.u.]	-5225.7597	-5225.7603	-5225.7621	-5225.7652	-5225.7688
TS [a.u.]	-5225.6543	-5225.6572	-5225.6608	-5225.6650	-5225.6698
$\Delta G^{\ddagger}$ [kcal/mol]	66.15	64.69	63.59	62.87	62.15
$\Delta\Delta G^{\ddagger}$ [kcal/mol]	0.00	-1.46	-2.56	-3.27	-4.00
**RMSD from 0-Field**					
Int (Å)	-	0.0317	0.0699	0.1061	0.1422
TS (Å)	-	0.0082	0.0175	0.0268	0.0382

Table S72. Summary of A.V.E.D.A. output data for Reaction 12 computed in the gas phase at 298 K.

Level of Theory	F = 0 a.u.			F = 2.5 10^{-3} a.u.				F = 5.0 10^{-3} a.u.			
	E_{Int}	E_{TS}	ΔE‡	E_{Int}	E_{TS}	ΔE‡	ΔΔE‡	E_{Int}	E_{TS}	ΔE‡	ΔΔE‡
B3LYP/def2-TZVP											
Electronic Energy	-5225.7273	-5225.6378	56.13	-5225.7292	-5225.6439	53.49	-2.64	-5225.7322	-5225.6518	50.46	-5.68
Enthalpy	-5225.6913	-5225.6041	54.66	-5225.6931	-5225.6102	52.04	-2.62	-5225.6961	-5225.6180	49.04	-5.62
Free Energy	-5225.7331	-5225.6430	56.50	-5225.7344	-5225.6490	53.60	-2.91	-5225.7369	-5225.6568	50.24	-6.27
B3LYP-D3/def2-TZVP											
Electronic Energy	-5225.7303	-5225.6413	55.87	-5225.7321	-5225.6471	53.29	-2.57	-5225.7349	-5225.6549	50.20	-5.66
Enthalpy	-5225.6943	-5225.6076	54.41	-5225.6961	-5225.6134	51.84	-2.57	-5225.6989	-5225.6212	48.79	-5.62
Free Energy	-5225.7363	-5225.6468	56.18	-5225.7376	-5225.6523	53.53	-2.65	-5225.7400	-5225.6602	50.09	-6.09
M06-2X/def2-TZVP											
Electronic Energy	-5225.7058	-5225.6014	65.51	-5225.7072	-5225.6042	64.62	-0.89	-5225.7093	-5225.6075	63.89	-1.63
Enthalpy	-5225.6692	-5225.5668	64.26	-5225.6706	-5225.5696	63.39	-0.87	-5225.6728	-5225.5729	62.66	-1.59
Free Energy	-5225.7104	-5225.6043	66.62	-5225.7117	-5225.6071	65.63	-0.99	-5225.7135	-5225.6105	64.61	-2.01
ωB97X-D/def2-TZVP											
Electronic Energy	-5225.7542	-5225.6506	65.01	-5225.7555	-5225.6535	64.00	-1.02	-5225.7576	-5225.6570	63.15	-1.87
Enthalpy	-5225.7178	-5225.6162	63.74	-5225.7191	-5225.6191	62.74	-1.00	-5225.7212	-5225.6226	61.91	-1.83
Free Energy	-5225.7597	-5225.6543	66.15	-5225.7603	-5225.6572	64.69	-1.46	-5225.7621	-5225.6608	63.59	-2.56

Table S73. Summary of A.V.E.D.A. output data for Reaction 12 computed in the gas phase at 298 K, continued

Level of Theory	F = 0 a.u.			F = 7.5 10^{-3} a.u.				F = 10.0 10^{-3} a.u.			
	E_{Int}	E_{TS}	$\Delta E^{‡}$	E_{Int}	E_{TS}	$\Delta E^{‡}$	$\Delta\Delta E^{‡}$	E_{Int}	E_{TS}	$\Delta E^{‡}$	$\Delta\Delta E^{‡}$
B3LYP/def2-TZVP											
Electronic Energy	-5225.7273	-5225.6378	56.13	-5225.7364	-5225.6613	47.09	-9.04	-5225.7420	-5225.6728	43.40	-12.74
Enthalpy	-5225.6913	-5225.6041	54.66	-5225.7004	-5225.6275	45.74	-8.92	-5225.7060	-5225.6388	42.17	-12.49
Free Energy	-5225.7331	-5225.6430	56.50	-5225.7408	-5225.6613	49.84	-6.66	-5225.7461	-5225.6793	41.95	-14.56
B3LYP-D3/def2-TZVP											
Electronic Energy	-5225.7303	-5225.6413	55.87	-5225.7390	-5225.6645	46.73	-9.13	-5225.7445	-5225.6761	42.93	-12.94
Enthalpy	-5225.6943	-5225.6076	54.41	-5225.7030	-5225.6307	45.38	-9.03	-5225.7086	-5225.6421	41.69	-12.72
Free Energy	-5225.7363	-5225.6468	56.18	-5225.7436	-5225.6701	46.12	-10.06	-5225.7488	-5225.6822	41.80	-14.38
M06-2X/def2-TZVP											
Electronic Energy	-5225.7058	-5225.6014	65.51	-5225.7124	-5225.6114	63.37	-2.14	-5225.7165	-5225.6158	63.19	-2.33
Enthalpy	-5225.6692	-5225.5668	64.26	-5225.6759	-5225.5768	62.17	-2.09	-5225.6800	-5225.5812	62.00	-2.26
Free Energy	-5225.7104	-5225.6043	66.62	-5225.7163	-5225.6145	63.89	-2.74	-5225.7202	-5225.6191	63.46	-3.16
ωB97X-D/def2-TZVP											
Electronic Energy	-5225.7542	-5225.6506	65.01	-5225.7607	-5225.6610	62.54	-2.48	-5225.7649	-5225.6657	62.27	-2.74
Enthalpy	-5225.7178	-5225.6162	63.74	-5225.7244	-5225.6266	61.34	-2.41	-5225.7286	-5225.6313	61.09	-2.66
Free Energy	-5225.7597	-5225.6543	66.15	-5225.7652	-5225.6650	62.88	-3.27	-5225.7688	-5225.6698	62.15	-4.00

4.15 Reaction 13 (Palladium-Mediated C–H Activation)

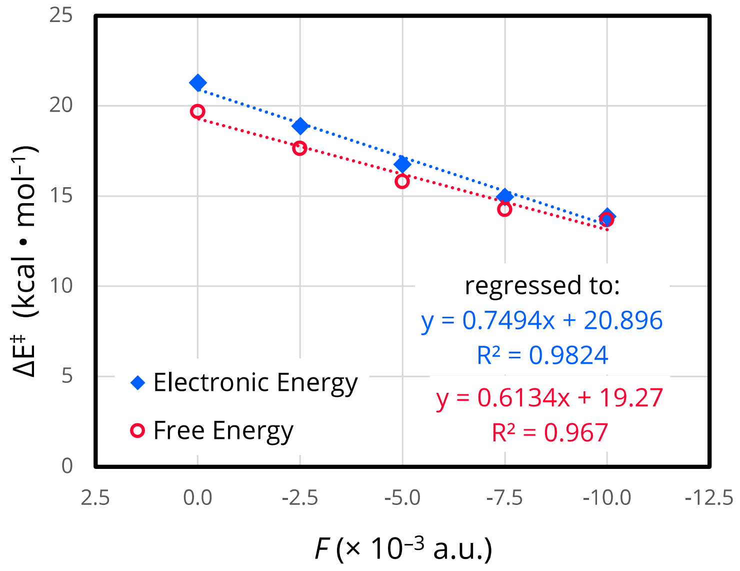

Figure S52. Effective activation energy ($\Delta E^{\ddagger}$ or $\Delta G^{\ddagger}$, kcal/mol) as a function of OEF magnitude for Reaction 13 computed at the B3LYP/def2-TZVP level of theory (gas phase, 298 K).

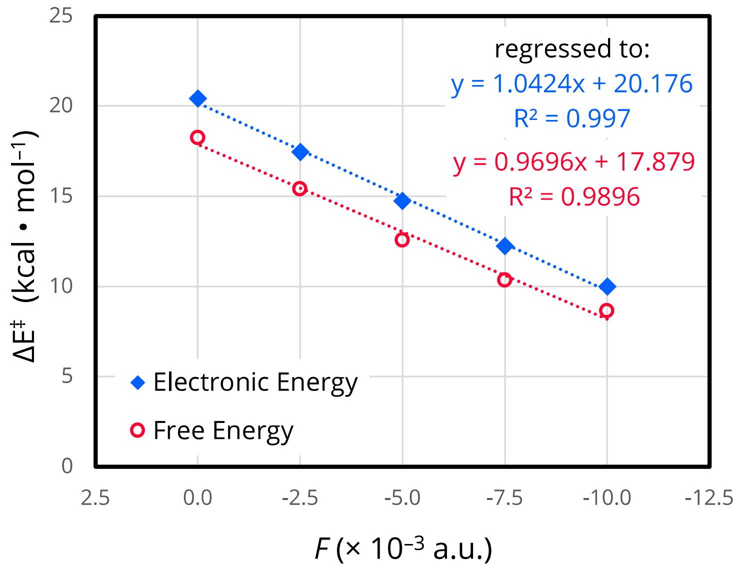

Figure S53. Effective activation energy ($\Delta E^{\ddagger}$ or $\Delta G^{\ddagger}$, kcal/mol) as a function of OEF magnitude for Reaction 13 computed at the B3LYP-D3/def2-TZVP level of theory (gas phase, 298 K).

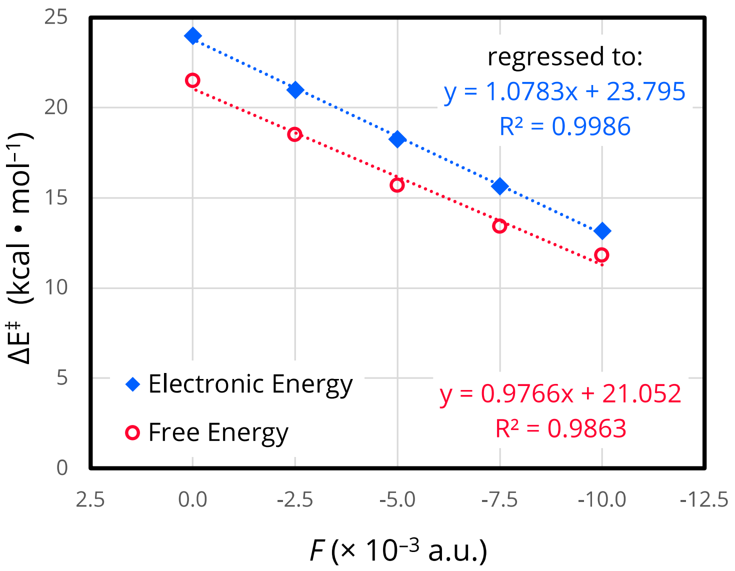

Figure S54. Effective activation energy ($\Delta E^{\ddagger}$ or $\Delta G^{\ddagger}$, kcal/mol) as a function of OEF magnitude for Reaction 13 computed at the M06-2X/def2-TZVP level of theory (gas phase, 298 K).

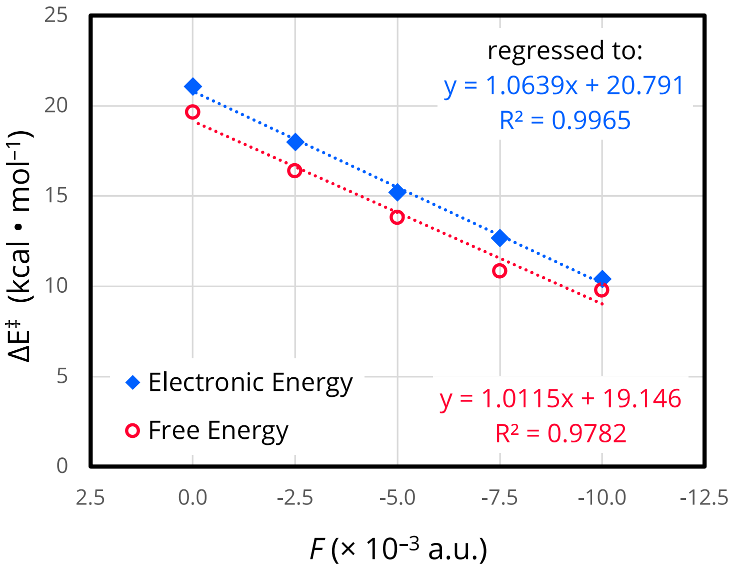

Figure S55. Effective activation energy ($\Delta E^{\ddagger}$ or $\Delta G^{\ddagger}$, kcal/mol) as a function of OEF magnitude for Reaction 13 computed at the ωB97X-D/def2-TZVP level of theory (gas phase, 298 K).

Table S74. Summary of A.V.E.D.A. output data for Reaction 13 computed at the B3LYP/def2-TZVP level of theory (gas phase, 298 K).

**Dipole Moments (debeye)**					
μ(Int) (x,y,z)	[-4.3572, 0.0885, 1.7039]			||μ(Int)||	4.6793
μ(TS) (x,y,z)	[-4.7367, -3.0753, -0.8332]			||μ(TS)||	5.7086
μ(rxn) (x,y,z)	[-0.3795, -3.1638, -2.5371]			||μ(rxn)||	4.0731

**Oriented Electric Field**					
F [× 10^{-3} a.u.]	0	-2.5	-5	-7.5	-10
**Electronic Energies**					
Int [a.u.]	-990.8500	-990.8503	-990.8526	-990.8569	-990.8640
TS [a.u.]	-990.8161	-990.8202	-990.8259	-990.8331	-990.8419
ΔE‡ [kcal/mol]	21.27	18.89	16.76	14.95	13.87
ΔΔE‡ [kcal/mol]	0	-2.38	-4.51	-6.32	-7.40
**Free Energies**					
Int [a.u.]	-990.5966	-990.5976	-990.6003	-990.6050	-990.6126
TS [a.u.]	-990.5653	-990.5695	-990.5752	-990.5823	-990.5908
ΔG‡ [kcal/mol]	19.66	17.62	15.79	14.26	13.6782
ΔΔG‡ [kcal/mol]	0	-2.04	-3.88	-5.41	-5.99
**RMSD from 0-Field**					
Int (Å)	-	0.0848	0.0998	0.1906	0.6296
TS (Å)	-	0.0145	0.0259	0.0320	0.0368

Table S75. Summary of A.V.E.D.A. output data for Reaction 13 computed at the B3LYP-D3/def2-TZVP level of theory (gas phase, 298 K).

**Dipole Moments (debeye)**					
μ(Int) (x,y,z)	[-3.6247, 0.2547, 2.8583]			||μ(Int)||	4.6231
μ(TS) (x,y,z)	[-4.6054, -3.0291, -0.866]			||μ(TS)||	5.5799
μ(rxn) (x,y,z)	[-0.9807, -3.2838, -3.7243]			||μ(rxn)||	5.0612

**Oriented Electric Field**					
F [× 10^{-3} a.u.]	0	-2.5	-5	-7.5	-10
**Electronic Energies**					
Int [a.u.]	-990.8939	-990.8933	-990.8946	-990.8978	-990.9029
TS [a.u.]	-990.8614	-990.8655	-990.8711	-990.8783	-990.8870
$\Delta E^{\ddagger}$ [kcal/mol]	20.42	17.44	14.73	12.23	9.99
$\Delta\Delta E^{\ddagger}$ [kcal/mol]	0.00	-2.97	-5.68	-8.18	-10.42
**Free Energies**					
Int [a.u.]	-990.6388	-990.6387	-990.6400	-990.6433	-990.6490
TS [a.u.]	-990.6097	-990.6141	-990.6199	-990.6268	-990.6352
$\Delta G^{\ddagger}$ [kcal/mol]	18.22	15.40	12.56	10.34	8.64
$\Delta\Delta G^{\ddagger}$ [kcal/mol]	0.00	-2.82	-5.66	-7.89	-9.59
**RMSD from 0-Field**					
Int (Å)	-	0.0098	0.0163	0.0329	0.0352
TS (Å)	-	0.0092	0.0246	0.0334	0.0382

Table S76. Summary of A.V.E.D.A. output data for Reaction 13 computed at the M06-2X/def2-TZVP level of theory (gas phase, 298 K).

**Dipole Moments (debeye)**					
μ(Int) (x,y,z)	[-3.5212, -0.298, 3.0651]			‖μ(Int)‖	4.6779
μ(TS) (x,y,z)	[-4.5786, -3.3228, -0.8437]			‖μ(TS)‖	5.7198
μ(rxn) (x,y,z)	[-1.0574, -3.0248, -3.9088]			‖μ(rxn)‖	5.0543

**Oriented Electric Field**					
F [× 10^{-3} a.u.]	0	-2.5	-5	-7.5	-10
**Electronic Energies**					
Int [a.u.]	-990.3365	-990.3360	-990.3373	-990.3403	-990.3452
TS [a.u.]	-990.2983	-990.3026	-990.3082	-990.3154	-990.3242
$\Delta E^‡$ [kcal/mol]	23.98	20.99	18.24	15.64	13.17
$\Delta\Delta E^‡$ [kcal/mol]	0.00	-2.99	-5.74	-8.34	-10.81
**Free Energies**					
Int [a.u.]	-990.0780	-990.0773	-990.0787	-990.0820	-990.0873
TS [a.u.]	-990.0438	-990.0478	-990.0537	-990.0606	-990.0685
$\Delta G^‡$ [kcal/mol]	21.48	18.48	15.67	13.41	11.81
$\Delta\Delta G^‡$ [kcal/mol]	0.00	-2.99	-5.81	-8.07	-9.67
**RMSD from 0-Field**					
Int (Å)	-	0.0094	0.0213	0.0342	0.0640
TS (Å)	-	0.0099	0.0204	0.0219	0.0229

Table S77. Summary of A.V.E.D.A. output data for Reaction 13 computed at the ωB97X-D/def2-TZVP level of theory (gas phase, 298 K).

**Dipole Moments (debeye)**					
μ(Int) (x,y,z)	[-3.5816, 0.2299, 2.939]			‖μ(Int)‖	4.6388
μ(TS) (x,y,z)	[-4.6792, -3.1881, -0.8698]			‖μ(TS)‖	5.7285
μ(rxn) (x,y,z)	[-1.0976, -3.418, -3.8088]			‖μ(rxn)‖	5.2340

**Oriented Electric Field**					
F [× 10^{-3} a.u.]	0	-2.5	-5	-7.5	-10
**Electronic Energies**					
Int [a.u.]	-990.5584	-990.5578	-990.5591	-990.5622	-990.5672
TS [a.u.]	-990.5248	-990.5291	-990.5348	-990.5420	-990.5506
$\Delta E^{\ddagger}$ [kcal/mol]	21.06	17.98	15.21	12.69	10.41
$\Delta\Delta E^{\ddagger}$ [kcal/mol]	0.00	-3.08	-5.85	-8.38	-10.65
**Free Energies**					
Int [a.u.]	-990.3004	-990.2996	-990.3012	-990.3045	-990.3104
TS [a.u.]	-990.2691	-990.2735	-990.2792	-990.2872	-990.2949
$\Delta G^{\ddagger}$ [kcal/mol]	19.63	16.39	13.81	10.84	9.77
$\Delta\Delta G^{\ddagger}$ [kcal/mol]	0.00	-3.24	-5.82	-8.80	-9.87
**RMSD from 0-Field**					
Int (Å)	-	0.0174	0.0191	0.0244	0.1039
TS (Å)	-	0.0062	0.0117	0.0204	0.0284

Table S78. Summary of A.V.E.D.A. output data for Reaction 13 computed in the gas phase at 298 K.

Level of Theory	F = 0 a.u.			F = 2.5 10^{-3} a.u.				F = 5.0 10^{-3} a.u.			
	E_{Int}	E_{TS}	$\Delta E^{‡}$	E_{Int}	E_{TS}	$\Delta E^{‡}$	$\Delta\Delta E^{‡}$	E_{Int}	E_{TS}	$\Delta E^{‡}$	$\Delta\Delta E^{‡}$
B3LYP/def2-TZVP											
Electronic Energy	-990.8500	-990.8161	21.28	-990.8503	-990.8202	18.89	-2.39	-990.8526	-990.8259	16.76	-4.51
Enthalpy	-990.5201	-990.4923	17.45	-990.5204	-990.4963	15.09	-2.36	-990.5227	-990.5020	13.00	-4.45
Free Energy	-990.5966	-990.5653	19.66	-990.5976	-990.5695	17.62	-2.04	-990.6003	-990.5752	15.79	-3.88
B3LYP-D3/def2-TZVP											
Electronic Energy	-990.8939	-990.8614	20.42	-990.8933	-990.8655	17.44	-2.98	-990.8946	-990.8711	14.73	-5.69
Enthalpy	-990.5634	-990.5372	16.46	-990.5629	-990.5413	13.50	-2.95	-990.5642	-990.5470	10.82	-5.64
Free Energy	-990.6388	-990.6097	18.22	-990.6387	-990.6141	15.40	-2.82	-990.6400	-990.6199	12.56	-5.66
M06-2X/def2-TZVP											
Electronic Energy	-990.3365	-990.2983	23.99	-990.3360	-990.3026	20.99	-3.01	-990.3373	-990.3082	18.24	-5.75
Enthalpy	-990.0032	-989.9710	20.18	-990.0026	-989.9753	17.15	-3.03	-990.0040	-989.9810	14.42	-5.76
Free Energy	-990.0780	-990.0438	21.48	-990.0773	-990.0478	18.48	-2.99	-990.0787	-990.0537	15.67	-5.81
ωB97X-D/def2-TZVP											
Electronic Energy	-990.5584	-990.5248	21.08	-990.5578	-990.5291	17.98	-3.10	-990.5591	-990.5348	15.21	-5.87
Enthalpy	-990.2249	-990.1972	17.36	-990.2242	-990.2015	14.23	-3.13	-990.2256	-990.2073	11.52	-5.84
Free Energy	-990.3004	-990.2691	19.63	-990.2996	-990.2735	16.39	-3.24	-990.3012	-990.2792	13.81	-5.82

Table S79. Summary of A.V.E.D.A. output data for Reaction 13 computed in the gas phase at 298 K, continued

Level of Theory	F = 0 a.u.			F = 7.5 10^{-3} a.u.				F = 10.0 10^{-3} a.u.			
	E_{Int}	E_{TS}	$\Delta E^{‡}$	E_{Int}	E_{TS}	$\Delta E^{‡}$	$\Delta\Delta E^{‡}$	E_{Int}	E_{TS}	$\Delta E^{‡}$	$\Delta\Delta E^{‡}$
B3LYP/def2-TZVP											
Electronic Energy	-990.8500	-990.8161	21.28	-990.8569	-990.8331	14.95	-6.32	-990.8640	-990.8418	13.87	-7.41
Enthalpy	-990.5201	-990.4923	17.45	-990.5272	-990.5093	11.24	-6.21	-990.5345	-990.5182	10.26	-7.20
Free Energy	-990.5966	-990.5653	19.66	-990.6050	-990.5822	14.26	-5.41	-990.6126	-990.5908	13.68	-5.99
B3LYP-D3/def2-TZVP											
Electronic Energy	-990.8939	-990.8614	20.42	-990.8978	-990.8783	12.23	-8.19	-990.9029	-990.8870	9.99	-10.43
Enthalpy	-990.5634	-990.5372	16.46	-990.5675	-990.5542	8.36	-8.10	-990.5729	-990.5630	6.19	-10.27
Free Energy	-990.6388	-990.6097	18.22	-990.6433	-990.6268	10.34	-7.89	-990.6490	-990.6352	8.64	-9.59
M06-2X/def2-TZVP											
Electronic Energy	-990.3365	-990.2983	23.99	-990.3403	-990.3154	15.64	-8.35	-990.3452	-990.3242	13.17	-10.82
Enthalpy	-990.0032	-989.9710	20.18	-990.0072	-989.9883	11.87	-8.31	-990.0122	-989.9972	9.45	-10.73
Free Energy	-990.0780	-990.0438	21.48	-990.0820	-990.0606	13.41	-8.07	-990.0873	-990.0685	11.81	-9.67
ωB97X-D/def2-TZVP											
Electronic Energy	-990.5584	-990.5248	21.08	-990.5622	-990.5420	12.69	-8.39	-990.5672	-990.5506	10.41	-10.67
Enthalpy	-990.2249	-990.1972	17.36	-990.2289	-990.2146	8.99	-8.37	-990.2342	-990.2233	6.85	-10.51
Free Energy	-990.3004	-990.2691	19.63	-990.3045	-990.2872	10.84	-8.80	-990.3104	-990.2949	9.77	-9.87

4.14 Summary of Results by Functional

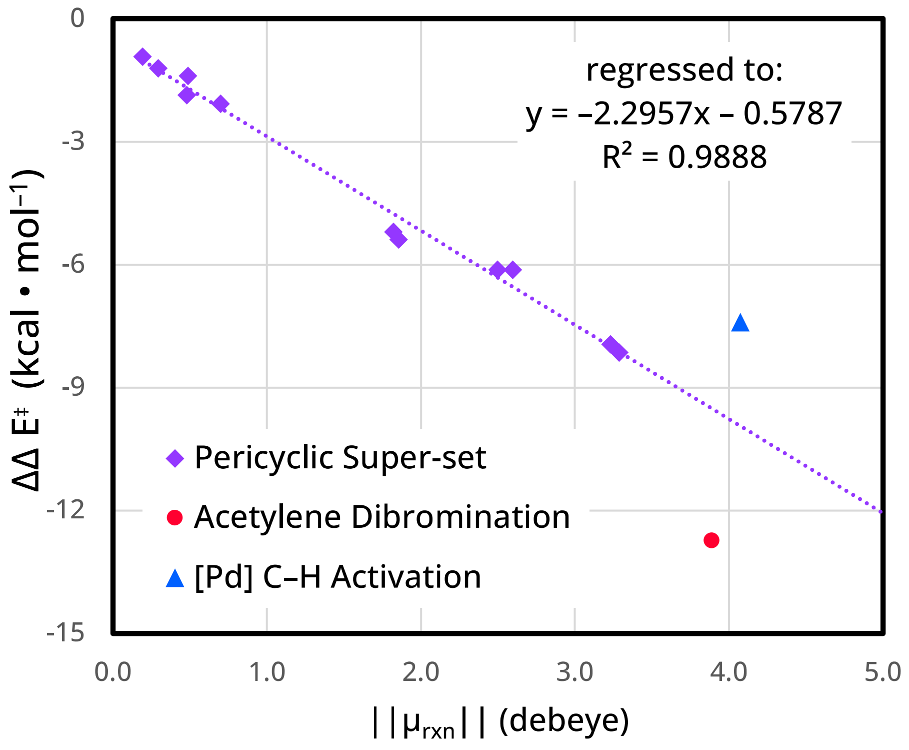

Figure S56. Correlation between net reaction dipole moment magnitude ($\|\vec{\mu}_{rxn}\|$) and change in effective activation energy in the presences of an oriented electric field (10.0×10^{-3} a.u.) for Reactions 1–13 described by the uncorrected electronic energy difference ($\Delta\Delta E^{\ddagger}$), Results computed in the gas phase at 298 K using B3LYP with the def2-TZVP basis set. Regression shown for the pericyclic reaction super-set (Reactions 1–11).

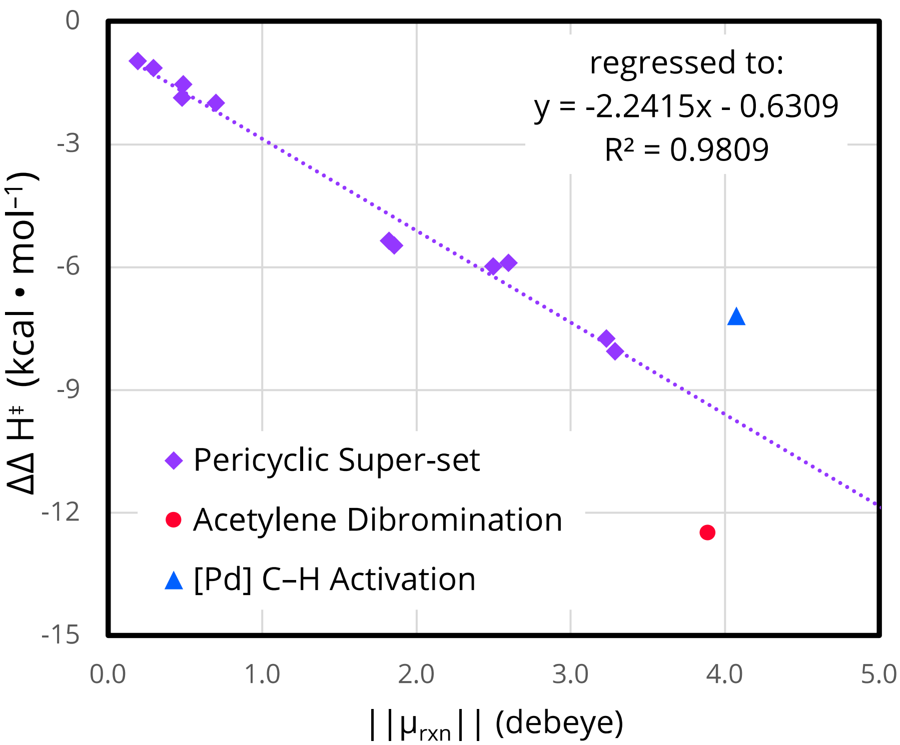

Figure S57. Correlation between net reaction dipole moment magnitude ($\|\vec{\mu}_{rxn}\|$) and change in effective activation energy in the presences of an oriented electric field (10.0×10^{-3} a.u.) for Reactions 1–13 described by the vibrationally corrected enthalpy ($\Delta\Delta H^{\ddagger}$), Results computed in the gas phase at 298 K using B3LYP with the def2-TZVP basis set. Regression shown for the pericyclic reaction super-set (Reactions 1–11).

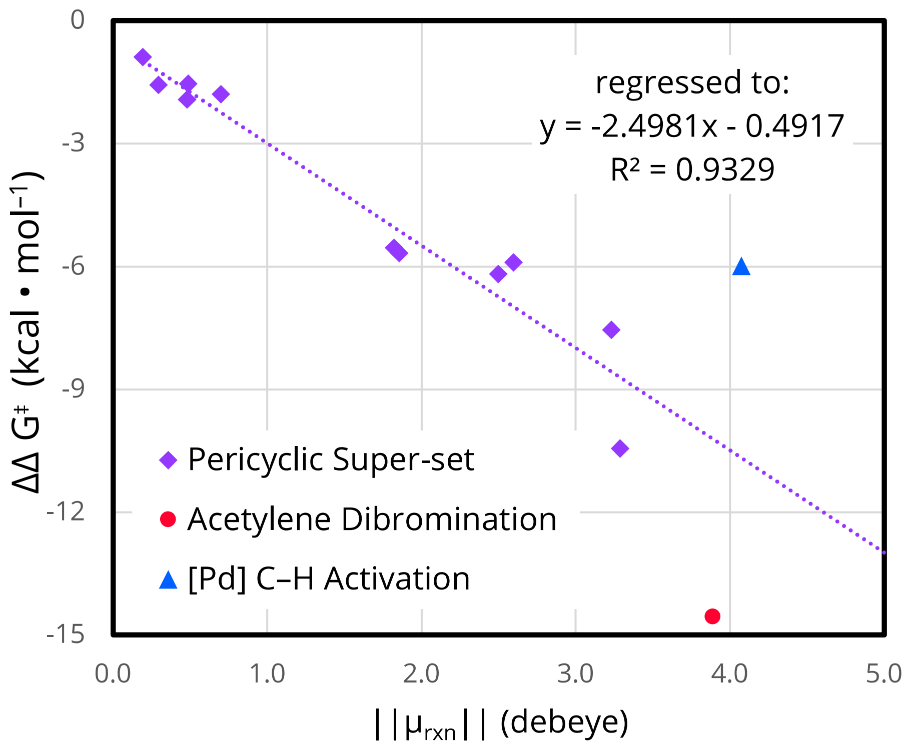

Figure S58. Correlation between net reaction dipole moment magnitude ($\|\vec{\mu}_{rxn}\|$) and change in effective activation energy in the presences of an oriented electric field (10.0×10^{-3} a.u.) for Reactions 1–13 described by the free energy ($\Delta\Delta G^{\ddagger}$), Results computed in the gas phase at 298 K using B3LYP with the def2-TZVP basis set. Regression shown for the pericyclic reaction super-set (Reactions 1–11).

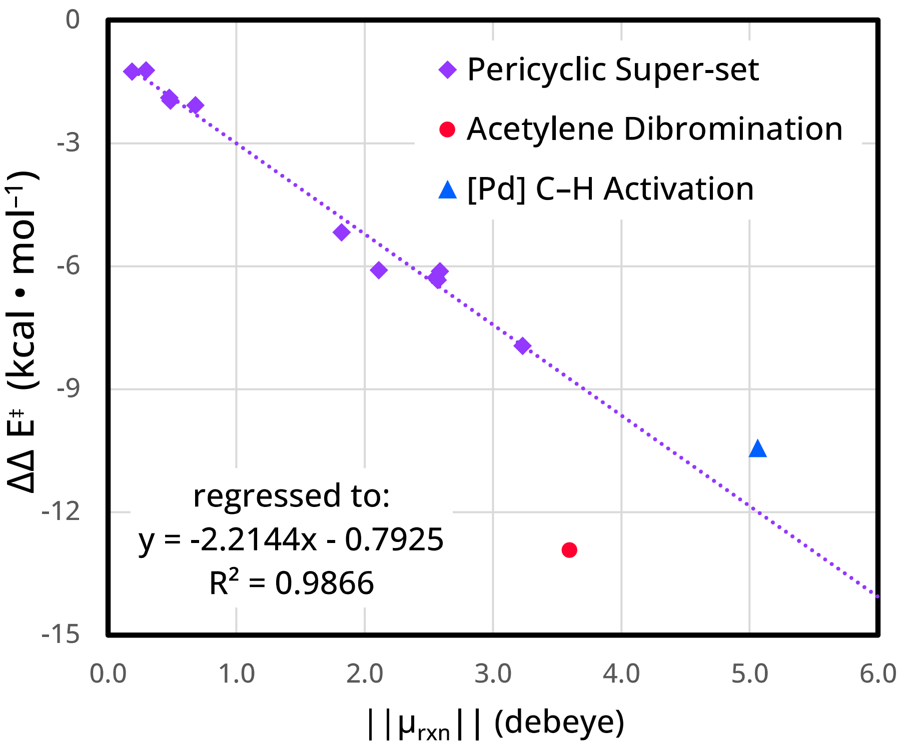

Figure S59. Correlation between net reaction dipole moment magnitude ($\|\vec{\mu}_{rxn}\|$) and change in effective activation energy in the presences of an oriented electric field (10.0×10^{-3} a.u.) for Reactions 1–13 described by the uncorrected electronic energy difference ($\Delta\Delta E^{\ddagger}$), Results computed in the gas phase at 298 K using B3LYP-D3 with the def2-TZVP basis set. Regression shown for the pericyclic reaction super-set (Reactions 1–11).

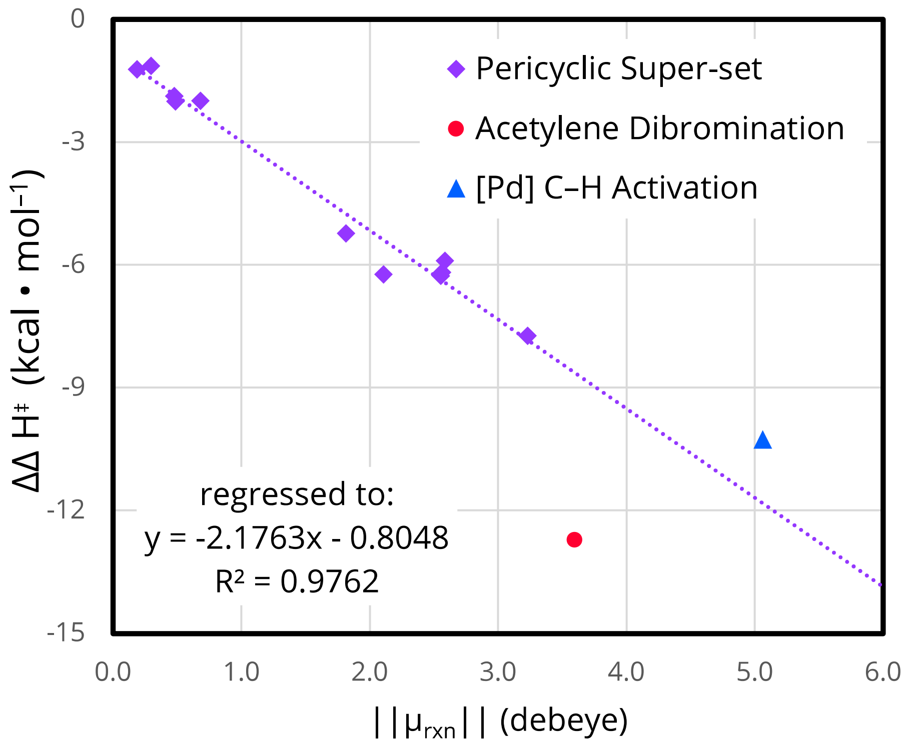

Figure S60. Correlation between net reaction dipole moment magnitude ($\|\vec{\mu}_{rxn}\|$) and change in effective activation energy in the presences of an oriented electric field (10.0×10^{-3} a.u.) for Reactions 1–13 described by the vibrationally corrected enthalpy ($\Delta\Delta H^{\ddagger}$), Results computed in the gas phase at 298 K using B3LYP-D3 with the def2-TZVP basis set. Regression shown for the pericyclic reaction super-set (Reactions 1–11).

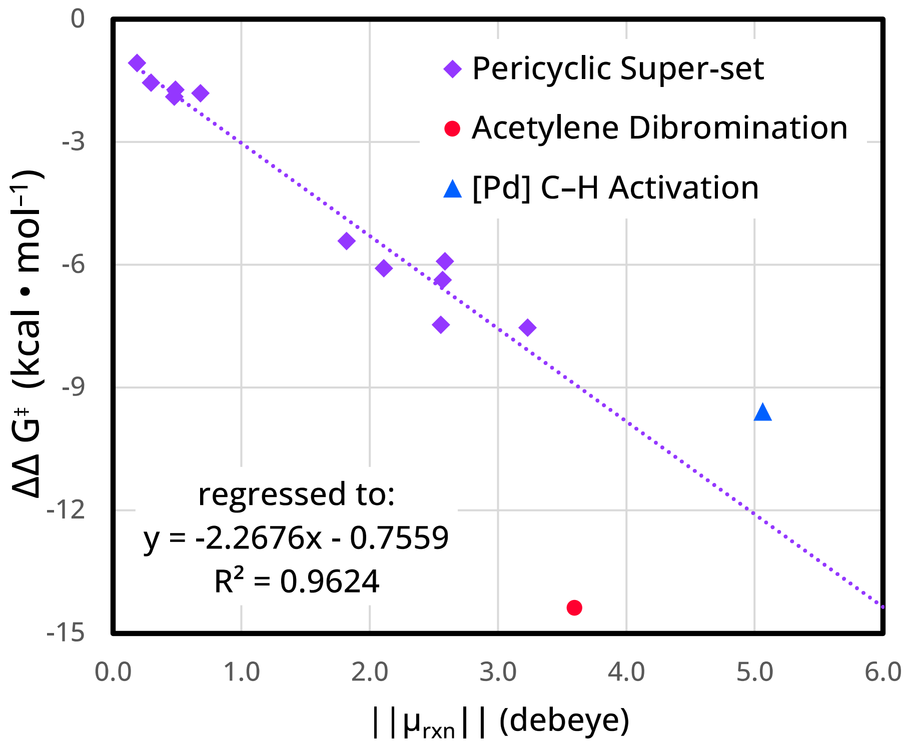

Figure S61. Correlation between net reaction dipole moment magnitude ($\|\vec{\mu}_{rxn}\|$) and change in effective activation energy in the presences of an oriented electric field (10.0×10^{-3} a.u.) for Reactions 1–13 described by the free energy (ΔΔG‡), Results computed in the gas phase at 298 K using B3LYP-D3 with the def2-TZVP basis set. Regression shown for the pericyclic reaction super-set (Reactions 1–11).

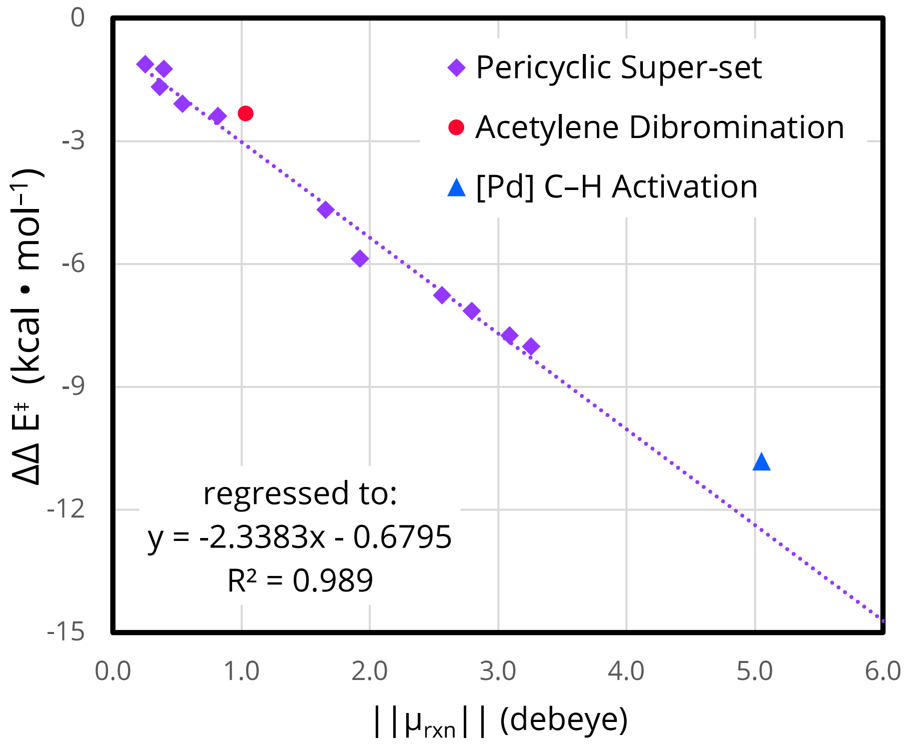

Figure S62. Correlation between net reaction dipole moment magnitude ($\|\vec{\mu}_{rxn}\|$) and change in effective activation energy in the presences of an oriented electric field (10.0×10^{-3} a.u.) for Reactions 1–13 described by the uncorrected electronic energy difference ($\Delta\Delta E^{\ddagger}$), Results computed in the gas phase at 298 K using M06-2X with the def2-TZVP basis set. Regression shown for the pericyclic reaction super-set (Reactions 1–11).

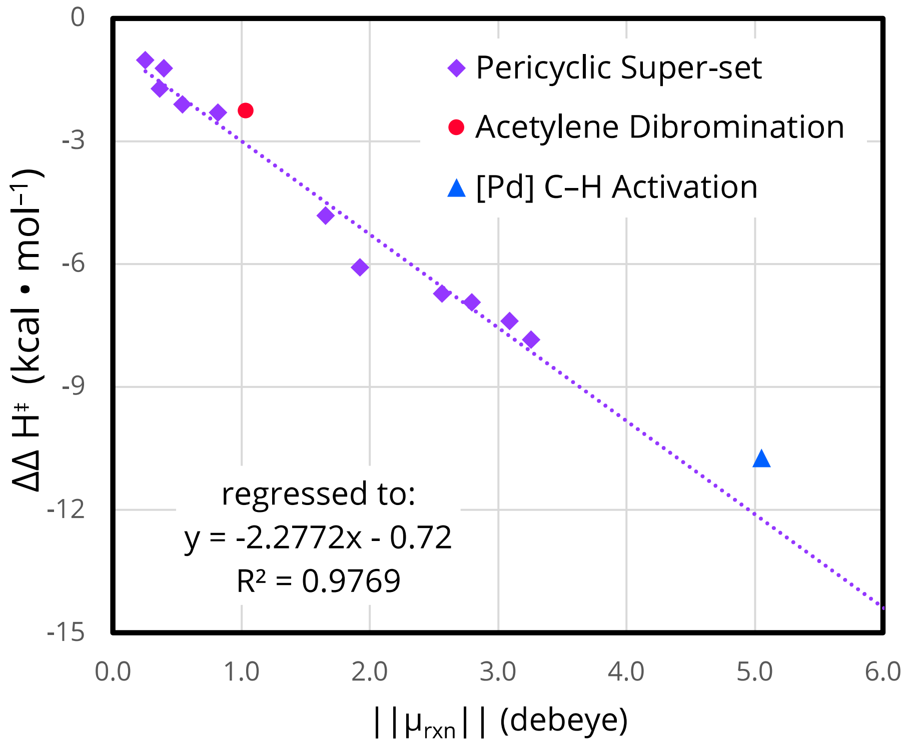

Figure S63. Correlation between net reaction dipole moment magnitude ($\|\vec{\mu}_{rxn}\|$) and change in effective activation energy in the presences of an oriented electric field (10.0×10^{-3} a.u.) for Reactions 1–13 described by the vibrationally corrected enthalpy ($\Delta\Delta H^{\ddagger}$), Results computed in the gas phase at 298 K using M06-2X with the def2-TZVP basis set. Regression shown for the pericyclic reaction super-set (Reactions 1–11).

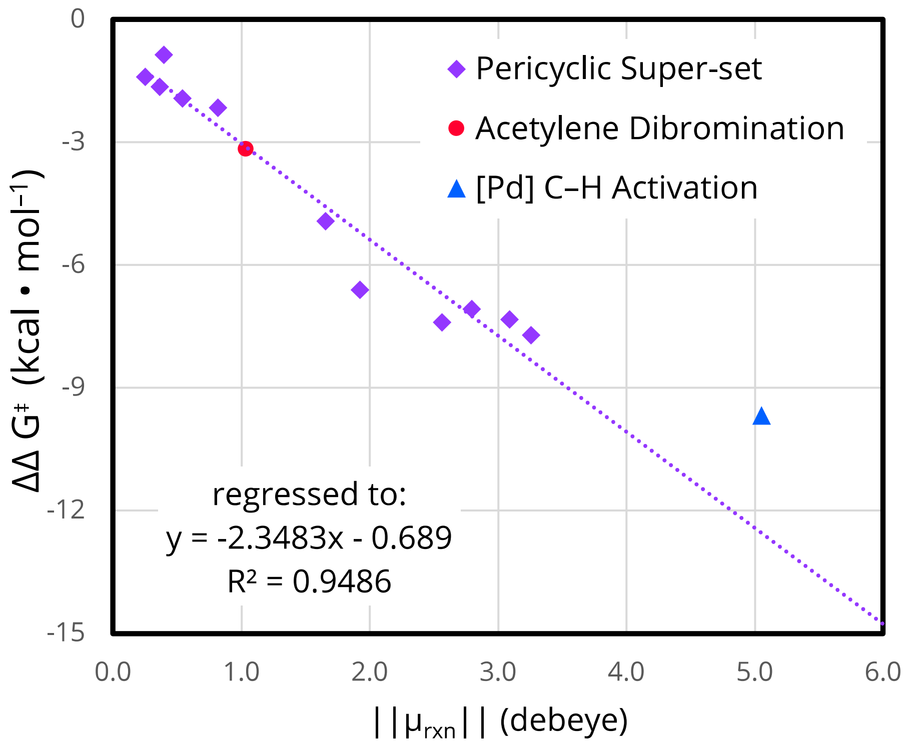

Figure S64. Correlation between net reaction dipole moment magnitude ($\|\vec{\mu}_{rxn}\|$) and change in effective activation energy in the presences of an oriented electric field (10.0×10^{-3} a.u.) for Reactions 1–13 described by the free energy ($\Delta\Delta G^{\ddagger}$), Results computed in the gas phase at 298 K using M06-2X with the def2-TZVP basis set. Regression shown for the pericyclic reaction super-set (Reactions 1–11).

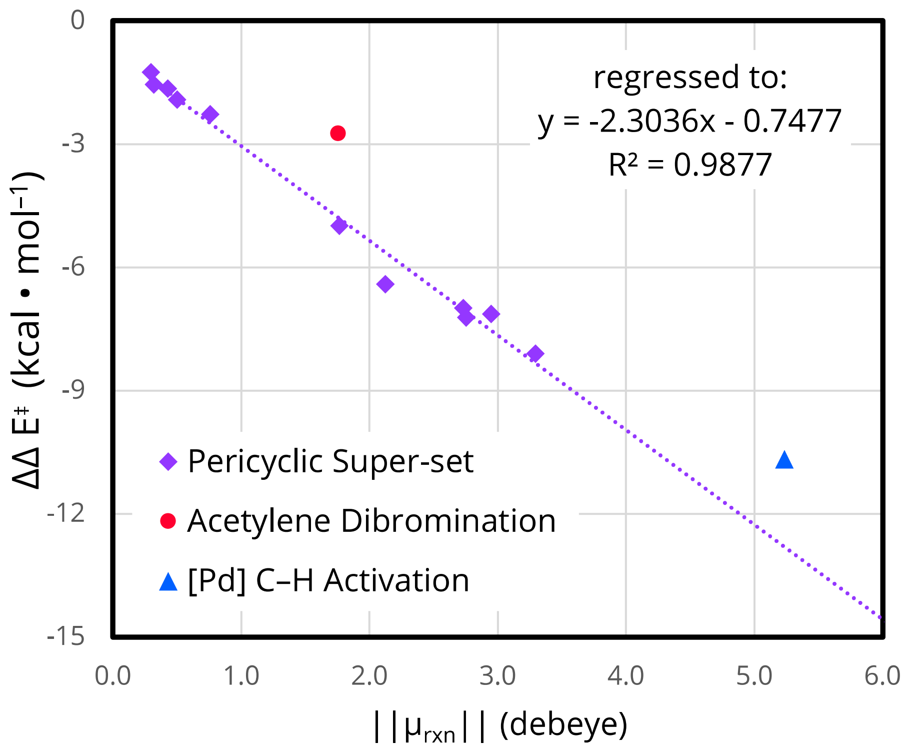

Figure S65. Correlation between net reaction dipole moment magnitude ($\|\vec{\mu}_{rxn}\|$) and change in effective activation energy in the presences of an oriented electric field (10.0×10^{-3} a.u.) for Reactions 1–13 described by the uncorrected electronic energy difference ($\Delta\Delta E^{\ddagger}$), Results computed in the gas phase at 298 K using ωB97X-D with the def2-TZVP basis set. Regression shown for the pericyclic reaction super-set (Reactions 1–11).

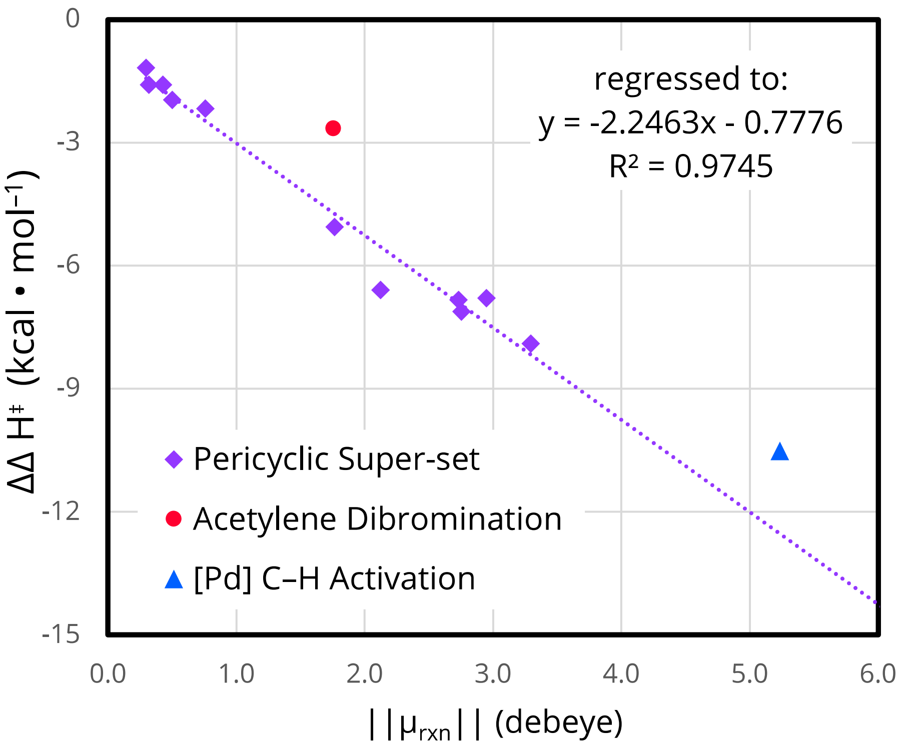

Figure S66. Correlation between net reaction dipole moment magnitude ($\|\vec{\mu}_{rxn}\|$) and change in effective activation energy in the presences of an oriented electric field (10.0×10^{-3} a.u.) for Reactions 1–13 described by the vibrationally corrected enthalpy ($\Delta\Delta H^{\ddagger}$), Results computed in the gas phase at 298 K using ωB97X-D with the def2-TZVP basis set. Regression shown for the pericyclic reaction super-set (Reactions 1–11).

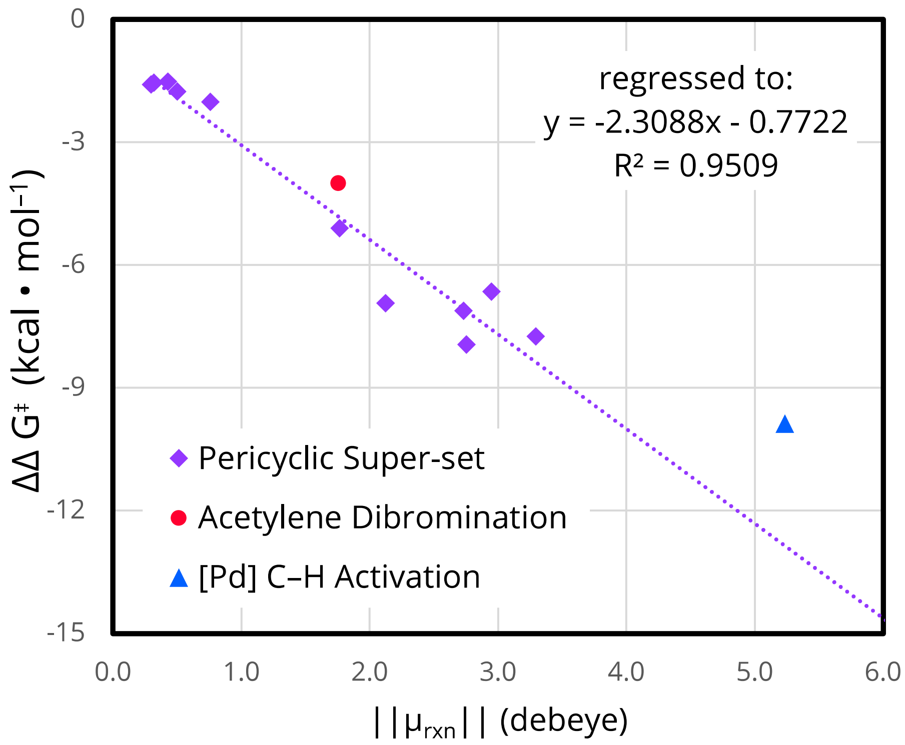

Figure S67. Correlation between net reaction dipole moment magnitude ($\|\vec{\mu}_{rxn}\|$) and change in effective activation energy in the presences of an oriented electric field (10.0 × 10^{-3} a.u.) for Reactions 1–13 described by the free energy ($\Delta\Delta G^{\ddagger}$), Results computed in the gas phase at 298 K using ωB97X-D with the def2-TZVP basis set. Regression shown for the pericyclic reaction super-set (Reactions 1–11).

5. References

1. Yoo, A. B.; Jette, M. A.; Grondona, M. In *SLURM: Simple Linux Utility for Resource Management*, Job Scheduling Strategies for Parallel Processing, Berlin, Heidelberg, Feitelson, D.; Rudolph, L.; Schwiegelshohn, U., Eds. Springer Berlin, Heidelberg, 2003; pp 44–60.
2. Frisch, M. J.; Trucks, G. W.; Schlegel, H. B.; Scuseria, G. E.; Robb, M. A.; Cheeseman, J. R.; Scalmani, G.; Barone, V.; Petersson, G. A.; Nakatsuji, H.; Li, X.; Caricato, M.; Marenich, A. V.; Bloino, J.; Janesko, B. G.; Gomperts, R.; Mennucci, B.; Hratchian, H. P.; Ortiz, J. V.; Izmaylov, A. F.; Sonnenberg, J. L.; Williams-Young, D.; Ding, F.; Lipparini, F.; Egidi, F.; Goings, J.; Peng, B.; Petrone, A.; Henderson, T.; Ranasinghe, D.; Zakrzewski, V. G.; Gao, J.; Rega, N.; Zheng, G.; Liang, W.; Hada, M.; Ehara, M.; Toyota, K.; Fukuda, R.; Hasegawa, J.; Ishida, M.; Nakajima, T.; Honda, Y.; Kitao, O.; Nakai, H.; Vreven, T.; Throssell, K.; Montgomery, J., J. A. ; Peralta, J. E.; Ogliaro, F.; Bearpark, M. J.; Heyd, J. J.; Brothers, E. N.; Kudin, K. N.; Staroverov, V. N.; Keith, T. A.; Kobayashi, R.; Normand, J.; Raghavachari, K.; Rendell, A. P.; Burant, J. C.; Iyengar, S. S.; Tomasi, J.; Cossi, M.; Millam, J. M.; Klene, M.; Adamo, C.; Cammi, R.; Ochterski, J. W.; Martin, R. L.; Morokuma, K.; Farkas, O.; Foresman, J. B.; Fox, D. J. *Gaussian 16, Revision A.03*, Gaussian, Inc.: Wallingford, CT, 2016.
3. *The PyMOL Molecular Graphics System, Version 2.0*, Schrödinger: 2010.
4. *Avogadro: an open-source molecular builder and visualization tool.*, 1.2.0.
5. Hanwell, M. D.; Curtis, D. E.; Lonie, D. C.; Vandermeersch, T.; Zurek, E.; Hutchison, G. R. Avogadro: an advanced semantic chemical editor, visualization, and analysis platform. *J. Cheminform.* **2012**, *4*, 17. DOI: 10.1186/1758-2946-4-17
6. Pettersen, E. F.; Goddard, T. D.; Huang, C. C.; Couch, G. S.; Greenblatt, D. M.; Meng, E. C.; Ferrin, T. E. UCSF Chimera—A visualization system for exploratory research and analysis. *J. Comput. Chem.* **2004**, *25*, 1605–1612. DOI: 10.1002/jcc.20084
7. Legault, C. Y. *CYLview 2.0*, Université de Sherbrooke: 2020.
8. Becke, A. D. Density-functional thermochemistry. III. The role of exact exchange. *J. Chem. Phys.* **1993**, *98*, 5648-5652. DOI: 10.1063/1.464913
9. Grimme, S.; Antony, J.; Ehrlich, S.; Krieg, H. A consistent and accurate ab initio parametrization of density functional dispersion correction (DFT-D) for the 94 elements H-Pu. *J. Chem. Phys.* **2010**, *132*, 154104. DOI: 10.1063/1.3382344
10. Zhao, Y.; Truhlar, D. G. The M06 suite of density functionals for main group thermochemistry, thermochemical kinetics, noncovalent interactions, excited states, and transition elements: two new functionals and systematic testing of four M06-class functionals and 12 other functionals. *Theor. Chem. Account* **2008**, *120*, 215–241. DOI: 10.1007/s00214-007-0310-x
11. Chai, J.-D.; Head-Gordon, M. Long-range corrected hybrid density functionals with damped atom–atom dispersion corrections. *Phys. Chem. Chem. Phys.* **2008**, *10*, 6615–6620. DOI: 10.1039/B810189B
12. Grimme, S. Semiempirical GGA-type density functional constructed with a long-range dispersion correction. *J. Comput. Chem.* **2006**, *27*, 1787–1799. DOI: 10.1002/jcc.20495
13. Weigend, F. Accurate Coulomb-fitting basis sets for H to Rn. *Phys. Chem. Chem. Phys.* **2006**, *8*, 1057–1065. DOI: 10.1039/B515623H
14. Weigend, F.; Ahlrichs, R. Balanced basis sets of split valence, triple zeta valence and quadruple zeta valence quality for H to Rn: Design and assessment of accuracy. *Phys. Chem. Chem. Phys.* **2005**, *7*, 3297–3305. DOI: 10.1039/B508541A
15. Hanaway, D. H.; Kennedy, C. R. *A.V.E.D.A.*, GitHub: 2022.
